# Complete biosynthesis of the fungal alkaloid zinnimidine: Biochemical insights into the isoindolinone core formation

**DOI:** 10.1016/j.jbc.2025.110319

**Published:** 2025-05-31

**Authors:** Ling Luo, Dan Li, Bi Long, Xiaoling Huang, Ying Wan, Hongping Long, Wenxuan Wang, Jing Li, Kangping Xu, Guishan Tan, Xia Yu

**Affiliations:** 1 Xiangya School of Pharmaceutical Sciences, Central South University, Changsha, Hunan, People's Republic of China; 2 Hunan Key Laboratory of Diagnostic and Therapeutic Drug Research for Chronic Diseases, Central South University, Changsha, People's Republic of China; 3 Pharmacy Department, Jiujiang City Key Laboratory of Cell Therapy, JiuJiang NO.1 People's Hospital, Jiujiang, Jingxi, People's Republic of China; 4 Center for Medical Research and Innovation, The First Hospital of Hunan University of Chinese Medicine, Changsha, Hunan, People's Republic of China; 5 Xiangya Hospital of Central South University, Central South University, Changsha, Hunan, People's Republic of China

**Keywords:** biocatalysis, enzyme conservation, fungal natural products, isoindolinone scaffold, total biosynthesis

## Abstract

Isoindolinone scaffold is widely presented in bioactive natural products and serves as a fundamental structure in pharmaceutical compounds. However, the key step of the isoindolinone core formation is not well-defined. In this study, we elucidated the complete biosynthetic pathway of the phytotoxic isoindolinone alkaloid zinnimidine, which was isolated from the cultures of various phytopathogenic fungi of the genus *Alternaria.* This was achieved through a systematic approach that integrated heterologous expression, feeding experiments, and enzymatic characterizations. Enzymes ZinADEF were identified as key catalysts involved in the biosynthesis of isoindolinone scaffold, directing the transformation from the 2-methyl benzoic acid-like tetraketide precursor to form the isoindolinone scaffold, while ZinB and ZinE catalyzes methylation and prenylation modification, respectively. Detailed enzymatic studies of the flavin-dependent oxidoreductase ZinD uncovered the key catalytic processes underlying the conversion from 1,2-benzenediol to the isoindolinone core, accompanied by the generation of hydrogen peroxide. Through enzymatic reactions of ZinD with various amino-containing compounds, porritoxin and several novel isoindolinones were synthesized. The analysis utilizing cblaster highlighted the conservation of ZinADEF genes across different fungi, indicating the existence of common biosynthetic pathways and mechanisms among these fungal species. Additionally, our study explained the late-stage biosynthetic branch details of isoindolinone and isobenzofuranone natural products. Our research unveils the biosynthetic mechanism of fungal isoindolinones, providing a theoretical foundation for the biocontrol of phytotoxins such as zinnimidine, and establishes a foundation for the biosynthesis of isoindolinone alkaloid derivatives.

The isoindolinone scaffold, a benzo-fused γ-lactam, stands out as a versatile framework with a broad range of biological activities and is commonly found in bioactive fungal metabolites (1, 2). Over several decades, this structure has captivated researchers, leading to the discovery of numerous natural products that feature the isoindolinone core across various species. For example, in the genus *Alternaria*, the bicyclic cichorine-type phytotoxic agent zinnimidine (**1**) (3) and the EBV-EA activation inhibitor porritoxin (**2**) (4) have been identified. Asperglactam A from *Aspergillus versicolor* exhibits moderate inhibitory activity against α-glucosidase (5). The tricyclic erinacerin O2, derived from *Hericium erinaceus*, is known for its antiproliferative effects against glioma (6). Spirocollequins B, isolated from *Colletotrichum boninense,* displays antiplasmodial activity (7). Additionally, the tetracyclic asepergillin PZ from *Aspergillus awamori* demonstrates activity against tumor cells (8), and the pentacyclic stachybotrylactam from *Stachybotrys chartarum* (9) is a cytotoxic agent (Fig. 1).

**Figure 1:**
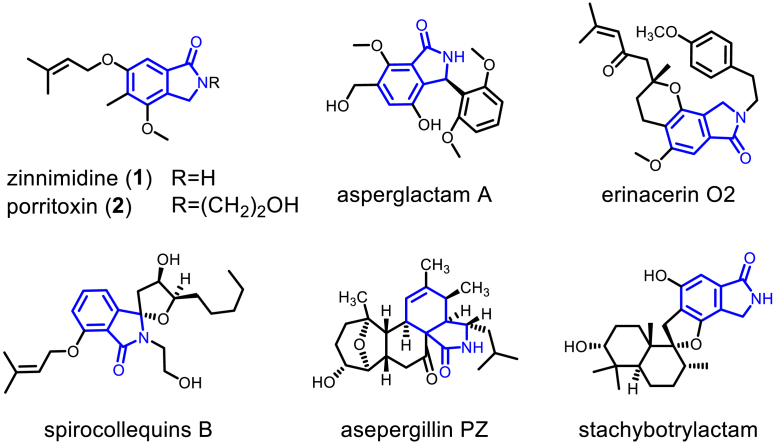
**Examples of natural products containing an isoindolinone scaffold**.

The biosynthetic formation of isoindolinones may involve oxidative events on monocyclic aromatic precursors, according to several studies. For example, Wang *et al.* identified the gene clusters for aspernidine A and cichorine from *Aspergillus nidulans* (*A. nidulans*) and proposed their biosynthetic pathways based on gene deletion results. In their proposed pathways, the oxidoreductase CicC is responsible for the oxidation of a benzo-γ-lactone nidulol, which leads to the formation of isoindolinone cichorine (10), and the choline dehydrogenase encoded by the gene *pkfF* (11) was recommended to be responsible for the oxidation of aspernidine E, followed by a series of steps toward aspernidine A (Fig. 2, *A* and *B*). Some research on the biosynthesis of the triprenyl phenol family of isoindolinones from *Stachybotrys microspora* (SMTP) indicates that the isoindolinone structure could be formed from a nonenzymatic amine conjugation between an *o*-phthalaldehyde precursor pre-SMTP and the corresponding primary amine (12). Recently, cell-free extract assays by Hasumi *et al.* support that the pre-SMTP is generated from a 2-(hydroxymethyl)benzaldehyde intermediate (pri-SMTP) by a flavin-dependent oxidase (pri-SMTP oxidase), which is located outside the SMTP biosynthesis gene cluster (13). However, the proposed processes above were only partially studied, where strict biochemical assays and detailed mechanism discussions for the generation of the isoindolinone scaffold remain lacking. Zinnimidine (**1**) and porritoxin (**2**) are representative phytotoxins isolated from the cultures of the genus *Alternaria*, exhibiting inhibitory activity on the growth of some plant seedlings (3). Studying the biosynthesis of these compounds is crucial for understanding their mechanisms and potential applications in biocontrol. In this study, we investigated the total biosynthesis of a fungal isoindolinone zinnimidine (**1**) from *Alternaria alternata* (*A. alternata*). By combining heterologous expression, feeding experiments, and enzymatic characterization, we systematically characterized the total biosynthetic pathway of zinnimidine and revealed the conservation of ZinADEF genes across diverse fungi, providing comprehensive insight into the natural formation of the isoindolinone scaffold. Additionally, the late-stage biosynthetic branch details for forming isoindolinone and isobenzofuranone-type natural products were explained.

**Figure 2:**
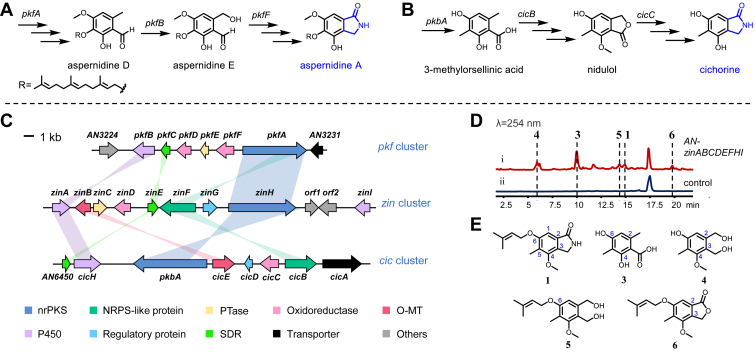
**Gene clusters and the proposed biosynthetic pathway for isoindolinones.***A*, proposed biosynthetic pathway for aspernidine A. *B*, proposed biosynthetic pathway for cichorine. *C*, gene cluster comparison between *zin* and *pkf*, *cic* clusters at the protein level. *D*, HPLC analysis of heterologous reconstitution of the *zin* cluster in *A. nidulans* A1145. *E*, compounds identified from the heterologous expression of *zinABCDEFHI* in *A. nidulans* A1145.

## Results and discussion

### Genome mining and identification of isoindolinone alkaloids

Upon a thorough examination of the genomic sequence of *A. alternata*, a specific gene cluster containing a non-reducing polyketide synthase (nrPKS)-encoding gene gained our attention, which was later named as *zin* cluster. This cluster comprises genes encoding an nrPKS ZinH, a P450 monooxygenase ZinA, an NRPS-like protein ZinF, and an O-methyltransferase ZinB. These enzymes share amino acid sequence identities ranging from 40.6% to 57.6% with PkbA/CicH/CicB/CicE, which are reported to be involved in the biosynthesis of cichorine (10) (Fig. 2*B* and Table S5). Moreover, the *zin* cluster contains seven additional genes, including genes encoding a P450 monooxygenase ZinI, a prenyltransferase ZinC, a flavin-dependent oxidoreductase ZinD, a short-chain dehydrogenase ZinE, a regulatory protein ZinG, a heterokaryon incompatibility protein ORF1, and a protein ORF2 predicted with unknown function (Fig. 2*C* and Table S4). These observations suggest that the *zin* gene cluster is also engaged in the biosynthesis of isoindolinone alkaloids, with products differing from those of previously reported gene clusters.

To identify metabolites derived from the *zin* cluster, we initially introduced eight enzyme-encoding genes (*zinABCDEFHI*) into *A. nidulans* A1145 for heterologous expression. Metabolite analysis of the *A. nidulans* A1145 transformants revealed the presence of five new metabolites (**1, 3**–**6**) in comparison to *A. nidulans* A1145 expressing empty vectors (Fig. 2*D*). All five compounds were isolated for NMR analysis and quickly identified as zinnimidine (**1**) (14) (Figs. S25, S26, and Table S6), 3-methylorsellinic acid (**3**) (15) (Figs. S5, S28, S29 and Table S8), 5-hydroxy-3-methoxy-4-methyl-1,2-benzenedimethanol (**4**) (14) (Figs. S6, S30, S31 and Table S9), zinniol (**5**) (14) (Figs. S7, S32, S33 and Table S10), and 6-[(3′,3′-dimethylallyl)oxy]-4-methoxy-5-methylphthalide (**6**) (16) (Figs. S34, S35, and Table S11), according to comparisons of their NMR spectra with literature data (Fig. 2*E*). The phytotoxin zinnimidine (**1**) exhibits an isoindolinone alkaloid structure (3), confirming the functionality of the *zin* gene cluster as an isoindolinone alkaloid biosynthetic gene cluster, as initially hypothesized. Based on the relationships of the structure of these compounds, **6** or **1** is hypothesized to represent the final product of this gene cluster. Starting from the structurally simplest nrPKS product **3**, a series of conversions, including prenylation, O-methylation, methyl hydroxylation, carboxyl reduction, and hydroxyl oxidation events, are expected during the biosynthesis of the final compound.

To validate our speculation, we initially expressed the nrPKS gene *zinH* in *A. nidulans* A1145 alone, which indeed led to the biosynthesis of compound **3** (Fig. S20). Subsequently, we introduced all isolated metabolites **1**, **3** to **6** to *A. nidulans* A1145 harboring the remaining seven genes in the pathway, excluding *zinH* (AN-*zinABCDEFI*). The supplementation of **3** led to the production of **5**, while feeding **5** resulted in the generation of **1**, thereby confirming that **3** is indeed a precursor for the biosynthetic pathway (Fig. S21). Feeding of compounds **4**, **6**, and **1** could also facilitate the production of **1** (Fig. S21). Therefore, we concluded that compounds **3** to **6** act as biosynthetic precursors for compound **1**. The predicted biosynthetic pathway for cichorine suggested the presence of a gene cluster in *A. nidulans* capable of modifying compound **3** (10). To avoid interference from enzymes in *A*. *nidulans*, we used the *Escherichia coli* (*E. coli*)/*Saccharomyces cerevisiae* (*S. cerevisiae*) system for conducting *in vivo* feeding and *in vitro* enzyme reaction studies to elucidate the roles of genes within the *zin* gene cluster.

### Biochemical reconstruction of the biosynthesis of zinnimidine

**Figure 3:**
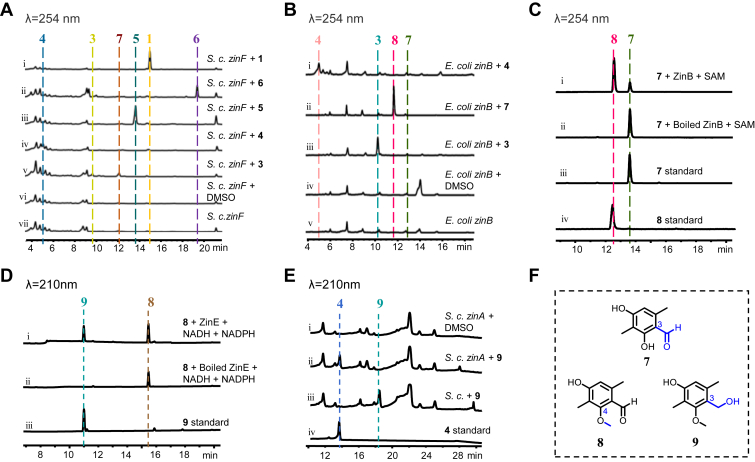
**Verification of the functions of ZinF, ZinB, ZinE, and ZinA.***A*, HPLC analysis of the feeding experiments catalyzed by *S. cerevisiae zinF*. *B*, HPLC analysis of the feeding experiments catalyzed by *E. coli zinB*. *C*, HPLC analysis of *in vitro* assays of ZinB with **7**. *D*, HPLC analysis of *in vitro* assays of ZinE with **8**. *E*, HPLC analysis of the feeding experiments catalyzed by *S. cerevisiae zinA*. *F*, structure of compounds **7**–**9**.

ZinF is an NRPS-like protein encoding A-T-R domains, homologous to CicB (41.20%) in the *cic* cluster (Table S5), which was proposed to be involved in the biosynthesis of downstream products of compound **3** (10). To determine the function of ZinF, we performed feeding experiments by feeding **3** to the culture of *S. cerevisiae* strain expressing *zinF* (*S. c. zinF*). The consumption of **3** and the production of a new product **7** were observed, while feeding compounds **1** and **4** to **6** did not result in the detection of any additional products (Fig. 3*A*). Detailed analysis of the ^1^H and ^13^C NMR spectra revealed that **7** is a derivative of **3,** in which the carboxyl group at the C-3 position of compound **3** has been reduced (17) (Figs. 3*F*, S36, S37, and Table S12). These results demonstrated that ZinF activates and reduces the carboxylic acid group in **3** to yield product **7** containing an aldehyde moiety.

Based on their structure, **4** is speculated to be a downstream product from **7** in the biosynthetic pathway. Subsequently, our research was conducted on elucidating the transformation from **7** to **4**. It is hypothesized that the conversion from **7** to **4** involves processes such as phenolic methylation, hydroxylation, and aldehyde reduction, presumed to correspond to ZinB (methyltransferase), ZinA or ZinI (cytochrome P450 enzyme), and ZinE (short-chain dehydrogenase), respectively. We conducted heterologous expression of these enzymes in *E. coli* or *S. cerevisiae* and performed compound feeding experiments. The results revealed that feeding **7** to the culture of *E. coli* strain expressing *zinB* (*E. coli zinB*) led to the production of **8** (Fig. 3*B*), which was identified to be the 4-O-methylated product of **7** (18) (Figs. 3*F* and S38, and Table S13). No additional products were detected when compounds **3** and **4**, which also contain the phenol group similar to **7**, were fed to the culture of *E. coli zinB* (Fig. 3*B*). To further elucidate the role of ZinB, *in vitro* assays were conducted using recombinant his_6_-tagged ZinB purified from the culture of *E. coli zinB* (Fig. S4). HPLC analysis showed that incubation of ZinB with **7** in the presence of SAM resulted in the production of **8** (Fig. 3*C*), confirming the specific role of ZinB in catalyzing the methylation of compound **7**. Subsequently, we conducted individual enzymatic reactions of **8** with three distinct enzymes, *i.e.* his_6_-tagged ZinE purified from the *E. coli* strain expressing *zinE* (*E. coli zinE*), and P450 enzymes ZinA or ZinI microsomes extracted from the *S. cerevisiae* strain expressing *zinA* or *zinI* (*S. c. zinA* or *S. c. zinI*). HPLC analysis revealed that only in the reaction with ZinE, compound **8** generated a new product, designated as compound **9** (Fig. 3*D*). Integration of ^1^H NMR, ^13^C NMR, and HMBC spectra along with HR-MS analysis verified that compound **9** represents the C-3 aldehyde reduction product derived from compound **8** (Figs. 3*F*, S9, S39–S43, and Table S14). We then introduced **9** into the culture of *S. c. zinA* or *S. c. zinI*. HPLC analysis revealed that the transformation mediated by *S. c. zinA* reinstated the biosynthesis of compound **4** (Fig. 3*E*), indicating that the P450 enzyme ZinA catalyzes the hydroxylation of the C2- methyl group of **9** to generate **4** (Figs. S6, S30, S31, and Table S9). In subsequent experiments for ZinI, compounds **1** to **8** were also fed to *S. c. zinI*. Since no additional product was detected, we concluded that ZinI is not involved in the biosynthesis of zinnimidine and derivatives. Thus, we have specified the reconstruction process from the nrPKS product **3** to the pathway intermediate **4**. The evidence substantiates that nrPKS ZinH biosynthesizes **3**, which is reduced by ZinF to produce **7**. Then the methyltransferase ZinB catalyzes the 4-O-methylation resulting in the formation of **8**. Compound **8** is reduced to **9** by ZinE through ketoreduction, followed by C2- hydroxylation catalyzed by the P450 enzymes ZinA to generate **4**. Interestingly, it was observed that the methylation of the phenolic hydroxyl group of **7** precedes the reduction of the aldehyde group, probably to prevent the formation of the active *ortho*-quinone methides (*o*-QM) intermediate, which is known to participate in a broad range of aromatic coupling reactions.

**Figure 4:**
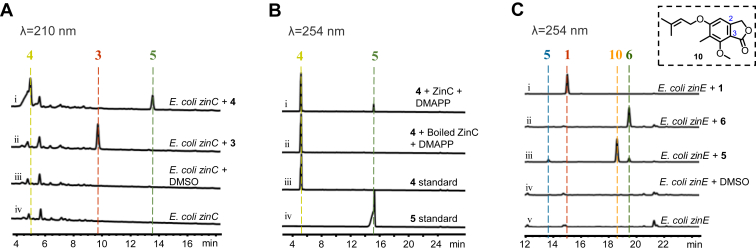
**Verification of the functions of ZinC and ZinE.***A*, HPLC analysis of the feeding experiments catalyzed by *E. coli zinC*. *B*, HPLC analysis of *in vitro* assays of ZinC with **4**. *C*, HPLC analysis of the feeding experiments catalyzed by *E. coli zinE*.

Compound **5** is a derivative resulting from prenylation at the phenolic hydroxyl group of **4**, suggesting that it is likely biosynthesized through a prenyltransferase ZinC-catalyzed reaction of **4**. Experimental evidence from substrate feeding of **4** to *E. coli* expressing *zinC* (*E. coli zinC*) and *in vitro* enzyme reactions of **4** with his_6_-tagged ZinC both support the role of ZinC in catalyzing the conversion of **4** to produce the 6-O-prenylated product **5** (Fig. 4, *A* and *B*). The catalysis by ZinC is substrate-specific, as no product was detected when **3** was used as the substrate in the feeding experiment (Fig. 4*A*). In the feeding experiments of *E. coli zinE*, the supplement of compound **5** as a substrate yielded compound **6** and a new product **10** (Fig. 4*C*). By comparing ^1^H and ^13^C NMR signals from the literature (16), **10** was confirmed as a benzofuranone compound that likely undergoes lactonization following the carboxylation of the C3- hydroxymethyl group of **5** (Figs. 4*C*, S44, S45, and Table S15). The structure of **10** differs from **6** by its isomeric nature, featuring a reversed lactone structure. The biosynthesis of compounds **6** and **10** mediated by *E. coli zinE* can be explained as a simultaneous reverse reaction to the previously observed aldehyde reduction reaction. The variation lies in the substitution of the aldehyde at either C-2 or C-3 produced during dehydrogenation. Subsequent spontaneous reactions lead to the creation of a hemiacetal and air oxidation, culminating in lactone formation in the generation of **10** and **6**.

**Figure 5:**
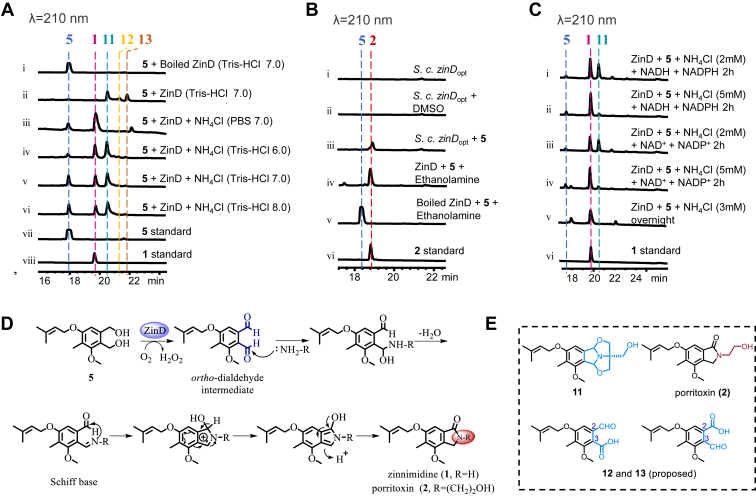
**Detailed characterization of ZinD.***A–B*, *in vitro* and *vivo* studies of ZinD with **5**. Enzyme reactions were in the presence of NADH, NADPH, FAD, and FMN. *C*, *in vitro* assays of ZinD with **5** under different cofactors and NH_4_Cl concentrations (in the presence of FAD and FMN). *D*, proposed formation mechanisms from **5** to **1** and **2**. *E*, structure of compounds **2**, **11**–**13**.

However, the formation mechanism of the isoindolinone scaffold in zinnimidine (**1**) remains undisclosed. Considering the 42.7% protein sequence similarity between the flavin-dependent oxidoreductase ZinD and pri-SMTP oxidase (13), it is speculated that ZinD might play a crucial role in the formation of the isoindolinone ring in zinnimidine (**1**). Unfortunately, its overexpression in *S. cerevisiae* resulted in insufficient quantities of his_6_-tagged ZinD. Therefore, codon optimization was conducted for ZinD to enhance protein yield. The optimized his_6_-tagged ZinD (*zinD*_opt_) was successfully purified from *S. cerevisiae* after codon optimization. Subsequent incubation of the purified ZinD with **5** under various reaction conditions at 30 °C overnight (Fig. 5*A*) revealed the formation of **1**, along with three additional products **11**, **12**, and **13**. This result further confirmed the pivotal role of ZinD as a key enzyme in the biocatalytic synthesis of the isoindolinone ring in zinnimidine (**1**). Additionally, we assessed the conversion yield of **1** under different pH conditions, observing a higher yield using Tris-HCl buffer at pH 6.0 (Fig. 5*A*, iv), while lower yields were obtained using Tris-HCl buffer at pH 7.0 or 8.0 (Fig. 5*A*, v and vi). Importantly, the absence of NH_4_Cl in the enzyme reaction precluded the generation of **1**. This confirmed the critical role of the nitrogen source in the catalytic production of **1** by ZinD.

### Mechanistic study of ZinD and its applications in the production of isoindolinone derivatives

Compared to the NH_4_Cl-free assay of ZinD, the addition of NH_4_Cl resulted in the production of **1** and a decrease in products **11**, **12**, and **13** (Fig. 5*A,* i, ii, and v). To verify whether **11**, **12**, and **13** serve as biosynthetic precursors of **1**, each compound was incubated with purified his_6_-tagged ZinD in the presence of NH_4_Cl. However, no product formation was detected, suggesting that **11**, **12**, and **13** are not intermediates in the enzymatic process but rather by-products. The molecular formula of compound **11** was determined as C_19_H_25_NO_5_ based on the HR-ESI-MS data (Fig. S10). The four additional carbons in **11** were hypothesized to be obtained from Tris component in the Tris-HCl buffer utilized during the enzymatic reaction. Structural analysis from 2D NMR spectra (Figs. S46–S50 and Table S16) confirmed the structure of compound **11** as a di-acetal structure arising from the interaction between Tris and **5** (Detailed structure elucidation is available in the Experimental procedures). The structure of **11** was further validated *via* ^13^C NMR chemical shift calculation conducted by Density Functional Theory (DFT) calculations using Gaussian (19) (Table S17).

Based on the acetal structure identified in compound **11** (Fig. 5*E*), it is hypothesized that ZinD catalyzes the dehydrogenation of the 1,2-benzenedimethanol backbone within compound **5** to generate a highly reactive *ortho*-dialdehyde intermediate. This intermediate subsequently undergoes a spontaneous reaction with Tris to form **11** (Fig. S23). To confirm the origin of the Tris-like moiety, we substituted Tris-HCl buffer with PBS buffer for both the enzyme purification and enzymatic reactions of ZinD and observed cessation of compound **11** production. Instead, only compound **1** accompanied by minor quantities of compounds **13** and **12** was detected in the HPLC analysis (Fig. 5*A*, iii). Due to the limited yields of **12** and **13**, the quantity obtained was insufficient for NMR analysis. HR-ESI-MS analysis identified the molecular formula C_15_H_18_NO_5_ for both **12** and **13** (Figs. S11 and S12). Additionally, both mass spectra exhibited a peak corresponding to a 44 Da reduction from the molecular ion peak. This suggests that compounds **12** and **13** contain an *ortho*-carboxybenzaldehyde moiety (Fig. 5*E*), with variations in the positioning of the carboxyl group at either C-2 or C-3. The formation of compounds **12** and **13** might be explained as a result of the air oxidation of the *ortho*-dialdehyde intermediate. Conversely, in the presence of a nitrogen source, the *ortho*-dialdehyde intermediate undergoes spontaneous reactions with primary amine groups to efficiently and rapidly generate isoindolinone compounds. Due to the high reactivity of the *ortho*-dialdehyde intermediate within the enzymatic reaction system, we were unable to isolate it for the identification of non-enzymatic amine conjugation. Instead, we utilized *o*-phthalaldehyde (OPA) standard to react with NH_4_Cl, and the production of isoindolin-1-one was confirmed by HPLC analysis (Fig. S22). This observation led us to conclude that compound **5** undergoes oxidation by ZinD to generate an *ortho*-dialdehyde structure, which is then subjected to react with amine donors to form indolinones.

To test cofactor dependence of ZinD, we conducted enzymatic assays with the addition of NADH/NADPH or NAD^+^/NADP^+^, as well as without these cofactors. HPLC analysis validated the consistent production of compound **1** in reaction systems containing either oxidized or reduced cofactors (Fig. 5*C*, i-v). The experimental results also indicated that as the concentration of the nitrogen source increases, the yield of isoindolinone correspondingly increases (Fig. 5*C*, i-iv). To further study the mechanism of the ZinD-catalyzed benzyl alcohol oxidation, especially to verify the proposed by-product hydrogen peroxide formed from oxygen, we used a colorimetric hydrogen peroxide assay kit to detect the hydrogen peroxide level from the *in vitro* enzyme reaction. In the presence of NH_4_Cl as a nitrogen source and without additional cofactors, hydrogen peroxide was detected by the kit in the assay with active ZinD, in which the conversion of compound **5** to **1** was observed, in significant contrast to the inactivated ZinD negative control group (Fig. S24). The detection of hydrogen peroxide from the ZinD catalyzed reaction clearly indicated that flavoenzyme ZinD acts as an oxidase that converts *o*-phthalyl alcohol **5** to the corresponding *o*-pthalaldehyde **1** with the production of hydrogen peroxide, as the by-product from the reducing half-reaction.

Subsequently, we repeated the previous yeast feeding experiment using compound **5** as the substrate and fed it to the *S. cerevisiae* strain expressing *zinD*_opt_, resulting in the observation of a new product **2** (Fig. 5*B*, i-iii). The product was identified as porritoxin, an isoindolinone natural product isolated from *Alternaria porri,* through NMR analysis (20) (Table S7, Figs. 5*E*, S8, and S27). We hypothesized that **2** was formed through a spontaneous reaction between ZinD-catalyzed *ortho*-dialdehyde intermediate and a primary amine with an ethanol side chain present either in the medium or within the yeast cells. Upon replacing the nitrogen source NH_4_Cl in the enzymatic assays catalyzed by ZinD with ethanolamine, we successfully verified our hypothesis by detecting the formation of **2** by HPLC analysis (Fig. 5*B*, iv-v).

**Figure 6:**
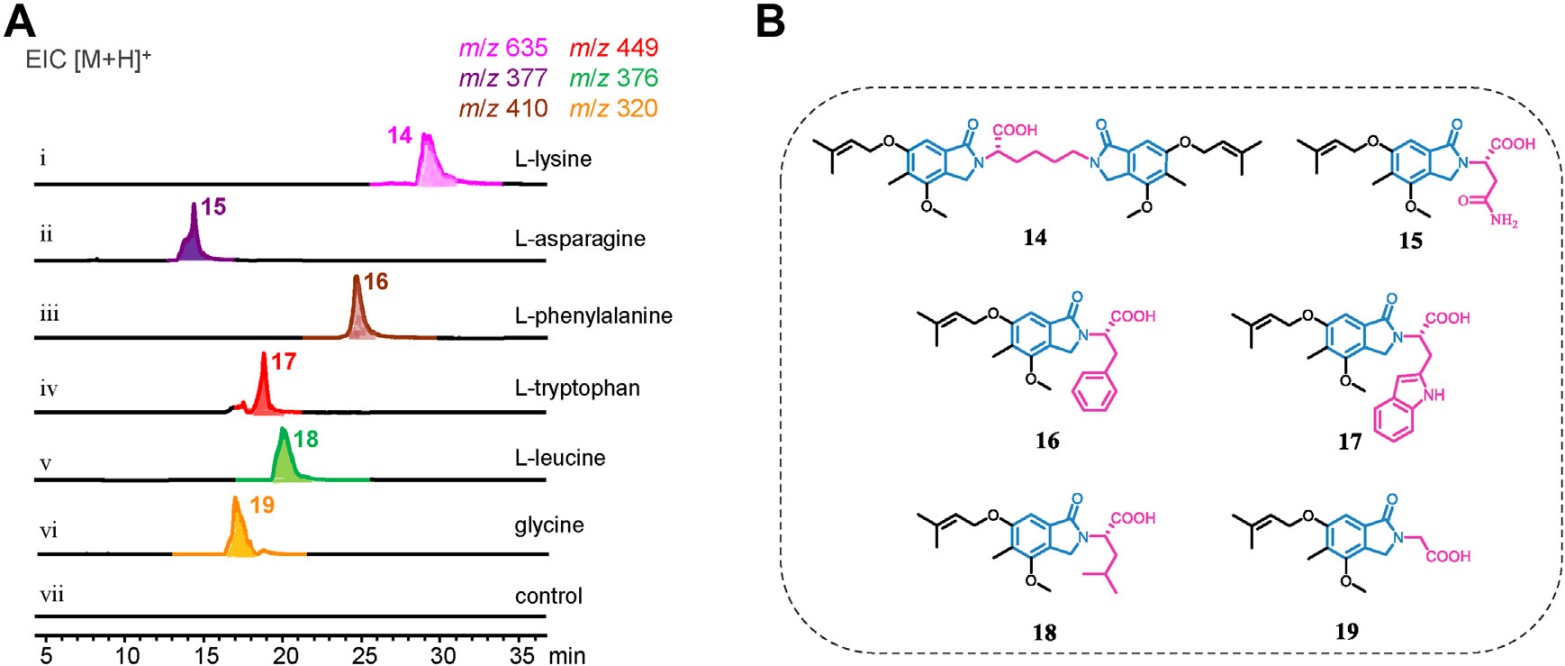
**Using ZinD and various amino-containing compounds to synthesize several new isoindolinones.***A*, LC-MS analysis of novel isoindolinones formed by the catalysis of **5** by ZinD in the presence of different amino acids. *B*, structure of compounds **14** to **19**.

According to the experimental results, we proposed the following mechanism for the formation of the isoindolinone ring in zinnimidine (**1**). Under the catalysis of ZinD, the *ortho*-diol structures in **5** generate reactive *ortho*-dialdehyde intermediates, which then react with nitrogen sources to form relatively stable Schiff base intermediates through a dehydration reaction. Finally, the carbonyl group undergoes nucleophilic addition with the imine to generate a cationic intermediate. Through electron transfer, the H^+^ dissociates to form isoindolinone compounds (21) (Fig. 5*D*). Due to the high reactivity of the *ortho*-dialdehyde structure towards primary amines, we hypothesize that compounds with primary amine moieties can generate isoindolinone compounds with diverse structures through enzymatic and non-enzymatic processes, with compound **5**. Therefore, we selected a variety of compounds containing primary amine moieties as nitrogen sources to participate in the *in vitro* enzymatic reactions with ZinD and compound **5**. The results showed that L-lysine, L-asparagine, L-phenylalanine, L-tryptophan, L-leucine, and glycine reacted with **5** to generate new products **14**, **15**, **16**, **17**, **18**, and **19,** respectively (Fig. 6, *A* and *B*). HR-MS analysis confirmed the molecular formula C_36_H_46_N_2_O_8_, C_19_H_24_N_2_O_6_, C_24_H_27_NO_5_, C_26_H_28_N_2_O_5_, C_21_H_29_NO_5_, and C_17_H_21_NO_5_ for compounds **14**, **15**, **16**, **17**, **18**, and **19,** respectively (Figs. S13–S18). The decrease in molecular formula of H_6_O compared to the combined molecular formulas of **5** and the respective amino acid suggests the formation of the isoindolinone structure in association with the amino acid. To further confirm the formation of the isoindolinone ring, we scaled up the enzymatic reaction using L-phenylalanine as the nitrogen source. HR-MS and NMR analysis confirmed the presence of the isoindolinone structure in **16** (Figs. S15, S51, and Table S18).

### Comparative analysis of isoindolinone biosynthetic gene clusters (BGCs) in various fungi

Compared to the *zin* cluster, the two previously reported isoindolinone BGCs (*pkf* and *cic*) both contain a central nrPKS encoding gene. In contrast, instead of containing a homolog to *zinF*, which is the A-T-R domains that catalyze the reduction of carboxylic acid in the structure to form 2-methylbenzaldehyde, the *pkf* cluster involves an nrPKS gene *pkfA*, which comprises a C-terminal R domain. While the *cic* cluster has both nrPKS PkbA and A-T-R domain containing CicB. Subsequently, both the *pkf* and *cic* clusters have homologs of *zinA* and *zinE* capable of producing lactone or diol products. Interestingly, there is no homolog of *zinD* in the *pkf* and *cic* clusters, indicating that there must be other genes within these clusters catalyzing alcohol to aldehyde conversion.

**Figure 7:**
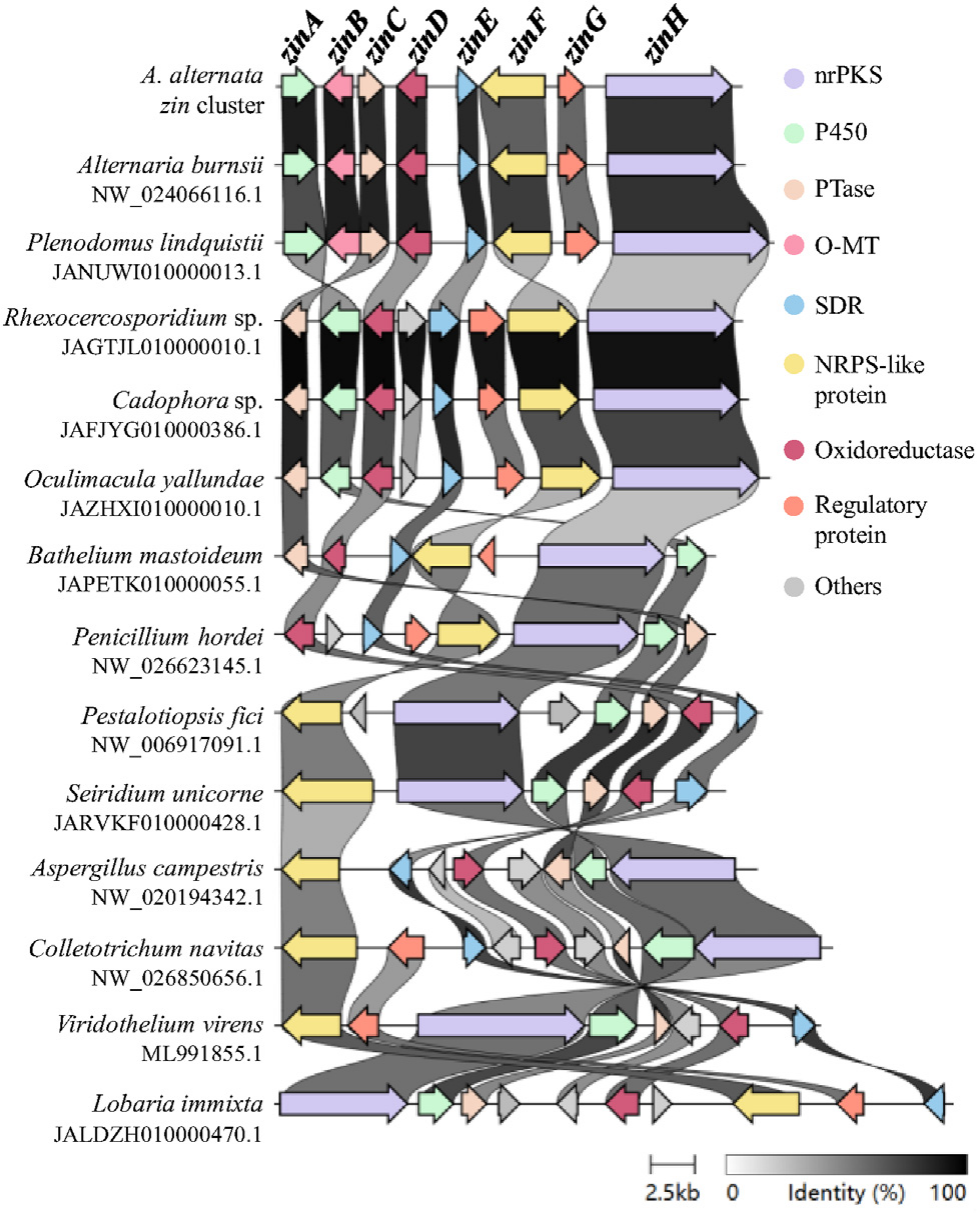
**Selected examples of BGCs with homologies to ZinADEF from fungal strains of 13 different genera aligned using clinker**.

As the differences between the *zin* and *cic*/*pkf* pathway to form isoindolinone, we wonder if *zin* pathway is an isolated case or a general route. Utilizing the sequence of isoindolinone scaffold-forming cassette ZinADEF as a query, we employed the cblaster tool on the CAGECAT server (22) to search for biosynthetic gene clusters harboring homologs to ZinADEF. Our search algorithm detected homologs of the *zin* cluster across various classes of fungi, including *Eurotiomycetes*, *Leotiomycetes*, *Dothideomycetes*, *Sordariomycetes,* and so on. The investigation of homologous biosynthetic gene clusters selected from fungal strains of 13 different genera using clinker tool (23) revealed a high degree of conservation of the ZinADEF genes (Fig. 7). This conservation highlights the consistent involvement of ZinADEF genes in the biosynthesis of the isoindolinone scaffold across various fungi, thereby suggesting the presence of shared biosynthetic mechanisms among these fungal species.

**Figure 8:**
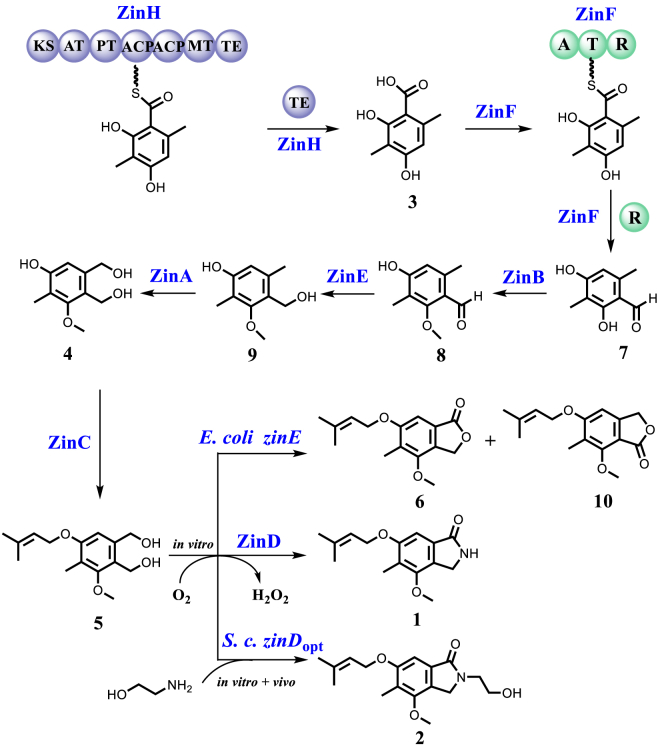
**Biosynthetic pathway of isoindolinones zinnimidine (1) and porritoxin (2), along with isobenzofuranones 6 and 10**.

Interestingly, the biosynthesis of isoindolinones (**1**, **2**) and isobenzofuranones (**6**, **10**) both originate from a common tetraketide precursor **3** and share a common 2-methylbenzaldehyde-like intermediate (Fig. 8). In subsequent biosynthetic steps, the aldehyde moiety is selectively reduced to a hydroxymethyl group by an SDR enzyme (*e.g.,* ZinE) at an appropriate stage, while the methyl group is also converted to a hydroxymethyl group by a P450 enzyme (*e.g.,* ZinA). This 1,2-phenylenedimethanol structure (*e.g.,* **5**) serves as the final common intermediate for isoindolinones and isobenzofuranones. The reverse reaction catalyzed by a ZinE homology SDR enzyme can convert one of the hydroxymethyl groups back to an aldehyde, leading to the formation of 2-(hydroxymethyl)benzaldehyde, which spontaneously undergoes cyclization and dehydrogenation to yield isobenzofuranone (*e.g.,* **6** and **10**). Acting as a crucial catalyst in the isoindolinone pathway, the ZinD-like flavin-dependent oxidoreductase catalyzes the formation of the reactive phthalaldehyde structure from **5**, which readily reacts with amino-containing compounds, spontaneously generating distinct isoindolinones. Through rigorous biochemical experiments, this study elucidates the late-stage biosynthetic details of isoindolinone and isobenzofuranone natural products.

## Conclusion

This research performed a comprehensive elucidation of the biosynthetic pathway for the formation of the isoindolinone scaffold in nature. Focusing on the isoindolinone zinnimidine, a phytotoxin isolated from the cultures of a variety of phytopathogenic fungi from the genus *Alternaria*, we revealed the key role of the enzymes ZinADEF in the biosynthesis of the isoindolinone scaffold. These enzymes catalyze the transformation from the 2-methylbenzoic acid-like tetraketide precursor to form the isoindolinone scaffold. Detailed enzymatic analyses of ZinD uncovered the catalytic oxidation process on the 1,2-benzenedimethanol intermediate, which led to the spontaneous formation of the isoindolinone core when ammonia is present. Application of ZinD in the supply of various amino-containing compounds resulted in the synthesis of several new isoindolinones. The bioinformatic analysis highlighted the consistent presence of ZinADEF in diverse fungi, suggesting shared biosynthetic pathways and mechanisms across these fungal species. Additionally, this study explained the late-stage biosynthetic branch details involved in generating isoindolinone and isobenzofuranone natural products. This research unveils the biosynthetic mechanism of fungal isoindolinones and establishes a foundation for the biosynthesis of isoindolinone alkaloid derivatives. Furthermore, elucidating the biosynthetic mechanism of the phytotoxic zinnimidine provides a theoretical basis for the development of biocontrol methods.

## Experimental procedures

### Strains and culture conditions

*A. alternata* ACCC 36022 was obtained from Agricultural Culture Collection of China (ACCC) and cultured at 28 °C. *A. nidulans* A1145 was cultured at 37 °C. *A. nidulans* A1145 was grown on Solid Czapek Dox (CD) medium for sporulation or on Czapek Dox-starch (CD-ST) medium for compound production and RNA isolation. *S. cerevisiae* RC01 was used for *in vivo* homologous recombination and heterologous expression of biosynthetic genes. *E. coli* strains DH5α and XL1 Blue were used for cloning, while the strain BAP1 was used for expression.

### General molecular biology methods

Genomic DNA (gDNA) and RNA from *A. alternata* were prepared using ZYMO ZR Fungal/Bacterial DNA Kit (Zymo Research) and Quick-RNA Fungal/Bacterial Miniprep (Zymo Research) according to the manufacturer’s protocol, respectively. Polymerase chain reactions for cloning were performed using PrimeSTAR Max DNA Polymerase (Takara). The plasmids for *S. cerevisiae* or *A. nidulans* A1145 expression were constructed by the yeast homologous recombination method and rescued using the Zymoprep Yeast Miniprep Kit (Zymo Research). Complementary DNA (cDNA) was synthesized with HiScript III First-Strand cDNA Synthesis Kit (Vazyme). The agarose gel images of RNA and cDNA were developed and captured by a camera.

### Plasmid construction

The plasmids for *A. nidulans* A1145 expression were constructed by the yeast homologous recombination method. The fragments of the gene *zinA*, *zinB*, *zinC*, *zinD*, *zinE*, *zinF*, *zinH* or *zinI*, each with its original terminator were amplified from the gDNA of *A. alternata* by using primer pairs AN-zinA-F/AN-zinA-R, AN-zinB-F/AN-zinB-R, AN-zinC-F/AN-zinC-R, AN-zinD-F/AN-zinD-R, AN-zinE-F/AN-zinE-R, AN-zinF-F/AN-zinF-R, AN-zinH-F1/AN-zinH-R1/AN-zinH-F2/AN-zinH-R2, or AN-zinI-F/AN-zinI-R, respectively. The promoter *glaA, gpdA,* and *amyB* were amplified by using primers glaA-F/glaA-R, gpdA-F/gpdA-R, and AmyB-F/AmyB-R from vectors pYTU, pYTR, and pYTP, respectively. In plasmid p35-01, *glaA*, *gpdA*, and *amyB* promoters were used for *zinH*, *zinE*, and *zinA*, respectively. Plasmid p35-02 contains *zinB* with promoter *glaA,* and *zinC* with promoter *gpdA*, while *zinF* with promoter *amyB*, *zinD* with promoter *gpdA*, and *zinI* with promoter *glaA* were recombinated into p35-03. To obtain the fungal expression vectors with *zinE* and *zinA*, p35-01 was digested by NheI to remove part of the *zinH* gene fragment, then the backbone was re-ligated to give p35-04. To construct the plasmids for overexpression in *S. cerevisiae*, the PCR fragment containing the entire coding sequence of *zinA*, *zinD*, *zinF*, or *zinI* was amplified from cDNA by using primer pair zinA-F/zinA-R, zinD-F/zinD-R, zinF-F/zinF-R or zinI-F/zinI-R, respectively. Next, primers SC-zinD-F/SC-zinD-R, SC-zinF-F/SC-zinF-R, SC-zinA-F/SC-zinA-R, and SC-zinI-F/SC-zinI-R were used to amplify the above PCR product to obtain the target fragment for co-transforming into yeast with NdeI/PmlI-digested pXW55 vector, or NdeI/PmeI-digested pXW06 vector to give vectors pZY20 to 21 and pZY23 to 24. To construct the *E. coli* expression vectors pZY26 to 27 and pZY29, the PCR fragment containing the entire coding sequence of *zinB, zinC*, or *zinE* was amplified from cDNA by using primer pair zinB-F/zinB-R, zinC-F/zinC-R or zinE-F/zinE-R, respectively. Primers pCold-zinB-F/R, pCold-zinC-F/R, and pCold-zinE-F/R were then used to amplify the above product. Each PCR fragment was ligated into the linearized vector, which was subsequently digested by appropriate restriction enzymes. The NdeI/BamHI-digested fragment for *zinB* was ligated into the NdeI/BamHI-digested linearized pCold to yield the *E. coli* expression vector pZY26. Similar to pZY26, the obtained fragment for *zinC* and *zinE* was inserted into pCold by using NdeI/XbaI sites to give the expression vector pZY27 and pZY29, respectively.

### Protein overproduction and purification of ZinB, ZinC, and ZinE in *E. coli*

To overexpress ZinB, ZinC, and ZinE, the constructed plasmids pZY26, pZY27, and pZY29 were introduced into *E. coli* BAP1, respectively. The overnight precultures were transferred into 1 L liquid LB medium supplemented with 50 μg/ml ampicillin and grown at 37 °C to an absorption at 600 nm of 0.6 to 0.8. Isopropyl thiogalactoside (IPTG) was added to the culture to a final concentration of 0.1 mM. The cells were induced at 16 °C for 16 h, then harvested by centrifugation and resuspended in lysis buffer (10 mM imidazole, 50 mM NaH_2_PO_4_, 300 mM NaCl, pH 8.0) at 2 to 5 ml per gram wet weight. After sonication on ice, the lysate was centrifuged at 13,000×*g* for 45 min at 4 °C. Purification of the soluble His_6_-tagged fusion protein from supernatant was performed by using affinity chromatography with Ni-NTA agarose resin (Qiagen) according to the manufacturer’s instructions. For buffer exchange, the purified protein was loaded on a PD-10 desalting column (GE Healthcare), which had been equilibrated with 50 mM Tris-HCl, pH 7.5, previously. The purified enzyme was checked by SDS-PAGE, and the concentration was measured with the Q5000 micro-volume UV-Vis spectrophotometer (Quawell). The SDS-PAGE gel images of the purified enzyme were developed and captured by a camera.

### Protein overproduction and purification of ZinD in *S. cerevisiae*

*S. cerevisiae* RC01 harboring the plasmid pZY20 or pZY25 was cultured overnight at Uracil Dropout Medium and then transferred to a 2000 ml Erlenmeyer flask containing 500 ml liquid YPD medium, and incubated at 30 °C, 250 rpm for 3 days. Pellets were collected by using a centrifuge and resuspended in lysis buffer (10 mM imidazole, 50 mM NaH_2_PO_4_, 150 mM NaCl, pH 8.0) and lysed on ice by sonication. The lysate was centrifuged at 13,000*g* for 45 min at 4 °C to remove the cellular debris. Recombinant His_6_-tagged fusion protein was purified from supernatant by using affinity chromatography with Ni-NTA agarose resin (Qiagen) according to the manufacturer’s instructions. To exchange the buffer, the purified protein was passed through a PD-10 desalting column (GE Healthcare) pre-equilibrated with 50 mM Tris-HCl or 100 mM PBS at different pH. The eluate was then concentrated using an Amicon centrifugation filter unit (EMD Millipore) to obtain a higher protein concentration. The purified enzyme was checked by SDS-PAGE, and the concentration was measured with the Q5000 micro-volume UV-Vis spectrophotometer (Quawell). The SDS-PAGE gel images of the purified enzyme were developed and captured by a camera.

### *In vitro* assays

The enzyme reaction mixtures for determination of ZinB activities (50 μl) contained 50 mM Tris-HCl (pH 8.0), 5 mM MgCl_2_, 2 mM SAM, compound **7**, and 30 μg purified recombinant protein. Negative controls were performed with inactivated ZinB by boiling for 30 min. The reaction mixtures were incubated at 30 °C overnight and terminated by the addition of 50 μl acetonitrile. The insoluble materials were removed by centrifugation for 15 min at 13,000 rpm, and the supernatant was subjected to HPLC for analysis. For reactions with ZinC, 100 μg zinC, 5 mM CaCl_2,_ 0.5 mM DMAPP, and compound **4** were given in 50 mM Tris-HCl, pH 7.5 at a final volume of 50 μl. The reaction was incubated at 37 °C, then terminated with an equal volume of methanol and treated the same as described for those with ZinB. The incubation mixture of ZinE contained 50 mM PBS, pH 6.4, 5 mM MgCl_2_, 2 mM NADH, 2 mM NADPH, 100 μg protein, and compound **8**. After incubation at 30 °C overnight, the mixture was also quenched by methanol and analyzed by HPLC after removal of the protein precipitate. Enzyme assays for ZinD (50 μl) in Figure 5*A*, i, ii, and iv-vi contained 50 mM Tris-HCl at different pH (6.0, 7.0, or 8.0), 2 mM NADPH, 2 mM NADH, 1 mM FAD, 1 mM FMN, 2 mM NH_4_Cl, 50 μg of the purified recombinant ZinD and compound **5**. 100 mM PBS at pH 7.0 was used for the enzyme assay of ZinD (50 μl) in Figure 5*A*, iii. Enzyme assays for ZinD (50 μl) in Figure 5*B*, iv-v contained 100 mM PBS (pH 7.0), 2 mM NADPH, 2 mM NADH, 1 mM FAD, 1 mM FMN, 4 mM ethanolamine, 50 μg of the purified recombinant ZinD and compound **5**. Enzyme assays for ZinD (50 μl) in Figure 5*C* contained 50 mM Tris-HCl pH 7.0, 2 mM NADPH/NADP^+^ or NADH/NAD^+^ or without them, 1 mM FAD, 1 mM FMN, 2, 3 or 5 mM NH_4_Cl, 100 μg purified recombinant ZinD and compound **5**. Incubations were carried out at 30 °C overnight. After addition of 50 μl methanol and centrifugation, the supernatants were analyzed by HPLC.

### Feeding experiments

Recombinant plasmid pZY23, pZY25, pZY21, or pZY24 was transformed into *S. cerevisiae* using Frozen-EZ Yeast Transformation II (Zymo Research) to obtain *S. cerevisiae zinA*, *S. cerevisiae zinD*_opt_, *S. cerevisiae zinF*, or *S. cerevisiae zinI* strain, respectively. *S. cerevisiae zinA* or *S. cerevisiae zinD*_opt_ strain was cultured overnight in Uracil Dropout Medium. *S. cerevisiae zinI* or *S. cerevisiae zinF* strain was cultured in Tryptophan Dropout Medium. Then, the pre-culture solution was added to the YPD medium and cultured at 30 °C, 250 rpm for 3 days. The fermentation broth was concentrated by centrifugation at 2000 rpm for 15 min at 4 °C. The substrate dissolved in DMSO was added to the corresponding fermentation broth and incubated for an appropriate time. The fermentation broth was extracted with ethyl acetate three times afterwards. Samples were dried and resolved in methanol to be injected into the HPLC for analysis.

Plasmid pZY26, pZY27, or pZY29 was transformed into *E. coli* cells using the heat shock method to obtain *E. coli zinB*, *E. coli zinC*, or *E. coli zinE* strain, respectively. The overnight precultures of *E. coli zinB*, *E. coli zinC*, or *E. coli zinE* strain were transferred into 1 L LB medium supplemented with 50 μg/ml ampicillin. The cells were grown at 37 °C to an absorption at 600 nm of 0.6 to 0.8. IPTG was added to a final concentration of 0.1 mM and the cells were cultivated for a further 16 h at 16 °C for induction. Each culture was concentrated *via* centrifugation, followed by the addition of the substrate dissolved in DMSO. The corresponding fermentation broth was incubated for an appropriate time, then extracted by ethyl acetate for three times afterward. Samples were dried and resolved in methanol to inject into HPLC for analysis.

### Sample analysis and compound isolation

Sample analysis was performed on an Agilent 1200 Series HPLC System (Agilent Technologies) using a Venusil MP C18 column (4.6 × 100 mm, 5 μm, Agela Technologies). Acetonitrile (solvent A) and water (solvent B) were used at a flow rate of 1 ml/min. For the samples of heterologous expression in *A. nidulans* A1145 in Figures 2*D* and S19, and S20, a linear gradient from 5 to 98% (v/v) solvent A was used for 25 min, followed by 98% (v/v) solvent A for 10 min, and then the column was equilibrated with 5% (v/v) solvent A for 5 min. For the samples of substrate feeding for *zinA* in Figure 3*E*, the analysis started with a linear gradient of 2 to 25% (v/v) solvent A for 25 min, 25 to 95% (v/v) solvent A for 10 min, 95% (v/v) solvent A for 10 min and equilibrated with 2% (v/v) solvent A for 7 min. For the samples of enzymatic assays of ZinD in Figure 5, *A*–*C*, a liner gradient from 5 to 95% (v/v) solvent A was used for 35 min, followed by 95% (v/v) solvent A for 10 min, and then the column was equilibrated with 5% (v/v) solvent A for 7 min. Other samples were analyzed with a linear gradient of 10 to 95% (v/v) solvent A for 25 min, 95% (v/v) solvent A for 10 min, and 10% (v/v) solvent A for 5 min. Analysis of the compounds combined with LC-MS using negative or positive mode electrospray ionization was utilized to identify the products. The analysis started with a linear gradient of 5 to 100% (v/v) solvent A for 25 min. Then the column was washed with 100% (v/v) solvent A for 10 min and equilibrated with 5% (v/v) solvent A for 2 min with a flow rate of 0.2 ml/min.

For isolation of compounds **1**, **3** to **6**, the strain *AN-zinABCDEFHI* was grown on CD-ST agar plates (1 L) at 30 °C for 4 to 6 days. The solid medium was cut into small pieces and extracted with ethyl acetate three times. The extracts were concentrated by rotary evaporation and re-dissolved in methanol. Then, the dissolved fragment was separated on a Sephadex LH-20 column eluted with methanol. Fractions containing the target compounds were identified by HPLC analysis and combined for further purification. A semi-preparative column ODS-A C18 column (10 × 250 mm, 5 μm, YMC) was used for isolation of the products, with the same solvents mentioned above at a flow rate of 2 ml/min. For the purification the compounds **1**, **3** to **5**, a linear gradient from 10 to 50% (v/v) solvent A for 20 min, followed by 50 to 56% (v/v) solvent A for 14 min, then the column was washed with 98% (v/v) solvent A for 10 min and equilibrated with 10% (v/v) solvent A for 10 min. For **6**, a liner gradient from 10%-30% (v/v) of solvent A for 10 min was eluted, followed by 30%-95% (v/v) solvent A for 35 min, and then the column was washed with 98% (v/v) of solvent A for 10 min, and equilibrated with 10% (v/v) of solvent A for 10 min.

For isolation of compound **7**, compound **3** was fed to the heterologous strain of *S. cerevisiae* harboring the plasmid pZY21. Compound **7** was purified by HPLC with a linear gradient from 30 to 75% (v/v) solvent A for 26 min, followed by 75 to 95% (v/v) solvent A for 2 min, then the column was washed with 95% (v/v) solvent A for 10 min and equilibrated with 30% (v/v) solvent A for 10 min at a flow rate of 2.5 ml/min. For isolation of compound **10**, compound **5** was fed to the *E. coli* strain harboring the plasmid pZY29. Compound **10** was purified by HPLC with a linear gradient from 5 to 86% (v/v) solvent A for 36 min, followed by 86 to 95% (v/v) solvent A for 2 min, then the column was washed with 95% (v/v) solvent A for 8 min and equilibrated with 5% (v/v) solvent A for 8 min at a flow rate of 3 ml/min. For isolation of compound **8**, compound **7** was fed to the *E. coli* strain harboring the plasmid pZY26. Compound **8** was purified by HPLC with a linear gradient from 5 to 95% (v/v) solvent A for 30 min, followed by 95% solvent A for 8 min, then the column was equilibrated with 5% (v/v) solvent A for 9 min at a flow rate of 2.5 ml/min. For isolation of compound **9**, compound **8** was fed to the *E. coli* overexpression strain harboring the plasmid pZY29. Compound **9** was purified by HPLC with a linear gradient from 5 to 40% (v/v) solvent A for 20 min, followed by 40 to 90% solvent A for 15 min, then the column was equilibrated with 5% (v/v) solvent A for 10 min at a flow rate of 2.5 ml/min.

Compounds **11**, **12**, and **13** were obtained by the large-scale *in vitro* enzymatic reaction of ZinD with **5** in the absence of a nitrogen donor. Compounds **11**, **12,** and **13** were purified by HPLC with a linear gradient from 40 to 68% (v/v) solvent A for 8 min, followed by 68 to 95% solvent A for 12 min, then the column was washed with 95% (v/v) solvent A for 12 min and equilibrated with 40% (v/v) solvent A for 10 min at a flow rate of 2.0 ml/min. Compound **2** was obtained by feeding **5** as substrate to *S. cerevisiae* strain expressing *zinD*_opt_, and the purification conditions of compound **2** were the same as above. To obtain compound **16**, the enzymatic reaction of ZinD was scaled up with L-phenylalanine as the nitrogen source, and the purification conditions were the same as above.

### NMR spectra analysis

^1^H, ^13^C, and 2D NMR spectra were recorded on a Bruker AV400, AV 500, or AV 600 spectrometer with a 5 mm dual cryoprobe (Bruker Corp). Chemical shifts were referenced to the solvent signal of CDCl_3_ at *δ*_C_ 77.16 ppm and δ_H_ 7.26 ppm, acetone-*d*_*6*_ at *δ*_C_ 29.84 ppm, 206.26 ppm and *δ*_H_ 2.05 ppm, CD_3_OD at *δ*_C_ 49.00 ppm and *δ*_H_ 3.31 ppm, DMSO-*d*_*6*_ at *δ*_C_ 39.52 ppm and *δ*_H_ 2.50 ppm. The NMR spectra were processed with MestReNova software.

### H_2_O_2_ level detection

H_2_O_2_ levels in ZinD enzyme reactions were determined using the Hydrogen Peroxide Quantitative Assay Kit (Sangon Biotech) according to the manufacturer's instructions.

### Structure elucidation of compound 11

Compound **11** was determined to have the molecular formula C_19_H_25_NO_5_ based on the HR-ESI-MS data, with the degree of unsaturation calculated as 8. By comparing the NMR spectra of compound **11** with compound **5**, three additional methylene carbon signals at *δ*_C_ 72.4 ppm (C1"), *δ*_C_ 72.9 ppm (C3"), and *δ*_C_ 63.7 ppm (C4"), as well as a quaternary carbon signal at *δ*_C_ 73.1 ppm (C2"), were clearly observed in compound **11**, which were hypothesized to correspond to the four carbons originated from the Tris component in the Tris-HCl buffer used during the enzymatic reaction. Further ^13^C NMR comparison revealed that the signals of C7 and C9 also showed obvious differences, which shifted from *δ*_C_ 63.3 and *δ*_C_ 56.4 ppm in **5** to *δ*_C_ 98.4 and *δ*_C_ 97.3 ppm in **11**, respectively. Additionally, the proton signals at *δ*_H_ 4.71 (2H) and *δ*_H_ 4.69 (2H) ppm for H-9 and H-7 in **5** were replaced by *δ*_H_ 5.91 (1H) and *δ*_H_ 5.61 (1H) ppm in **11**, suggesting that acetal structures were formed at C9 and C7. HMBC correlation signals from H-7 to C1/C2/C3 and H-9 to C2/C3/C4 further confirmed that the C2 and C3 positions on the benzene ring were replaced by acetal structures. In addition, the HMBC correlations from H-1" to C7/C3"/C4", H3" to C9/C1"/C4", and H-4" to C1"/C3" support the presence of the Tris structure, and the two methylene carbons C1" and C3" in Tris are connected to C7 and C9, respectively, to form acetal. The structure of **11** was further validated *via* ^13^C NMR chemical shift calculation conducted by GIAO ^13^C NMR calculations with STS protocol (24) (Table S17).

### Quantum chemical calculation

DFT calculations were performed by the Gaussian16 software package with the g09defaults keyword (19). Crest (25) was used to search the conformational space of candidate structures on the GFNFF (26) level of theory, followed by optimization on the GFN2-xTB level (27) with a 4 kcal/mol energy window to remove high-energy conformers. The obtained conformer geometries and energies were further optimized on r^2^SCAN-3c level (28) by the ORCA 5.0.2 software package to screen low-energy conformers within 2 kcal/mol (29, 30, 31). Optimization and frequency calculation of each conformer was performed on B3LYP-D3(BJ)/TZVP (IEFPCM, dimethylsulfoxide) level of theory. DFT GIAO ^13^C NMR calculation was calculated on the ωB97x-D/6-31G∗ (IEFPCM, dimethylsulfoxide) level, and the data processing followed the reported STS protocol (24). The calculated shielding tensors of conformers were Boltzmann averaged based on the Gibbs free energy.

## Data availability

All data presented are contained within the article.

## Supporting information

This article contains supporting information.

## Conflict of interest

The authors declare that they have no conflicts of interest with the contents of this article.
