# Supplementary material for: Complete biosynthesis of the fungal alkaloid zinnimidine: Biochemical insights into the isoindolinone core formation
Source: J Biol Chem. 2025 May 31;301(7):110319. doi: 10.1016/j.jbc.2025.110319 (PMC12268544; doi:10.1016/j.jbc.2025.110319)
Supplement: Supplementary Tables and Figures [file mmc1.pdf]

Supporting Information

Complete biosynthesis of the fungal alkaloid zinnimidine: biochemical insights into the isoindolinone core formation

Ling Luo (罗 玲)[a,b,‡], Dan Li (李 丹)[a,b,‡], Bi Long (龙 碧)[a,b,‡], Xiaoling Huang (黄 小玲)[e], Ying Wan (万 影)[a,b], Hongping Long (龙 红萍)[c], Wenxuan Wang (王 文宣)[a,b], Jing Li (李 静)[d], Kangping Xu (徐 康平)[a,b], Guishan Tan (谭 桂山)[a,d] and Xia Yu (于 霞)[a,b,*]

a. Xiangya School of Pharmaceutical Sciences, Central South University, Changsha, Hunan 410013, People's Republic of China;

b. Hunan Key Laboratory of Diagnostic and Therapeutic Drug Research for Chronic Diseases, Central South University, Changsha 410013, People's Republic of China;

c. Center for Medical Research and Innovation, The First Hospital of Hunan University of Chinese Medicine, Changsha, Hunan 410007, People's Republic of China;

d. Xiangya Hospital of Central South University, Central South University, Changsha, Hunan 410008, People's Republic of China;

e. Pharmacy Department, Jiujiang City Key Laboratory of Cell Therapy, JiuJiang NO.1 People's Hospital, Jiujiang, Jingxi 332000, People's Republic of China.

‡ These authors contributed equally to this work.

*Corresponding author: Xia Yu

Email: xyu226@csu.edu.cn.

Table of Contents

Supplementary Tables 1

Table S1: Gene sequences of *zinA-I*. 1

Table S2: Primers used in this study. 11

Table S3: Plasmids used in this study 13

Table S4: The predicted functions of *zin* cluster. 14

Table S5: Protein comparison of *zin* gene cluster to *cic* and *pkf* gene clusters. 15

Table S6: NMR data of compound **1** in CD_3OD. 16

Table S7: NMR data of compound **2** in DMSO-d_6. 17

Table S8: NMR data of compound **3** in acetone-d_6. 18

Table S9: NMR data of compound **4** in CD_3OD. 19

Table S10: NMR data of compound **5** in CD_3OD. 20

Table S11: NMR data of compound **6** in $CDCl_3$. 21

Table S12: NMR data of compound **7** in $CDCl_3$. 22

Table S13: NMR data of compound **8** in $CDCl_3$. 23

Table S14: NMR data of compound **9** in CD_3OD. 24

Table S15: NMR data of compound **10** in $CDCl_3$. 25

Table S16: NMR data of compound **11** in DMSO-d_6. 26

Table S17: Experimental ^{13}C NMR chemical shifts (DMSO-d_6) of **11** and the calculated different configurations of **11** with GIAO ^{13}C NMR calculations with STS protocol 27

Table S18: NMR data of compounds **16** and **2** in DMSO-d_6. 28

Supplementary Figures 29

Figure S1: Maps of plasmids for overexpression of *zinABCDEFHI* in *A. nidulans* A1145. 29

Figure S2: Agarose gel analysis of RNA from *AN-zinABCDEFHI* strain. 30

Figure S3: PCR analysis of *zinA-F*, *zinI* transcription in *AN-zinABCDEFHI* strain 30

Figure S4: SDS-PAGE analysis of purified ZinB, ZinC, ZinE, and ZinD. 31

Figure S5: MS spectrum of compound **3**. 32

Figure S6: MS spectrum of compound **4**. 32

Figure S7: MS spectrum of compound **5**. 32

Figure S8: MS spectrum of compound **2**. 33

Figure S9: MS spectrum of compound **9**. 33

Figure S10: MS spectrum of compound **11**. 33

Figure S11: MS spectrum of compound **12**. 33

Figure S12: MS spectrum of compound **13**. 34

Figure S13: MS spectrum of compound **14**. 34

Figure S14: MS spectrum of compound **15**. 34

Figure S15: MS spectrum of compound **16**. 34

Figure S16: MS spectrum of compound **17**. 34

Figure S17: MS spectrum of compound **18**. 35

Figure S18: MS spectrum of compound **19**. 35

Figure S19: UV spectra of compounds **1-10**........ 36

Figure S20: HPLC analysis of product from heterologous expression of *zinH* in *A. nidulans* A1145. 37

Figure S21: HPLC analysis of *AN-zinABCDEFI* strain cultures after feeding with **1**, **3-6**........ 38

Figure S22: *O*-phthalaldehyde (OPA) reacts with NH_4Cl to form isoindolin-1-one by the non-enzymatic reaction. 38

Figure S23: Proposed mechanism for the transformation of **11**........ 39

Figure S24: H_2O_2 level detection in ZinD-catalyzed reactions........ 39

Figure S25: ^{1}H-NMR spectrum of **1** in CD_3OD (400 MHz)........ 40

Figure S26: ^{13}C-NMR spectrum of **1** in CD_3OD (100 MHz). 40

Figure S27: ^{1}H-NMR spectrum of **2** in DMSO-d_6 (600 MHz). 41

Figure S28: ^{1}H-NMR spectrum of **3** in acetone-d_6 (500 MHz). 41

Figure S29: ^{13}C-NMR spectrum of **3** in acetone-d_6 (125 MHz). 42

Figure S30: ^{1}H-NMR spectrum of **4** in CD_3OD (400 MHz)........ 42

Figure S31: ^{13}C-NMR spectrum of **4** in CD_3OD(100 MHz). 43

Figure S32: ^{1}H-NMR spectrum of **5** in CD_3OD (500 MHz)........ 43

Figure S33: ^{13}C-NMR spectrum of **5** in CD_3OD (100 MHz). 44

Figure S34: ^{1}H-NMR spectrum of **6** in $CDCl_3$ (600 MHz)........ 44

Figure S35: ^{13}C-NMR spectrum of **6** in $CDCl_3$ (150 MHz). 45

Figure S36: ^{1}H-NMR spectrum of **7** in $CDCl_3$ (500 MHz)........ 45

Figure S37: ^{13}C-NMR spectrum of **7** in $CDCl_3$ (100 MHz). 46

Figure S38: ^{1}H-NMR spectrum of **8** in $CDCl_3$ (600 MHz). 46

Figure S39: ^{1}H-NMR spectrum of **9** in CD_3OD (400 MHz). 47

Figure S40: ^{13}C-NMR spectrum of **9** in CD_3OD (100 MHz). 47

Figure S41: HSQC spectrum of **9** in CD_3OD (100 MHz). 48

Figure S42: HMBC spectrum of **9** in CD_3OD (100 MHz). 48

Figure S43: ^{1}H-^{1}H COSY spectrum of **9** in CD_3OD (400 MHz). 49

Figure S44: ^{1}H-NMR spectrum of **10** in $CDCl_3$ (600 MHz). 49

Figure S45: ^{13}C-NMR spectrum of **10** in $CDCl_3$ (150 MHz). 50

Figure S46: ^{1}H-NMR spectrum of **11** in DMSO-d_6 (600 MHz). 50

Figure S47: ^{13}C-NMR spectrum of **11** in DMSO-d_6 (125 MHz). 51

Figure S48: HSQC spectrum of **11** in DMSO-d_6 (125 MHz). 51

Figure S49: HMBC spectrum of **11** in DMSO-d_6 (125 MHz). 52

Figure S50: DEPT135 spectrum of **11** in DMSO-d_6 (125 MHz). 52

Figure S51: ^{1}H-NMR spectrum of **16** in DMSO-d_6 (600 MHz). 53

Supplementary Computation Data 54

Supplementary Tables

Table S1: Gene sequences of *zinA-I*.

Name	Sequence (5′→3′)
zinH	ATGTCACCGAACGCAGACCTCGATGGCATCGCCATCGTCGGGATGGGATGCAGGTT<br>CCCAGGTGCTGATTCCATCGAAGAGTACTGGGACCTCTTGCACGCCGGAAGGTCAAT<br>GGTCACGGAGCCACCCAAAGGACGCTTTCCCTCCCATGAACATGGGAGAAGCACGG<br>ATAAATCCGTCTTCTTCGGCAATTACATAAACGACATTGAAAGTTTCGACAACCGCT<br>TTTTCAAGAAATCAGGCCGCGAGGCCGCTTCTATGGACCCGCAACAAAGACTCCTGC<br>TCGAAGTTGCCTACCAAGCATTGGAATCTGCTGGCTTCTTTGGGCCTAGGGAGCAGG<br>ATTTGGATGTGGGTTGTTTCGTGGGTGTGTGCGCATCGGACTACAATGGTAAGTCGC<br>TTCTACGTACCGCGATGATGTTTTCGGGCTAACGCCGGTGCCTACGATCAGACAATG<br>TCGCCAGTCACCCACCCAACGCCTTTTCGACTCTGGGCACATTGCGTGCGTTTCTTAC<br>CGGTCGTATCAGTCATTTCTTCGGTTTGAGCGGCCCGTCGATTACGTATGACACCGC<br>GTGCTCGTCGTCCGCAGTTGCGATCGATGCTGCATGCAAGGCCATTGCTCACGGAGA<br>CTGCACAAGCGCTCTTGCAGGAGGCGTATCCGTTTTGACTAGCCCAAACTTTTTCCA<br>GAACTTGGCTGCCGCCTCGTTCCTAAGTCCTACTGGTCCTACTGTACGTATAGCCCTA<br>GAGCCAAGATGCACACCTAGACACCTCACATTGAGCTTCACTAACCTATTTGGTACA<br>CGTGTAGAAGTCCTTCGATGCGCGTGCCGATGGCTACTGTCGTGGCGAGGGTGTTGG<br>CCTAGTTGTGTTGAAGAAACTGTCGCAAGCCGTCCAGGATGGCGACCATATCTTCGG<br>TACCATCCTTGCCACGTCCGTCAAGCAAAGTAGCAACAAAGTACCGATCACTGTTCC<br>CTACAGCCCATCACATACCGCACTGTACCGACAGGTGCTGAGCATGGCCGGCGTGG<br>CTGCAGAGGACGTCACGTACCTTGAGGCCCATGGAACCGGCACGCCAATCGGCGAC<br>GTTATCGAGTTCGACGCTCTCAAGGCAACCTTTGCTAGTAAAAGGCGACAACAGCCT<br>CTGTATGTGGGCTCTGTCAAGAGCAACATTGGCCATACGGAGAGTACGTCCGGTGCT<br>GCTAGTCTCATCAAGACGCTGTTGATGATGCAAAAGCGGCAGATCCCCGGGCAGGC<br>AAACTACTCAACGCCCAACACTAAAATCGATCTCTCCGAGGGTCAGTTTGTCATCCC<br>GACACGGACGCAACAGTGGCAAGCGGACACACTCATATCCTGCGTGAACAACTATG<br>GCGCTGCCGGCAGCATTGCGGCCATTGTGCTCAAAGAAGCACCGTCCTTGACAGCTG<br>GCAAAGTGGAAAGCAGGGCCCTAACGAGTTATCCCATCGTGGTGTCCGGAAACAGT<br>AAGAACAGTCTTGCTGCGAACTGCGAGAAGCTAAGAAAGTTTATATCGACGTGCGG<br>GAGGGATGTCCAGATCGCTGACATTGCTTTCAACTTGGCCGACCGGCAGAACCGCA<br>CGTTGCCGCATGTGTTCGCCCGTACCGTCACTAGCTTGTCCGAGCTCGATGGCCACC<br>TCGCAGACGCCGCATCACCCTCTGCAGAACACGAGACCGGAAAGAGACGACCGGTA<br>GTGTTGGCTTTTGGTGGCCAAACGACGCGGTCAATAGGCTTAAGGAAAGAGGTTTA<br>CGATTCTTCCGTCCTGTTGCGTAGATACTTGGACGAATGCGACGGCTTACTGAAGAC<br>ATTCGGATACGAACGAGGGCTCTATCCCGCTATCTTCGACACCAGCTCTCAGGACGA<br>CGTGGTCGGACTCCAGTGTATGCAATTCGCGTTGCATTATGCTTGTGCACAGTCCTG<br>GATTGCTTGTGGCCTTCACATTGATCGCATCATCGGACACTCCTTTGGCCAACTCGTC<br>GCTCTGACCGTAGCGGGCGTTCTGTCGCTTACAGATGGCCTCAGACTAGTCTATGGA<br>CGTGCCGTCCTGATGAGAGACAGGTGGGGCCCAGAACGTGGCTCAATGGTTGCTTT

	GGAGGCTAGCGTCAACGAAACGAGGGCACTGATTGCCTCTGTCCAGCGCCACGACC<br>CCACGGTCCCTGAACTGGAGATAGCATGCTTCAACGGACCAGAAAGCCATGTGCTC<br>GTCGGGTCTGCTGCTGCTGTCGAGACTCTTGTCTCCATTCTCGGGCGGAACAGAACC<br>AAGCACACGGTGCTCAACGTGTCGCATGGTTTCCACTCCCGCTACTGCGACCCCATT<br>CTCCCTGAGCTGGAAGCACTAGCAAGCAGCCTGACTTTCAAGGAGCCAACGATACC<br>CATCGAAACGTGTTCCAGAGGTGGCACGTGGAGCGCAACCACTGCAAGGCTCATTG<br>CTGACCACACGCGCACACCCGTATATTTCGGAGAAGCCGTCAAGCGCATCGAGGCG<br>GATCTCGGACGCGCTACGTGGATAGAGGCTGGTTCAAACTCGTCTATAACGGGCAT<br>GGTCCGCCGCGCTGCCTCGCCAACTGTTGACCACCTATACTGCCCCGTCAACCTTAG<br>CCGGAACGACGCCATGGGACAGATGGCAGATACTTCCGTCAAGCTCTGGAAGAATG<br>GTCACAACGTTCAGTTCTGGCCTTTTCATCGTGCCCAGGCAGCATCATATAGCCGTC<br>TAAGTCTACCTCCATACCAGTTCGAGAGGACGCGCCACTGGCTGGACTTCAACCTTG<br>ACGCTGTGCGGGAACCCAAAAGCGTCGCACCCACGCCACCTGCAGTGTCACCTCCG<br>GTTCCAGAGCCTGTTGTCGAGCCAGATCCCGTATTCCTTGCCTTTTCCGGTTTCCAAG<br>ATGCTGAGCAGAAGGCCACTGTTTTCACCATTGACCCGCGTGCTCCTGAATGGTCAA<br>CGATGCTCGCGGGCCATGCAGTATTGGCTGAACCTCTCTGTCCCGCCCCTCTGTACA<br>TTGAGTTGGCTACCCGTGCGGCAAGAGAGATTGCAAGCCGTCGCGGCCTACAAGGT<br>GCTCAACAAGTTCGCGTAGACGGCCTGGAAATCACCTCCGCATTGGGCGCGAGTTAT<br>GACAAGATGATCAAGCTCACCCTCGACCAAACACATCCAGGAAGCAACGAGTACAC<br>CTTCACGTTCCAGGCGCATAGCCGTGGTGTGTCTGGGACAGGAGCAGACCCTATCTC<br>TACGCATGCTGTCGGCAAGGTCGAGCTTGCCTTGGCCGACGATAAGTCGCACATCGC<br>CGAGTTCAACCGCTTGACTAAGCTCCTTCAATACCACGATTTCGATGAGTACGAGGC<br>GTCTCGCAAGGGGGAGGCGGTCCATGGTGGCCTCGTGTACAAGATATTCTCTCGTGT<br>CGTCCAGTATCATGACTTCTACAAGGGAGTGCGCAAAGTCACGAGCAATGCAGGAG<br>TCATCTCTGCATATGTCTCTCTGCCAGAAGACCAGCCCGCTGCCTTTGAAGGTCTTGT<br>GACGAACCCAGTGGCTATCGACAACTTCCTGCAGGTGCCCGGGATCTACGCAAATT<br>GCTTGGCGCCGTGTCCGTCGGACGAGGTCTTTGTTTGCACCCACGTTGACCGTGTCC<br>AGCTATCACCGGACTTCGCCCTGAGTGGTAGCAATGGCTGGGACGTGCTTGCGATGT<br>CTACACCATCTGGTGAGAGGGAATGCAGCAACGACATATTCGTCAAGGATCATGTC<br>ACGGGACGGCTGGTGTTCGTCATCTTCGGTGCTGCCTTCACGAGGGTCCGCATTTCG<br>TCTTTGTCGAAAGTACTGTTCCGCACAAACGTTCATGACGGTGCGACAGCTGCCGCA<br>GCCACTTCCCGACCCGCACCGGCTGCTGTGAAAGCAGCTGTCAAGGTTGAAAGTCTC<br>ACCAACGCGCCCATTGCCACTCATCATCCCCCAGTCGTATCGCCTGCACTGGCGTCA<br>TTCAACGACCCTGCAACTAAGCAAGCCATGCCATATCTCAATGGAGAAACTGTTCAA<br>ACGGCACCGAAGCCACCTGCACCAAGAGGTCACGTCACCTCACATCAACAAGGACC<br>TAGTGTTGAAGTACGACTTCGGCAAATGCTCAGCAAGATCACGGACGTGCCTGCAG<br>ACCAATTCCAAGGAGATGTGCAGCTTGAGGACCTAGGTATCGACTCTCTAATGGCA<br>ACTGAGATCGTATCCGAAGTATCTGACGTCTTCGGCATCTCAATACCACAAGATCAT<br>CTTCAGGATCTTCTGACGTTTGCGTCGCTGCGGGACTATCTCGACAAGCGAAATGGG<br>AGCACCGCGCCTGCAGTCGAACAGCAGCCTGCTGCTGTTGAACCACCAGTGACCAA<br>AGTGACCAACAGACAAGAACTCGTGTTCACTACCGAGCCAGCCCGCTCGATCCCTGT<br>TAACTCGCACGTAGAGGTTCCCACTAAAAGCGCAGCGGAAAGCACAGGTATGCAGC<br>AGATGATATCGCGTCTGGCACAACTTCTAGGTGAACATCTCGACTGCCCACCGCAAA<br>GCTTCAAGCGCTCTGAGGCTCTCGTCGATTATGGCCTCGACTCTCTGCTGTGCATGG

	AGCTTATGAGCGACATGCAGCAGATCTTCGATGTTTCCATCGATCTGGCGCAGCTCA<br>CCACGGAGAGCACTTACGGCGACCTCGTCGACATTTTCGTGCAGGCTGTAGGTGGCA<br>CGATCACCGCAGACAGCAGTGCCACCCCTAGCACTTCCTCGCACGTCAGTAGCCCGG<br>AGCGTGCGCGGTCGTCTGGTTTTACAACGCCGGCGATCGACGCATCCACTCTTCTCG<br>AGAAGGAAGGCTTGAAAGACACGTATGATGACCCTCTGATACGTTCGCAGGACGTG<br>TTCGAACAGACCAAGACCCAATTCGACCACCTCGCGCTGGAGTACGGGTTCTCTGGA<br>TTTTACGACAAGGTTTACCCTAAGCAATTGCAGCTGGTCGTGGCTTATACTGTCGAA<br>GCCTTTGCTGATCTCGGCATCGACCTGAGGCGGCTGCGTCCTGGTGACGAGATCCCC<br>CTACTCGACGTGCTTCCCAAACACAAACATCTCGCCGTCGCGCTAAACCACATCCTG<br>CGGGAGGGGAAGCTTGCAGACTACGACGGCATGCGCTACGTCCGCTCAGATGTACC<br>AACCGAGGGTGTGCCTTCCAAGGTTCTCTTCAACGCTATCGTAGAGCAATTCCCTCA<br>ACATGCGGACGAACACAAACTCCTTAACATCTGTGGCGCAGAGCTTGGCGCGCTTTT<br>GCGAGGGCAGAAAGATCCATTGGCTTTGCTCTATGGCAACAAAGCGAATCGGACCA<br>TCTTGGAGAACGTGTACAGCACAAGTCCCATGTACATCATCATGTCACGGCTTCTCA<br>CCATGTTCTTGGAGCAAAGCCTGAGCTCGTGTTCACCAGGACCGGATGGCAAGTTCC<br>AAATTCTGGAGCTCGGCGCAGGTACGGGGTCGACGACAAAGTGGGTTGTGGAAGCT<br>CTCGTGCAGCGCGGCATACCTTTCGAATATACATTCACGGACATCTCTTCGTCGCTC<br>GTCTCTGCCGCGAAGCGAAAGTTCGCAAAGTACGGGACCACGATGAAGTACGCCAC<br>TCTGGACATTGAGAAGGAGCCGCCGATCAATCTGCAGGGCCAGTTTGACATCGTGCT<br>GTCGACAAATTGCATCCACGCCACCAAGAATCTATCTACCTCGCTTTCCCATATCAA<br>CAAGCTCCTGAGTCCACACGGGTTTGTCTCGCTGGTAGAATTCACCACCCGCCTTTT<br>CTGGTTTGACCTCGTCTTCGGCCTGCTCGATGGGTGGTGGGCGTTCGAGGATGGCCG<br>TCGCTACGTGCTGGCTAGACCAGAGTTGTGGGCAGAGAACATGCGCTCCAGCGGCT<br>TCGCCCACGTCTCGTGGACAGGCGGCTCGACGCGTGAATCTGAGACTGTGAGGATC<br>ATCACCGGCTTCAAACGACCTGTTGCCGATCCCAGTCTCCCCCAAAGCATTCCTCAA<br>GACCGGAGAGGCGGCATCGAGACCGTCGTCTTTGGCCACGCCGACAACGGGCTGCC<br>GTTGCGGGCGGATATACACTATCCTTCGCCAGCGCAGGCGTCCGCATACGAGACAT<br>GGCCAATCGGTACGCAATAATGTGTCTTCCTTCACCCTCTACACTGCCGCTGGTGGT<br>TCCGCCGCTGACGCCGTGGACTCAGGCCTGATGATACACGGTGGTGGACACGTGAT<br>GCTCTCGCGAAAGGATGTGCGCCCGCGCCAAACGCAACTTCTCCTCAACCACGGCGT<br>GCTTCCTGTGGCCATTGACTACCGGCTTTGCCCCGAAACTACGCTCCTCGAAGGTCC<br>GCTCGTCGACGCGCACCGCGCCTACGCCTGGGCCCGACATGTCCTCCCAACCCTGCA<br>GCTCACCAGCACATCGGTCCGACTCGATGCGTCTCGAGTGGTGGTTATCGGTTGGTC<br>CACCGGAGGCACAGTCGCCATGTCGCTGGCCTGGACGTCCGTACCGCGAGGTCTGC<br>CAGCGCCCGATGCGGTCCTCGTCTTCTACTGCCCGACCGACTACGAAGACGAGTTCT<br>GGACCCGGCCTAATGTCCCAGACCACACGGACACCTACGTCGACGAAACGTTTGAC<br>CTCCTCGAAGGTGTGTATCCCGAGCCCATCACGGCCTACAACGTCCCGTCCAAGATG<br>GTGGCCATGGCTGGCTGGATAGCCCCAAAAGATGCGCGGAGCCGCATCGTCCTGCA<br>CATGAACTGGCACGGCCAGACGCTTCCTGTGCTGTTCCGCGGCCTCCCAAGTGCAAA<br>GAAGGCTGGACGAACCGAGCACACAGCGTGGCATAGTATGGCGCAGCCGTCAAATG<br>ACGAGGTCGTCCAGGGAAGTCCGTACGCTCAGATCGTCAGAGGCAACTACAAGTCA<br>CCAACGCACGTCGTCTTCGGCACAAAGGACGACCTCATCCCGTGGCAGCAGGCCAA<br>GCGGACGGTGGATGCGCTACGCGCGGCGGGAATCGAGTCTGGGCTGACTCTAGTGA<br>AAGACCAGCCGCACCTGTTCGACTTGTACGGAGATCCGACAGGCAAGAAGTGGGAG

	GCGGTGCTCGAGGGGTATGCTTTCCTCTTCAAGAGGATCGGAAGGGAGAGCGCGTAG
zinA	ATGATTTTGAGAATTCTATACACTGTGGTCGTTGCGACCATCATATTCGCACTGGTCAAATCGTTCCAATCGTACACATTCCTCAGGCAAATCCCCGGGCCGAAAGCAGCAGCCCTTACGAACCTGGTCCGCCGCTCATGGGCAAAATCTGGTCGCATACATGAAATTCATCTCCGTTTGCACCGAGACTATGGTTCCGTGGTCCGCGTGGGACCAAATGCTGTGCTGGTCTCGCAGCCGCGCGCCATCGACAGCATCTTTGGCTTCAAGTCGCGACCGAAGAAGGTAGAGTACCCATCATCTTGTTTTTCAGCGAAGCTGAAATATCCATCCGACATAACAAGATCCTCGCTGATGAAGTCGCCTAGTCAGAGTTCTACAACGCGCTCATGCCGCGTATGAAAGGCGGCGCTATCCCCGACGTGTTCGCGACGCTCGACGAGGATGTGCACCGCCAGATGCGGCGGCCTGTCGCGAACCTCTACGGCATCGGCAACCTGGTCTCGTTCGAGCCGCTCATCACGTCCACGCTGCGGCACTTTTTCACGCGCTTGGATGAGCGCTTCACAGACCGCGACACCCCGTTCGATATGTATGATTGGCTACAATTCTTCACATTCGATGTCATTGGCGAGGTGACGTTTTCCCGACGCCTCGGGTTTCTGGATGAAGGAAGGGATATCGAGGGTGTGATTGACGCGAACTGGAAGTATTTCAAGGCCGCGGCACCGGTGAGTGCAGGATTTTCCGTGCCCCATGAAAACTATTGGCGATGATGATGACGCTGTGTGGTGCATGAGCTGATGAAGGGTCGCAGAATACCCAGATGCCGTGGCTGGATTACCTGTGGAAGGATAACCCCCTGCTGCCCGCGGGATCAAAAAAGAATCTCGTGGCGGAGTTTTCTTTTGCGCGGATCCATGAGCGCATGAGCTTGACACCCGCGCAGAGGGAGGACATCAATCAGCGTGACTTTCTGTCCTGCTTTATCAGGGAGAAGGACAAGGACAGTACTTTACCTGCTAGGTAGGCATTTCCAAACAGTCCCATCCTAGCGACATACGTGCACCTCTGACATGGAGATAGCGCCGTCCCCACCTGGACCAACTCAAACATCCAAGCCGGGGTCGACACAACCAGCATCCTCGAAAGCGCCCTCTTCTACCACCTCCTCAAGAACCCGACTACCCTCGCGACTCTGCGCGCCGAGATCGATGATGCTGCAAAGACGGGCCGCATCTCCGAGTTCGTCTCATGGAAGGAGTCGCAGACGCTGCCATATCTTGAGGCTTGCATTCATGAAGCATCGCGGCTGCATCCTCCCATTTGCTTCCCAATCGAACGCATAGTGGGGAAGGAAGGGCTGCACATTGACGGGTTTGTGATTCCACCGGGAACAAGGATATCAATGGTATGTGCTTTCGATGGATTTCGCAGAGAAATACGGAGGATGCTAACGGAGTGGTGTTGATAGAGCCCGTGGACGGTTCATCGTCAAAAGTGGTTGTACGGTGAAGACGCAGACATGTGGCGACCAGAACGGTGGCTCGTGGGCGAGGAGCAAAAACGGGCAATGTATGGCTCATTGCTTACGGTACGTGTCCACCCCCTGTTTGGCCACCCCCTAACGTGCCGCATGTGGCTAACGGTGCACCTACCAGTTTGGCGCGGGCCATCGATCTTGCCTGGGGAAGAACCTGTCGTACTTTCAGATTTTCAAGCTGGTGCCCTCGTTGCTGCAAAAATACGATGTAAGTTGTGTTGTTTTTATTCCCCAGTGCGTTCAGTGCGTAGTTTTGAGCAAGAGGCTGATGAAGGTATAGCTTGAGCTGGTGCGGCCGGACGAGGAGTGGAAGATCGAGAACCATTGGTTCGCGCAGCCGTCGGGGTTTGAGGTGAAGATTTCGCGAAGGAAGTAA
zinB	ATGGAACGCCTTCCTCACACCTTGAAGGACGCCGACGAGCTCCTGGAACTTTCCGCCTTGCTGCAAGAGGCAGCGCTGACGATCAAGAATGAATGGGCCAAAGAAGACTTCTCGTACCAAGATGACAGCGAAGACACAGCGCGCATCCTGCCTAGCCACAAGCTTTGGGACGCGGAGAGAACAGTCGAAGCAGTCACGGGATCTCTAATTGAACTCGTAGCAGAGCCTCATCAGCGCATCCAGCAAATTTTGGCAGAGTTCATGGAATCCCGCGCGCTATTCATCGCCGCCGAGCGCAAGATCCCTGACCTTCTCAATGAGGCGGGTCCTGATGGGTTGGATATTGAGTCGATAGCCGAGAAGACCGGGATTGAGCGAAGGAAGCTGGGTGAGTTACAACGGGCTAGAGATCGTGTTGTATGTGCTAAATTTCTTCAGCTCGCATCTTGAGG

	ACCTTATGTTCAATTCACATCTTCCGCGAGGTTGCAGAAAATCGGTTCGCCAACAAC<br>CGGATCTCTGAAGCTCTGGTTAATAATCCAGGCTTGAGAGCTTATGTGCAGCTGTTG<br>TAAGTGGTTGGGAATCTCCGGTCTCGAAGTCTGCTTTCTGACACCCTCTCAAGCGGA<br>CTGCATATCTACTCGTCGTCTGACCACTTCCCTCGTTACCTACTCGGTTCTAAAGGTC<br>ACTCATACAAAGTCGACGAGACAGCTTTTCACCGCGCCATGGGAACGAATAAGCCT<br>CTTTGGGAGTGGATGTCTGAAAAGCTTCCGCCTAGTCAACTTGTCTCCGATGGCCCG<br>GGCTATCCAGGTGTCCCCGATCTGTCCCTCTTCACTGGTTCCACAGACGCGCCAGGA<br>GCGATCATGAATCGACCGGAGCTTGAAAATTTCGCTTTGGCCATGGTAGCCGGAGG<br>AAAGACCTCTGGCGCTGCTCATGCGTTCGATTTTCCCTGGCTCGAGCTCGGGGAGGG<br>CCTTGTGGTCGATGTTGGTGGAGGCGTCGGTACGTAAGCTCCAGGCTTGGCTCCAAA<br>ATTGGCTAATCTCTTGCATAGGAGGCTTCCCGCTTCAGCTGATGAACGTATACCCAA<br>AGCTGAAGTTTGTCGTGCAAGATCGTCCCGAGAATGTCGAACGTGGCGAGCACGAG<br>ATTTACCCCAAGGAAGCACCTGATGCCGTCTCCAGCGGCCGCGTAACGTTTCAAGCG<br>CATGATTTCTTCGAGCCGAACCCAGTGAAGAACGCCGATGTTTACTGGCTGCGTGGC<br>ATCCTGTGGGTAGACCTTGATTTAGTTCCGGTGTGCCATTCTCTAACTGACGTCTTCT<br>AGCCACGATTGGTCCGATGATTACTGTGTCGCAATCCTCAAAGCCATCAAGACCGCC<br>ATGGGCCCGAAGTCGCGCATTCTCATCTGCGACCCCGTCATGAACACGACCTTTGGC<br>TGCGCCGAGATCCCAGCGGCTCCGAGTCCGCTACCGGCGAATTACGGGTACCACGT<br>GCGGTACTGCCACACTCGCGACCTTGCGCTCATGGCGACATTGAACGGGATCGAGC<br>GGACGCCTACCGAGTTCAAAGCTTTGTTGGAGAGGGCGGGACTTCGACTGCGAAAG<br>TTTTGGGAGGTGAGAAGCATGGTGGGTATCACCGAAGCGAGGCTCGATGACGCGAG<br>TGATTAG
zinC	ATGAACGGCACCACAAACGACCAGATGAGCGGCCACGTAGAGGAGGACTACGTAA<br>AGCATCAAGTGCAAAGCCCCACAAACGTGTGGTTATCTTTGCAAAAAGTCATCCCCT<br>TACAGGATCATGATGCCAGATTTTGGTGGTATCATACGGGCTACCATGTGGCTTGTA<br>TGGTTGACGCCTCCGGATATTCCATGGAGAAACAGTACGAAGTGCTGCTTTTCCACC<br>TAAATTGGATCGTGAGTCTTCCTTGTCAATCGACTTCCTGCTCAGAATAATCCCTGAC<br>TATTTGGTCAGTGTCCCCGACTAGGACCGGCTCCTGAGGGCGATGGAAAGTCGAAA<br>TGGCACTCACTGATGGCGCACGACGGTTCGCCTTTGGAATACTCCTGGAAATGGAAT<br>ACATCTGCAGGAAAGCCGGATATACGATACTCATGGGAGCCGTTCAATCCTGGCTC<br>GGGCAAGACCACTGATCCTCGAAACCATGCGCTGTCGCTAGACTACATGCGCGCTAT<br>GAAGCATGTCCTGCCCGGTGTAGACTTCTCCTGGATCACTACGCTCCTGGAAGAGAT<br>CGAGAGCGGGGATCAAAATGCCTCGCACTTCCTCCACGCTGTGGAGTACAGCCAGA<br>CAAAGTCCTTTGGTCTCAAGTCTTATTTCTTGCCGCGAGACTATAAGATCCTGCAAG<br>CCGGCAGCACGACTACCATGGATGAGTGGGACAAAGCCATCCTGAAACTCAACCCG<br>AATAACGTGGGGCGCGATACCTTGATGGAATTCATCTCTAGCAATCCCGAGGGCAA<br>GCTGTTGCAACCGTGGTATGTCTCGCTTTTACGCCTAGTGGGAGCCTGTAAACTGAT<br>GTAGATGAACAGTGTCCTAGCCGTAGACAATGTGAAGCCCGAGAAGTCGCGGCTGA<br>AACTCTACTTCATGACATCGCACACGAGTTTCTCCTCTCTCCGCGAGATCGTCACCCT<br>CGGCGGCACACGGGATGTTCCAGAGTCCAGCCTCCAAGACCTGAAATCTTTCATCTG<br>GACGCTGCTCGGGCTGCCCGACGACTACCCAGAAGACGTTAACGTACCCGCGCACC<br>CACCCGTTGCCAAGACGTGGCTGGATGAAGAGAATCTTGTCGAGTGTTTCGTTTACT<br>TCTTCGACATCGCGCCGCACAATAGCGAGGTCGACATCAAGTTTTACCTACCGACAA<br>GGAGGTATGGCCCCGACGATCGGCAGATTGCGACACGGCTTGTGGAATGGATGAAG

	TCGAGAGGCCGTGGAGCTTGGTGTGATCGGTATCTGCACATGTTGGAAACGCTGGCT GAGCACCGCGGATTGGAGAATGGAAAGGGATTACACAGCTATATCAGCTATCAGAT CGGGAAGGGGTCGGAGCCAGATATCAAGTCTTATCTCACACCAGAGACGTACCATC CGGCACGATACACATTGTCAAAGGAGTAA
zinD	ATGATTGCTTCTCAACTGATGCATGCTCTGCTTGGAGTTGCTTCATTCGTTTCAGTAG CATCAGGTGCTGCCATCGACAATGATCTTATTTTCTCCCGTCAGGATGCTTGGCCAG CTTCTATTGCAGGCGAGGCCCACGACCAGAGCTGGCCTGAATTTGCCAACGTGACGC AACGTTGGTCTACCTACTCAGCTCCTACTTTCAATGAAGTATTCCTACCCAGGAACG AGAGGGAGCTTTCCATGGGGGTAAGCAAAATCATTCTTTCCTGATCACCAAGTGACC AATTTGCTTACACTATCCTTTTCTAGTTGAGGTACCTGTCGAGCAACAACCGCCCCTT TCTTGCTAAATCAGGCGGTCACGGCTACTCGGCTACTCTTCAGACTGTGCAGAACGC CACCATGATCAACATGGCTGGTTTCAACACCATGAGGATGAACCGAGACCAGAGTG TGACCTACGGCTCCGGTGTGCTCTATGGAGACCTTGTTCAGTTCCTGTACGACCATG GACGCGTAGTTAGTAAGTTTTGACTGAAGTGGTAAACTCAATGAAGATAGCTAATG AGTGTAGCTGTTGGCTCCTGTCCTTGCGTCGGATCCGCCGGTGCTGCTCTCGGTGGC GGTCTCGGCCGTTTCGAAGGTCTCTATGGCCTCACGAGTGATAACATCAGCAAGGTT CGATACGCACTATACAATGGCACCATCGTCGAAGCATCTCCGCAAGTCAACAAAGA TCTCTTCTGGGGAATCCGTGGTGCGGGCCAGAACTTCGGTATCGCTTTCGAGATCAC ACTGCAGACACACCCCGCCACCAACAACGGCCAGCATTACAACGGAGACTTTTACT TCGCACGCAAAGACATGAAGAGGATGTTCGATGCTGTCAACAAACTCGCAGCACCA AGAGGCAACCCTGATCTCTTTGACCCACGTCTTAACTTCATTCCGTTCGTTTCATACA ACACTACCACGCAAGATGTCAGTCATCAGGAACCTTATAATGAGGAAAATACTAAC AACAGAACAGGTTGATATTATCCTCAACGTCCAGTACGCAGGACCCACCCGGGACG CCCAAAAGCACACCAACCAATTCAAGTCCTACGCCACACGCTTCGCGGAGGTCGAG ACATCTTGGCCAGGCACATTCAACGCTGCTGTCGGTGGATTGATCAACTTCTCCTGC ACCAAGGGCGTCAACTACGATATGCGCGGTGTCATCACCAAGACGCTCGACACTCA AATCATCAGCGACTTCGCAGATGACCTCATCGAGATGATCCACGCCAACCCGTCTGC AAACGCCAGCATGTTGATGATCGAAACCTTCCCCAACCAGGCCATGAACAAGTTTCC CGACAACTTTTCCGCCTTCCCTCACCGTGGAAGGTTCAACAACATGATCGAACTTGT CATGCTGTACACGGACGACAGAGAAGCTGCCAATGTTGAGGCTTTCAGTGTCAAGT GGCGCAACAGGTGGGCCGAGAAGAGGTACTCTGGATACGACAGGTTGTACGAGTAC CAGAACTACGGTCATGGCGATGAGCCTTTGAGCGCTCTTTACGGCTACGACCAGTGG CGACACGTCAGGCTGACAGAGTTGAAGAACAAGTATGACCCTCGAGGCTTGTTCAA CGGATACCACCCCATCCCTAACACACTCGCCGGCTGGTCTTAG
zinE	ATGTCTCAATTCGATATTATCGGCGAGCAGTACAAGAAACTACCAGTTCTCATCAAC AGCTCAAATGTTGGTGGCAGGACGTACATCGTAACGGGCGGCAACACCGGGCTCGG GCTCGAAACGGCAAGACACCTAGTCAAAGCGGACGCAGCCCAGGTCATCATCTCGA GTCGCAAGATTTCAGCAGGCGAAGCGGCAAAGGCAGACATCGAGCGCACGACTGG ACGCAAGCATGTGGTCAAGGTCTGGCAGCTTGACCTTACGTCGTTCGCATCCGTCAA AGCATTCGCTGCCAAAGCCTCCGAACTAGAGCGAATCGACGCCCTTATCAACAACG CCGGAATCATGACGGACAAGTTCGCTCTCGCTGAAGGCTACGAGACTGCCATGACA GTCAATGCTATCAGCCCGTTCTTGTTAGCCGCGCTGTTGATGCCGAAGTTACAGGAG ACGGCCCAGCAGTTCAGCACTACCCCTCGCATCGTCATGGTTGGCAGCGCGCTGGCC TTCCAAGCGAACAAAGAACTGGAGAAGGGTGGATCGGAGAACATCTTAGCGAACCT

	GAATGACGTTAAGATTGCAGACCGGTGAGTTCTTTACCTCTTGAATGTGCTTCTTCG<br>ATGGAACTACTTCATGCAACCAAACCTCGCAAGAACGAATGGCTAATGTGTGTGTCC<br>AGCTATCCTGTGACAAAACTTGTCCAGCTATACGCCACCCGCGAATGGGCCTCCCGG<br>TACCCTGTCGAGAAGACCAAGGTCATCCTGAACATGACTGCGCCTGGTGTTTGCTCA<br>ACTGGCCTTGCCCACGACACAAGCGCTGTTACTCGTGCCGTGATTGGTACGATTCGG<br>TTCTTCTTCGCACGGACAGCCGAGGAAGGCAGTCGCACTACAGTTCATGGTGTTATC<br>GCTGGCAATGATAGCCACGGCCAATTTCTCTCCGGCTGCAAGATCAAAGAGTAAGC<br>TGCGTGTGCTGACCTCATATGTCGAACTCTACTAACGGAAGCGATAGTTGGTGGGTC<br>CCCGCCTGGGCCAAGAACGACCACGGCCTACGCACACAAAAACAGTTGTGGAAAGA<br>GCTTACTGGAATCCTGGAGGCGACTTCGCAGAATGTGTTATCCAACATCAAGTCTAT<br>TTCCAACTAG
zinF	ATGTCGACTTTTGTGTCACCACGACTTAATCTAGACCACAACTCAGCATCGGGAATA<br>CGTACCTTGCCCGAACTGGTCGAGTTCCATGTCAAGCACAATCCCCAGCACACATTC<br>TGTATCCAGGCCGAGAAGCCTCTGGTCGAGAATGGCAGACATCGCTTGACATATCTA<br>AGCTACGAGGGCCTACAGCAGGCCATCCTGCAATGCCAAGCATGGCTCAGAGACAA<br>CGCTCCAAACGTCCATGTCTCTTACTTGGACGATGATGGAACTGTGAAGAGATGCGC<br>ACCGGTCGCTGTCCTTATGGAAAGTCATGTTGGCCTGGCCGTTTACATCCTCACCTTG<br>ATGGGAATGGGCGTTCCGGTTGTTCTACTGTCGACACGCCTAAGCCCGGTCGCGATT<br>AGCCACCTTATGCAAACAACCAAGGCCCAGCACGTGTTGGTATCTCAGCGTCTTCAG<br>CCTCTAGTTACCGAGGATCTTGCTGTCGGATGCGCCGAACCGGATAAACAGTGCAGT<br>TTTGAAGTCTGCATCGCGACAGGCTATGACACTTTCCTTGGTCAAGTCCCAGTTACA<br>TCGAACGGACTGATAGCATATCCCAATCATTTTATCGCGGAAGACGATCGCCAGATT<br>CTTATTCTCCATTCGTCGGGGACGTCCGGACTTCCAAAACCCATTTACTGTTCCCATA<br>AGTATTTTTTGGGTTCCGCAAATTGCCATAGCTTCTCCTCAGAAACTGAAGCGCATG<br>GCCTCACAGTCTCCACGTCGCCATTCTTCCATGTGAGTCATTGTATAAAAGAGAGCT<br>CGTGTTTAGTCACTCACACAGAAAGGGATTTGGACTTGTCCCAATGTGCATATCCCT<br>AGGTATTGGCAAGACATTGTGTCTCCCTCCTCCATCCTCCATCCCAACAGGCATCTC<br>AGTAGCAGCACTCCTGGAGGATTCTGGTGCCAAGGCCTTGTTGACAGTGCCATCGAT<br>CGTTGAGGAAATCGCGTTTCTCCAGGATGATCGAGGAATTCAGACTCTTCAACAGCT<br>GGACTTCATTGCTTTCGGTGGAGGTATGCCCAAGGACTCTGTTGGGGAGAAGCTTCG<br>GAAGGCAGGGGTGAAGCTGATTAATCATTACGGTGCGACGGAGACTGGACCATTGT<br>CGCCTTTCTTCGTGCCCGCGGATTCCTACGACTGGCACTATGTTAAGCTTCGGCATG<br>ACACCTTTGTGCCATTGGAGGTTCAGCTGGACCCCGTCAGTGCGCAGGAAAATAGAT<br>TTCAGATGAGTCTTCGGCCTTTTGGGTGGAATGAGCGCTTCGAGCTCCAGGATATTC<br>TTGTCAGGAAACCAGGGTCTCATGAGGAAGACATGGAGTTCTCGATTCAAGGTCGA<br>ACCGATGACTTGATATGTCTCGCAACAGGGGAAAAAGTCCGTCCGACAATCTTGGA<br>GTCTCTTTTGCAGCAACATCATGGCGTCAAAGCGGCTACGACATTCGGAGATGGCCG<br>ATTTGAGCTTGGCGTCATCGTGGAGTCAATTGAGCCTCTTGAAGCCCACCATGTTGA<br>GGCATTCAGAGCGTCTATCTGGCCGCTCATCGAAGAAGCCGGCCATACCATGGATG<br>CACATGCGAGGATCTCATCTCCCGCAGCGGTCTTGGTGTTTCCTCCCGGATTGCTGC<br>CCAGATCCGACAAGGGTACCATACTCCGGTTAGAAGTGGCCAAGAGGTTTTCGAAA<br>GAGATCGACGAGGTATATCAAGAGCTTGAAGGAAATGTGACAGCGACTCCGCTCGA<br>CCGATCCTCGCCCGAGACGACGATTAGAAGCCTGATAGAAGACAACGTAAAGCTGA<br>ACTTGACTTCCACTAGTTGGACCAACGAGGACGATCTCTTCGAGCTTGGTATGGACA

	GCTTACAAGCCGTCAAACTACGTCGGTTGCTCATAGCATCATTGAAGGCAACTGGTG<br>CCAATTCTGATGATCTGTTGAAGCCAGACGAGATCTTGGGGGACTTTGTATATCGCA<br>ACGCTTCCGTTGTCAAGCTTGCTGAAGCAATGACTTCCAAGCATACTACGGTAAACA<br>ACGCTCCGGACTCTCGTTGGTTGGAACGGCTGGTTGATCAATACTCTGGAGGGGACA<br>TTGTACAACCTCAGAAAGCAATAGTCGTCTTGACAGGAGCCACAGGCAGCCTCGGA<br>TCGCACTTCCTGAACGTTCTTCTTAACGACCCTTCGGTAGGTCGGATCATATGTCTCA<br>ATAGACGTACCGAAGAGGACGCAGTTGCGAGACAGAGGCAGTCTTTATACTCCCGA<br>GGTATAGCTGTGAGCGATGAACTTTTCTCAAAGGTTGAGGTCCTAGAGACTAGCCCC<br>GCCATTGAGCGTCTGGGTTTGACTGCTACGCAATACGAATGTCTCGCTCGCGATGTT<br>ACTCACATAGTGCACATTGCTTGGCCTATGAGTTTCAGGATGAACCTGTCATCTTTTA<br>ACGCGACGTTCAAGACCCTACAGAACTTGGTCCAATTTGCATGCGATGCGCGCTCTC<br>AGATTCCGAGAAAGAGGCCGAGGTTCATGTTCGTTTCTTCGATCTCAACAGTCGGGA<br>ATTATCCCAGAATCAGAGCGGAACGCATGGTCCCTGAGGTGGCTGTCGACGATCCA<br>TGCTGGACCCTGGACCTGGGTTACGCCAAAGCGAAACTCGTGTGCGAAAAAATCGT<br>TCAGCGCGCAGCAGAAGATCACCCTGGAATTGAGTTTGGGCTAGTGCGTTTCGGTCA<br>AATTGCCGGATCCAGCACCGGGTATTGGAACCCTGATGAGCACCTCGCCGCGCTGGT<br>TGCATCGTCTCAGACTGTCGGGAAGTTTCCTGAGCTACGAGGGGTAAGAGATCAGA<br>CCACTCTTGTGTTTCCGTCAGCTGACTAACGGTGAGTAGACACTGTCGTGGATGCGT<br>GTGGACTCAGGTGCGGCTGCCCTCGCCGAGCTCATGTTCCATCCGTTACCGTTACAT<br>CTTGTGTACCACCTGGAGAATCATGTCCGACAGAGCTGGCAAGACGTTGTGGCAGT<br>ACTGGTCGAGAAGCTTGGTCTTCCTCAGCGCTCCATCGTCCCGATGAATGATTGGTT<br>GAAGCTTGTTGAGTGTGGCCCTAGCGAAGGCAACCCCGCGCAGAGTCTTCTTGACTT<br>CTTCAGTAATGATTTTGTCAAGATGTCCAACGGGTCCATCATGCTAGACACGGCCAA<br>CACTCGAGGTGTATCGCCTACGCTGAGAAAAATGTCGCCAATCTGCGAAGACGATA<br>TCGCTGCTTACATCGAGTATTGGCGAAGCGTAGGCCTATTGAGACGTGAATGGGAC<br>ATGAATATATCAAGGGAGACAGAGACAGTCCGTGGACAGCGAGTGTTGTTGCCATA<br>AGCATAGGATAGTTTGGGGCGGCAAACTAGGGGAGCCGTTCCGCCCGGCCGTGGAG<br>GGAGCCCCACGACCTGCCCCGCCGAACTGTTCGATTAGTAATCCCATCTCGCTCTTC<br>TTCTCAACGGCTTCCACCTTCAACTTTGACTCTACCCACACGAATCCAGTCGAATATC<br>TCACTGGTAGCGAAACTCAGCGTGCTGTAATGGCCCGATGTTCGCCTTGCCAATGGC<br>CGACACGCCATTTGTCTCTTTGGGCAGTGCATCCACGAGCTGTACGCGAACACGTGG<br>CCGTTCGATGCCACCATCCTCGCGGCATATCACGGCATCTCGATCCTGCAATCATAC<br>GAACAATATTAA
zinG	ATGACGCAAGCGGCAAGATTGCTGTCGCCGGACCACCTCGGGAGTTTGGAGTATCC<br>TGGACGCCTCGCCGCTTTGTTGCATTTGAGTGGCCAAGATTGGATGCTAATTCGAGC<br>AGAAGCGAGAAACGGCAAAGCTTTCAACTGGAACGAGGTCGCAGATCTGTTGCCCG<br>GGCGGACTAACAAAGATTGCCGAAAGAGATGGAGTAAGGTACAAGCCGGCATCAA<br>CAAAGGGGCCTGGACGCGAGCTGAAGATGAGAGGCTCCAGAAGGCAGTCGAAAAA<br>CAAGGCGCAAAGTACGTATGATGTAATTTCGCATCCCCAGGTCCTCTAACACTAGGT<br>AGACCTGTTTGCAGAAACTTTACTTGAAGGTCACTATGCTGACATTAACTAGATGGG<br>CACTAGTTTCGAAGAGTGTGCGGACGCGCAATGCTGACCGTGAGTGTGCTTATCATG<br>CTAGTTGCGAGGTATACGGACTAATGCACTATTTCAGAATGTGCCAAGCGGTGGCA<br>GCACGTCCTAGGGCCCGGGTGTAAACACGGCCCTTGGACAATAGAAGAAGATCAAA<br>AACTGTTAGCAGCTATCGAAAAACATGGAAACAATTGGAAACAGATAGGTCTTTCG

	GAATTACATGGTCGGTCCAGCCACGACATCAGAAATCGGTGAGCTACCCTACCCTAC<br>TCACCCAGATGACTCGCTAAAGGCATGCAGGAGTGTTGCATTGACACGCCGCGGCA<br>AATTCCAAGATCGAGTACAAAGTACTCGTAGCAGCTCAAACGACGTCGAGGAAATC<br>ATGCGGCAGGAACGTATGAGCAACACCCAAGACGAGAACGAACTGGGACTTGATG<br>ATGAATATGGAGAACACAGCTTCTCTGGCATGAATGTCGATAGTGAAGTCGAAACG<br>CCACATCCCTCTCTTGGAGAACGGGCCGATAGGGAACTTTCATTTGGGTCGAACACT<br>AGAGAGGACCCCCACGCTATCAACATCGTCGAAACCGGTGAACTTTCGACCATGAA<br>TCCTATGTGGAGATGGCCAGAGTTCACAACTGGTCCTCTGGTCTTTGATGAACACCA<br>ACTATCCACGACATCGGACACCGGCCACTCTTCCAACTACAACTCGATTTCCGACGC<br>ATCCATCTCAACACCGTCATACCTGAATTCTGCTTTCACTAATTCGACTGTTCTGTCT<br>TTGCACGAGATTAGTCCCAGCACTCCGAACAACCGCACGACACATACAGCGCAGGC<br>TTTTCGGAATCATCATGGAACATTCAACGATATTGGAGACCCTCTATCGTTGGCGCT<br>GGACTCATCACAACATGCGTTGTCGCCGACTACCGCGCCGGAAAAGAAGGGCCATG<br>TCACTATCAGTCTCAAGCAAGTGGATGCAGGGGTTGCACAAGATATAACATCCAGT<br>ATTTTGAAGTACAACGACAGTCTCGTAATTAGACTATATGTAGAGTAG
zinI	ATGGACAGCTCGCGAGCCGATGTTGGAGACAAAACAGCGTCCAAAAGTATAGCCTT<br>ACTTCTGGTCATTTTGAGCCTCGCAGGCTACGAGATCTGGAAACGCCAGGTTAGGCG<br>TAGAGTATGCGCGTCGGAGGCATTTCGTCGTCCTGATGCTGACATGTGATAGCAAGA<br>AGACCAATATACAGCGCAAATGGGATGCAAAGCACCACGGAGATGGGCGGCAAAG<br>TGGCCGGAAGGGTTGGATCTGCTTTTCAAGGTTGGACAACATGCAAGAGCGCAGAC<br>TATGCTTCAGTTCTTCTTGGACGTAGTCGAATCCAGCGGTCCTACCCATGAGCAACA<br>ACTGCGTAAGTGTCTAGGTGTAGGATGGAGCCTTTGGCACTGACACTCATAGCAGAC<br>TTCAGTCTTGGACTCCGACCTAAGCATTCCGCCCCATTGATGGGAAGTGGCATGTTT<br>ACCCAAGATGGTGCAGCGTGGAGGCACTCGCGGGCACTTCTACGGCCTCAGTTCAG<br>TTCCAACCGCTATCAAAATTTCGAGGAGATGAAGAAGTCGGTCGAGAGCCTCGTCG<br>ATCAAATTTCTCCCAATAGCATCGTTGATCTGCAACCGCTGTTCTTCCGTTTGACTTT<br>CGATACGACGACCTTCCTCCTTTTTGGAAAGACACTGAGCTCTTTGCAGAGCAGCGA<br>CATTGCGGGTAAAGAGTCGGAGTTTGCAGCGGCTTTCAACTTGGGCCAGGACTACTT<br>ATCACATCGTGGACGTTTGGGAGACCTATACTGGCTTGCAAATACTCCTGAGTTCTG<br>GCGGGCTTGCAAGACTTCGCATCGCTTCGTCGACGACGCAATTCAGGACGCACTGG<br>ATAATGCGGACAAGCCAAAGCTTGAGAAGGCGGAAGAGGAGGACAAGAAACATTA<br>CGTGTTCATTGATGCGCTCATCCAGGAAACGCGGAACAAGAAAGAACTCCGTGATC<br>AGTGTCTGAACGTTCTCCTAGCAGGCAGAGACACGACCGCATGCTGCCTCAGCTGG<br>ACTCTGCGCTTGCTGGCTCGACATCCTCAGGTTCTGGAGAAACTACGTACAGAGATC<br>GATGAGGTAGTAGGCCTTGGAGAACATGCGCCTCAGCCAACTCGAGTGGATCTGAA<br>GAAGATGAGATACCTCGATCTGGTACTGAAAGAAGTCCTTCGTCTTTATCCATCGGT<br>GCCAGTCAATTCCCGCGCAGCACTCAAGACGACAACGCTACCTGTTGGAGGAGGCC<br>CGGATGGGCAGTCTCCAATATTGGTGGGAAAAGGCGAGGCCGTCGGTTATTGCGTA<br>TATGCCATGCATCGCCGCACAGACATCTACGGAGAAGATGCACTCGAATTCCGGCC<br>CGAAAGGTGGGAAGATGGAACGTTGTTGCGCGATGTAGGCTACGGGTATCTACCCT<br>TCAATGGTGGGCCTCGAGTATGCCTAGGCCAAGAGTTCGCTCTGCTAGAAGCAGGA<br>TACACTGTGGCGAGATTGATACAAAGGTTTCCGTTCGTAACTGTGCCGCAAGACGAT<br>CCTGTTGTCGCGACGGGGAAGGAAAAGCAAATCCTGACACTGGTGGTTGCTTCGGG<br>AGACGGATGCCGTGTACACATGCGCTCGTAG

zinD$_{opt}$	ATGATTGCGAGTCAGCTGATGCATGCGCTGCTGGGCGTGGCGAGCTTTGTGAGCGTG<br>GCGAGCGGCGCGGCGATTGATAACGATCTGATTTTTAGCCGCCAAGATGCGTGGCC<br>GGCGAGCATTGCGGGCGAAGCGCATGATCAGAGCTGGCCGGAATTTGCGAACGTGA<br>CGCAGCGCTGGAGCACCTATAGCGCGCCGACCTTTAACGAAGTGTTTCTGCCGCGCA<br>ACGAACGCGAACTGAGCATGGGCCTGCGCTATCTGAGCAGCAACAACCGCCCGTTT<br>CTGGCGAAAAGCGGCGGCCATGGCTATAGCGCGACCCTGCAGACGGTGCAAAACGC<br>GACCATGATTAACATGGCGGGCTTTAACACCATGCGCATGAACCGCGATCAGAGCG<br>TGACCTATGGCAGCGGCGTGCTGTATGGCGATCTGGTGCAGTTTCTGTATGACCATG<br>GCCGTGTTGTGACGGTGGGCAGCTGCCCATGCGTTGGCAGTGCGGGCGCCGCCTTAG<br>GCGGCGGTCTGGGCCGCTTTGAAGGCCTGTATGGCCTGACGAGCGATAACATTAGC<br>AAAGTGCGCTATGCGCTGTATAACGGCACCATTGTGGAAGCGAGCCCGCAAGTGAA<br>CAAAGATCTGTTTTGGGGCATTCGCGGCGCGGGTCAGAACTTTGGCATTGCGTTTGA<br>AATTACCCTGCAGACCCATCCGGCGACCAACAACGGTCAGCATTATAACGGCGATTT<br>TTATTTTGCGCGCAAAGATATGAAACGCATGTTTGATGCGGTGAACAAACTGGCGGC<br>GCCGCGCGGCAACCCGGATCTGTTTGATCCGCGCCTGAACTTTATTCCGTTTGTGAG<br>CTATAACACCACGACCCAAGATGTGGATATTATTCTGAACGTGCAGTATGCGGGCCC<br>GACCCGCGATGCGCAGAAACATACCAATCAGTTTAAAAGCTATGCGACCCGCTTTG<br>CGGAAGTGGAAACGAGCTGGCCGGGCACCTTTAACGCGGCGGTGGGCGGCCTGATT<br>AACTTTAGCTGCACCAAAGGCGTGAACTATGATATGCGCGGCGTGATTACCAAAAC<br>CCTGGATACGCAGATTATTAGCGATTTTGCGGATGATCTGATTGAAATGATTCATGC<br>GAACCCGAGCGCGAACGCGAGCATGCTGATGATTGAAACCTTTCCGAACCAAGCGA<br>TGAACAAATTTCCGGATAACTTTAGCGCGTTTCCGCATCGCGGCCGCTTTAACAACA<br>TGATTGAACTGGTGATGCTGTATACCGATGATCGCGAAGCGGCGAACGTGGAAGCG<br>TTTAGCGTGAAATGGCGCAACCGCTGGGCGGAAAAACGCTATAGCGGCTATGATCG<br>CCTGTATGAATATCAGAACTATGGCCATGGCGATGAACCGCTGAGCGCGCTGTATG<br>GCTATGATCAGTGGCGCCATGTGCGCCTGACCGAACTGAAAAACAAATATGATCCG<br>CGCGGCCTGTTTAACGGCTATCATCCGATTCCGAACACCCTGGCGGGCTGGAGC

Table S2: Primers used in this study.

Name	Sequence (5′→3′)
AN-zinA-F	CCTTCTCTGAACAATAAACCCCACAGAAGGCATTTATGATTTTGAGAATTC TATACACTG
AN-zinA-R	GGTCTCTCCCGTCACCCAAATCAATTCACCGGAGTGCGATCGCGTTTAAAC ATGTCAGTCGATCGAAG
AN-zinB-F	CTGAGCTTCATCCCCAGCATCATTACACCTCAGCAGCGATCGCATGGAAC GCCTTCCTCACA
AN-zinB-R	TTGATATCGAATTCCTGCAGCCCGGGGGATCCTTAATAGATCTGTTTAAAC ACGGAATGCGAACAGATGA
AN-zinC-F	CCTTCTCTGAACAATAAACCCCACAGAAGGCATTTTTAATTAAGCGATCG CATGAACGGCACCACAAACG
AN-zinC-R	TAGGTCGCCAGGTACGACCAGTTCGGAAGATCAGGAGATCTCCCAGTGAT TGATGCGGC
AN-zinD-F	TAACCATTACCCCGCCACATAGACACATCTAAACATTAATTAAGCGATCG CATGATTGCTTCTCAACTGATG
AN-zinD-R	TAGGTCGCCAGGTACGACCAGTTCGGAAGATCAGGGCTAGCGCTGGTTTT GTGGTTTCATG
AN-zinE-F	TAACCATTACCCCGCCACATAGACACATCTAAACAATGTCTCAATTCGAT ATTATCGG
AN-zinE-R	CCGGGGGATCCACTAGTTCTAGAGCGGCCGCCTTAATGCGATCGCAAACT CAGCGTGCTGTAATG
AN-zinF-F	TCTCTGAACAATAAACCCCACAGAAGGCATTTTTAATTAAATGTCGACTTT TGTGTCAC
AN-zinF-R	GGTCTCTCCCGTCACCCAAATCAATTCACCGGAGTGTTTAAACGCTGACCT CATATGTCGAAC
AN-zinH-F1	AGCTTCATCCCCAGCATCATTACACCTCAGCATTAATATGTCACCGAACGC AGAC
AN-zinH-R1	ATTCCCTCTCACCAGATGGTG
AN-zinH-F2	GCTTGCGATGTCTACACCATC
AN-zinH-R2	CCGGGGGATCCACTAGTTCTAGAGCGGCCGCCTTAATTAACCGAGTGATG AGGTAATCGC
AN-zinI-F	CTGAGCTTCATCCCCAGCATCATTACACCTCAGCAGCGATCGCCCATGGA CAGCTCGCGAGCC
AN-zinI-R	TTGATATCGAATTCCTGCAGCCCGGGGGATCCTTAATGTTTAAACGCTAGC TATAGCTTCCCAACACGTTTGCA
glaA-F	CCTGATCTTCCGAACTGGT
glaA-R	TGCTGAGGTGTAATGATGCT
gpdA-F	ACTCCGGTGAATTGATTTGG
gpdA-R	TGTTTAGATGTGTCTATGTGGC
AmyB-F	GATTAAAGGTGCCGAACGA
AmyB-R	AAATGCCTTCTGTGGGGT
zinA-F	ATGATTTTGAGAATTCTATACACTG

zinA-R	TTACTTCCTTCGCGAAAT
zinB-F	ATGGAACGCCTTCCTCAC
zinB-R	CTAATCACTCGCGTCATCGAG
zinC-F	ATGAACGGCACCACAAA
zinC-R	TTACTCCTTTGACAATGTGTATCG
zinD-F	ATGATTGCTTCTCAACTGATGCA
zinD-R	CTAAGACCAGCCGGCGA
zinE-F	ATGTCTCAATTCGATATTATCGG
zinE-R	CTAGTTGGAAATAGACTTGATGT
zinF-F	ATGTCGACTTTTGTGTCA
zinF-R	TTAATATTGTTCGTATGATTGCA
zinH-F1	ATGTCACCGAACGCAGA
zinH-R1	ATAGCCACTGGGTTCGTC
zinH-F2	CGCTGCCTTTGAAGGTCT
zinH-R2	CTACGCGCTCTCCCTTC
zinI-F	CTCGCGAGCCGATGTTG
zinI-R	CTACGAGCGCATGTGTACAC
pCold-zinB-F	GGAATTCCATATGGAACGCCTTCCTCAC
pCold-zinB-R	CGCGGATCCCTAATCACTCGCGTCATCGAG
pCold-zinC-F	GGAATTCCATATGAACGGCACCACAAA
pCold-zinC-R	GCTCTAGATTACTCCTTTGACAATGTGTAT
pCold-zinE-F	GGAATTCCATATGTCTCAATTCGATATTATCG
pCold-zinE-R	GCTCTAGACTAGTTGGAAATAGACTTGA
SC-zinA-F	ATACAATCAACTATCAACTATTAACTATATCGTAATACCATATGATTTTGA GAATTCTATACACT
SC-zinA-R	AAATTTGTCATTTAAATTAGTGATGGTGATGGTGATGCACCTTCCTTCGCG AAATCT
SC-zinF-F	TACAATCAACTATCAACTATTAACTATATCGTAATACCATATGTCGACTTT TGTGTCAC
SC-zinF-R	TGATAATGGAAACTATAAATCGTGAAGGCATGTTTAAACCTAGGTTAGTG GTGGTGGTGGTGGTGATATTGTTCGTATGATTGCAG
SC-zinI-F	ATACAATCAACTATCAACTATTAACTATATCGTAATACCATATGGACAGCT CGCGAGCC
SC-zinI-R	ATACTTGATAATGGAAACTATAAATCGTGAAGGCATGTTTAAACCTAGTG GTGGTGGTGGTGGTGCGAGCGCATGTGTACACGG
SC-zinD-F	TCAACTATCAACTATTAACTATATCGTAATACCATATGATTGCGAGTCAGC TGATGC
SC-zinD-R	TGTCATTTAAATTAGTGATGGTGATGGTGATGCACGTGATAGCTCCAGCCC GCCAG

Table S3: Plasmids used in this study

Name	Description	Aim
p35-01	*zinH* (*nrpks)* gDNA with downstream 0.2 kb, *zinA* (*p450-1)* gDNA with downstream 0.2 kb, and *zinE (short-chain dehydrogenase)* gDNA with downstream 0.2 kb in pYTU	*A. nidulans* A1145 overexpression
p35-02	*zinB* (*O-methyltransferase)* gDNA with downstream 0.2 kb, and *zinC (prenyltransferase)* gDNA with downstream 0.2 kb in pYTR	*A. nidulans* A1145 overexpression
p35-03	*zinD* (*flavin-dependent oxidoreductase)* gDNA with downstream 0.3 kb, *zinF* (*NRPS-like protein)* gDNA with downstream 0.2 kb, and *zinI (p450-2)* gDNA with downstream 0.3 kb in pYTP	*A. nidulans* A1145 overexpression
p35-04	*zinA* (*p450-1)* gDNA with downstream 0.2 kb, and *zinE (short-chain dehydrogenase)* gDNA with downstream 0.2 kb in pYTU	*A. nidulans* A1145 overexpression
pZY20	*zinD* (*flavin-dependent oxidoreductase*) cDNA in pXW55	*S. cerevisiae* overexpression
pZY21	*zinF* (*NRPS-like protein*) cDNA in pXW06	*S. cerevisiae* overexpression
pZY23	*zinA* (*p450-1)* cDNA in pXW55	*S. cerevisiae* overexpression
pZY24	*zinI* (*p450-2*) cDNA in pXW06	*S. cerevisiae* overexpression
pZY25	*zinD*$_{opt}$ (*flavin-dependent oxidoreductase*) in pXW55	*S. cerevisiae* overexpression
pZY26	*zinB* (*O-methyltransferase*) cDNA in pCold	*E. coli* overexpression
pZY27	*zinC* (*prenyltransferase*) cDNA in pCold	*E. coli* overexpression
pZY29	*zinE* (*short-chain dehydrogenase*) cDNA in pCold	*E. coli* overexpression

Table S4: The predicted functions of *zin* cluster.

Gene	Size (gene/protein)	Known homolog from Uniprot (origin)	Identity (%)	Predicted function	E-value
zinA	1916/495	C8V0D4 *Aspergillus nidulans*	54.6	cytochrome P450	0
zinB	1648/480	A0A1U8QH20 *Aspergillus nidulans*	57.6	O-methyltransferase	0
zinC	1441/443	W6QY29 *Penicillium roqueforti*	43.1	prenyltransferase	$8e^{-126}$
zinD	1684/507	G4N285 *Pyricularia oryzae*	31.7	flavin-dependent oxidoreductase	$6e^{-69}$
zinE	1138/331	A0A4P8DJW5 *Cryptosporiopsis* sp.	38.3	short-chain dehydrogenase	$3e^{-67}$
zinF	3701/1199	A0A1U8QW91 *Aspergillus nidulans*	40.6	NRPS-like protein	0
zinG	1456/417	A0A1U8QVN4 *Aspergillus nidulans*	44.2	transcriptional regulator	$7e^{-39}$
zinH	7136/2308	Q5AZ32 *Aspergillus nidulans*	45.2	nrPKS	0
ORF1	1347/448	Q9UV10 *Neurospora crassa*	42.0	Heterokaryon incompatibility protein	0.002
ORF2	1824/607	none	none	none	none
zinI	1607/503	D4AY62 *Trichophyton benhamiae*	58.5	cytochrome P450	0

Table S5: Protein comparison of *zin* gene cluster to *cic* and *pkf* gene clusters.

zin	**Size (aa)**	**Proposed function**	***pkf* (*cic*) analog**	**Coverage/Identity (%)**
ZinA	495	P450	PkfB (CicH)	98/40.7 (100/54.6)
ZinB	480	O-methyltransferase	CicE	100/57.6
ZinE	331	short-chain dehydrogenase	PkfC (AN6450)	71/26.2 (40/23.5)
ZinF	1199	NRPS-like protein	PkfA (CicB)	37/33.5 (90/40.6)
ZinH	2308	nrPKS	PkfA (PkbA)	66/34.2 (99/45.2)

Table S6: NMR data of compound **1** in CD_3OD.

Compound	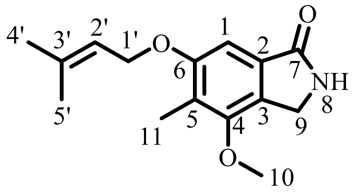 **1**	
position	δ_H, (multi., *J*)	δ_C
1	6.46 (s)	101.7
2	-	132.4
3	-	127.4
4	-	159.8
5	-	124.4
6	-	155.1
7	-	173.7
8	-	-
9	3.92 (s)	45.1
10	3.31 (overlaps)	60.1
11	1.57 (s)	9.7
1’	4.01 (d, *J*=6.6 Hz)	66.6
2’	4.92 (t, *J*=6.6 Hz)	120.9
3’	-	138.9
4’	1.19 (s)	25.9
5’	1.21 (s)	18.3

^{1}H NMR Spectrometer Frequency: 400.13 MHz, Temperature: 294.5 K, ^{13}C NMR Spectrometer Frequency: 100.62 MHz, Temperature: 294.4 K.

Table S7: NMR data of compound **2** in DMSO-d_6.

Compound	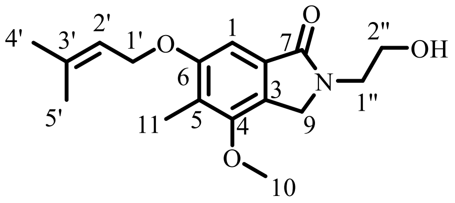 **2**
position	δ_H, (multi., *J*)
1	6.96 (s)
2	-
3	-
4	-
5	-
6	-
7	-
8	-
9	4.62 (s)
10	3.87 (s)
11	2.09 (s)
1’	4.60 (d, *J*=6.4 Hz)
2’	5.44 (t, *J*=6.4 Hz)
3’	-
4’	1.72 (s)
5’	1.75 (s)
1’’	3.55 (t, *J*=5.7 Hz)
2’’	3.62 (m)
2’’-OH	4.81 (t, *J*=5.4 Hz)

^{1}H NMR Spectrometer Frequency: 600.15 MHz, Temperature: 298.0 K.

Table S8: NMR data of compound **3** in acetone-d_6.

Compound	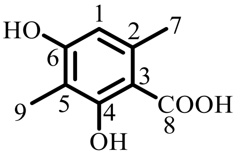<br>**3**	
position	δ_{H}, (multi., J)	δ_{C}
1	6.33 (s)	104.7
2	-	141.1
3	-	111.2
4	-	160.9
5	-	109.3
6	-	164.9
7	2.02 (s)	24.2
8	-	174.9
9	2.48 (s)	8.1

^{1}H NMR Spectrometer Frequency: 500.13 MHz, Temperature: 297.4 K, ^{13}C NMR Spectrometer Frequency: 125.77 MHz, Temperature: 298.3 K.

Table S9: NMR data of compound **4** in CD_3OD.

Compound	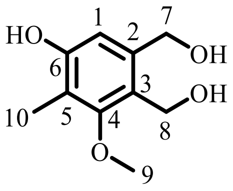<br>**4**	
position	δ_H, (multi., J)	δ_C
1	6.70 (s)	112.1
2	-	140.8
3	-	123.7
4	-	159.7
5	-	117.8
6	-	157.4
7	4.67 (s)	63.3
8	4.65 (s)	62.2
9	3.74 (s)	56.5
10	2.11 (s)	9.33

^{1}H NMR Spectrometer Frequency: 400.13 MHz, Temperature: 295.0 K, ^{13}C NMR Spectrometer Frequency: 100.62 MHz, Temperature: 295.0 K.

Table S10: NMR data of compound **5** in CD_3OD.

Compound	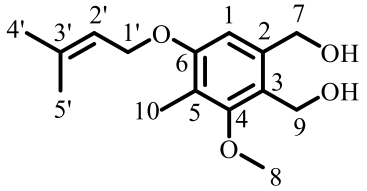 **5**	
position	δ_H, (multi., J)	δ_C
1	6.84 (s)	108.9
2	-	140.9
3	-	124.8
4	-	159.3
5	-	119.7
6	-	158.9
7	4.69 (s)	63.3
8	3.74 (s)	62.3
9	4.71 (s)	56.4
10	2.12 (s)	9.4
1'	4.56 (d, J=6.5 Hz)	66.2
2'	5.48 (t, J=5.5 Hz)	121.4
3'	-	138.4
4'	1.75 (s)	25.9
5'	1.79 (s)	18.2

^{1}H NMR Spectrometer Frequency: 500.13 MHz, Temperature: 294.6 K, ^{13}C NMR Spectrometer Frequency: 100.61 MHz, Temperature: 295.5 K.

Table S11: NMR data of compound **6** in $CDCl_3$.

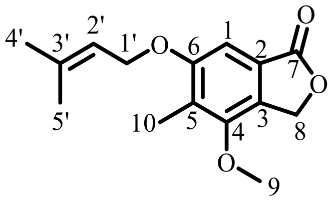

Compound	**6**	
position	δ_H, (multi., J)	δ_C
1	7.08 (s)	102.0
2	-	128.2
3	-	124.9
4	-	159.3
5	-	125.9
6	-	153.0
7	-	171.5
8	5.37 (s)	68.4
9	3.88 (s)	59.4
10	2.21 (s)	9.9
1’	4.57 (d, J=7.2 Hz)	65.9
2’	5.48 (t, J=6.6 Hz)	119.4
3’	-	138.4
4’	1.75 (s)	25.9
5’	1.80 (s)	18.4

^{1}H NMR Spectrometer Frequency: 600.15 MHz, Temperature: 298.0 K, ^{13}C NMR Spectrometer Frequency: 150.92 MHz, Temperature: 298.0 K.

Table S12: NMR data of compound **7** in $CDCl_3$.

Compound	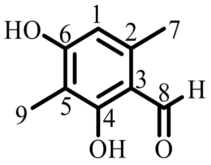 **7**	
position	δ_H, (multi., *J*)	δ_C
1	6.21 (s)	110.1
2	-	141.5
3	-	113.4
4	-	161.4
5	-	110.1
6	-	164.3
7	2.08 (s)	18.1
8	10.07 (s)	193.2
9	2.49 (s)	7.0
4-OH	12.66 (s)	-

^{1}H NMR Spectrometer Frequency: 500.13 MHz, Temperature: 295.6 K, ^{13}C NMR Spectrometer Frequency: 100.62 MHz, Temperature: 294.4 K.

Table S13: NMR data of compound **8** in $CDCl_3$.

Compound	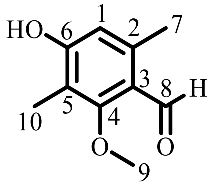 **8**
position	δ_H, (multi., *J*)
1	6.47 (s)
2	-
3	-
4	-
5	-
6	-
7	2.53 (s)
8	10.37 (s)
9	3.82 (s)
10	2.17 (s)

^{1}H NMR Spectrometer Frequency: 600.15 MHz, Temperature: 298.0 K.

Table S14: NMR data of compound **9** in CD_3OD.

Compound	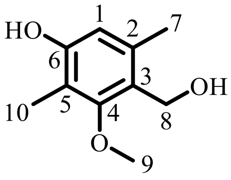 **9**			
position	δ_H, (multi., *J*)	δ_C	^1H-^1H COSY	HMBC
1	6.44 (s)	113.7	2.31	C2, C5, C6, C7
2	-	123.9		
3	-	137.9		
4	-	159.8		
5	-	115.8		
6	-	157.1		
7	2.31 (s)	19.0	6.44	C1, C2, C3, C4
8	4.61 (s)	56.9		C2, C3, C4
9	3.73 (s)	62.2		C4
10	2.08 (s)	9.1		C4, C5, C6

^1H NMR Spectrometer Frequency: 400.13 MHz, Temperature: 297.4 K, ^{13}C NMR Spectrometer Frequency: 100.62 MHz, Temperature: 297.6 K.

Table S15: NMR data of compound **10** in $CDCl_3$.

Compound	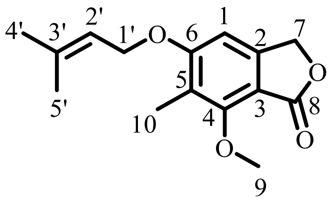 **10**	
position	δ_H, (multi., *J*)	δ_C
1	6.61 (s)	99.6
2	-	109.6
3	-	148.2
4	-	163.7
5	-	120.9
6	-	157.9
7	5.17 (s)	68.9
8	-	169.2
9	4.02 (s)	62.2
10	2.14 (s)	8.8
1’	4.59 (d, *J*=6.6 Hz)	65.9
2’	5.48 (t, *J*=5.8 Hz)	119.1
3’	-	138.7
4’	1.75 (s)	25.9
5’	1.81 (s)	18.4

^{1}H NMR Spectrometer Frequency: 600.15 MHz, Temperature: 298.0 K, ^{13}C NMR Spectrometer Frequency: 150.92 MHz, Temperature: 298.0 K.

Table S16: NMR data of compound **11** in DMSO-d_6.

Compound	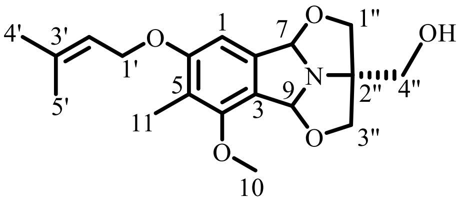 **11**		
position	δ_H, (multi., *J*)	δ_C	HMBC
1	6.74 (s)	102.0	C2, C3, C4, C5, C6, C7
2	-	139.0	-
3	-	120.7	-
4	-	154.2	-
5	-	118.5	-
6	-	159.2	-
7	5.61 (s)	98.4	C1, C2, C3, C6, C9
9	5.91 (s)	97.3	C2, C3, C4, C7, C3’’
10	3.86 (s)	60.0	C4
11	2.01 (s)	9.1	C1, C4, C5, C6
1’	4.53 (d, *J*=6.7 Hz)	65.2	C2’, C3’, C6
2’	5.44 (t, *J*=6 .7 Hz)	120.0	C4’, C5’
3’	-	137.2	-
4’	1.70 (s)	25.2	C2’, C3’, C5’
5’	1.75 (s)	18.1	C2’, C3’, C4’
1’’	3.97 (d, *J*=8.5 Hz)<br>3.62 (d, *J*=8.4 Hz)	72.4	C7, C3’’, C4’’
2’’	-	73.1	-
3’’	3.97 (d, *J*=8.5 Hz)<br>3.76 (d, *J*=8.6 Hz)	72.9	C9, C1’’, C4’’
4’’	3.51 (d, *J*=3.6 Hz)	63.7	C1’’, C3’’

^{1}H NMR Spectrometer Frequency: 600.05 MHz, Temperature: 298.0 K, ^{13}C NMR Spectrometer Frequency: 125.77 MHz, Temperature: 294.5 K.

Table S17: Experimental ^{13}C NMR chemical shifts (DMSO-d_6) of **11** and the calculated different configurations of **11** with GIAO ^{13}C NMR calculations with STS protocol.

Compound		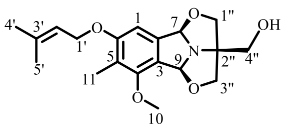 7*R*9*S*2"*R*-**11**		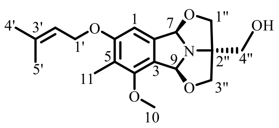 7*R*9*S*2"*S*-**11**		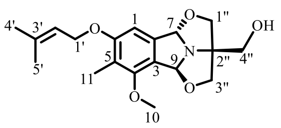 7S9*S*2"R-**11**		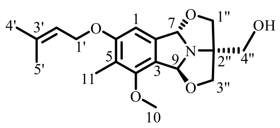 7S9*S*2"*S*-**11**	
position	Exptl.	Calcd.	Deviation	Calcd.	Deviation	Calcd.	Deviation	Calcd.	Deviation
1	102.0	102.84	0.84	102.57	0.57	107.03	5.03	100.32	1.68
2	139.0	145.29	6.29	140.40	1.40	142.26	3.26	141.49	2.49
3	120.7	123.01	2.31	117.76	2.94	116.54	4.16	116.34	4.36
4	154.2	154.81	0.61	154.84	0.64	153.45	0.75	157.22	3.02
5	118.5	117.38	1.12	117.95	0.55	117.32	1.18	115.21	3.29
6	159.2	157.23	1.97	157.89	1.31	155.07	4.13	156.51	2.69
7	98.4	86.74	11.66	97.14	1.26	88.99	9.41	97.76	0.64
9	97.3	86.54	10.76	96.94	0.36	98.02	0.72	89.29	8.01
10	60.0	58.73	1.27	58.22	1.78	58.61	1.39	58.88	1.12
11	9.1	13.44	4.34	12.64	3.54	11.47	2.37	11.73	2.63
1’	65.2	65.33	0.13	64.50	0.70	63.87	1.33	64.10	1.10
2’	120.0	120.01	0.01	120.28	0.28	120.27	0.27	120.38	0.38
3’	137.2	142.22	5.02	143.01	5.81	142.94	5.74	142.99	5.79
4’	25.2	27.75	2.55	26.97	1.77	25.70	0.50	26.20	1.00
5’	18.1	20.12	2.02	19.58	1.48	18.19	0.09	18.86	0.76
1’’	72.4	71.82	0.58	69.81	2.59	75.31	2.91	81.58	9.18
2’’	73.1	72.28	0.62	69.90	3.00	80.19	7.29	75.82	2.92
3’’	72.9	75.17	2.07	75.36	2.26	69.45	3.65	69.65	3.45
4’’	63.7	65.49	1.79	60.42	3.28	61.53	2.17	61.84	1.86
		MAE	2.94	MAE	1.87	MAE	2.97	MAE	2.97
		RMS	4.40	RMS	2.32	RMS	3.86	RMS	3.79
		P_{mean}	0	P_{mean}	9.56%	P_{mean}	0.41%	P_{mean}	0.44%
		P_{rel}	0	P_{rel}	100.00%	P_{rel}	0	P_{rel}	0

Table S18: NMR data of compounds **16** and **2** in DMSO-d_6.

Compound	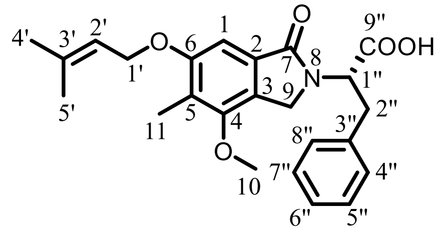 **16**	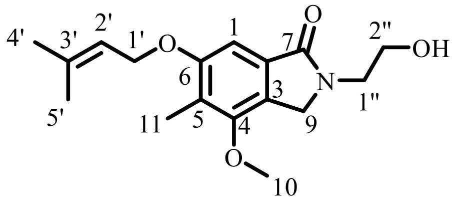 **2**
position	δ_H, (multi., *J*)	δ_H, (multi., *J*)
1	6.88 (s)	6.96 (s)
2	-	-
3	-	-
4	-	-
5	-	-
6	-	-
7	-	-
8	-	-
9	4.11 (s)	4.62 (s)
10	3.84 (s)	3.87 (s)
11	2.06 (s)	2.09 (s)
1’	4.56 (d, *J*=6.8 Hz)	4.60 (d, *J*=6.4 Hz)
2’	5.41 (t, *J*=6.5 Hz)	5.44 (t, *J*=6.4 Hz)
3’	-	-
4’	1.69 (s)	1.72 (s)
5’	1.73 (s)	1.75 (s)
1’’	5.01 (dd, *J*=11.6, 4.5 Hz)	3.55 (t, *J*=5.7 Hz)
2’’	4.58 (d, *J*=18.0 Hz)<br>4.48 (d, *J*=16.7 Hz)	3.62 (m)
3’’	-	-
4’’/8’’	7.24 (d, *J*=6.7 Hz)	-
5’’/7’’	7.21 (t, *J*=7.5 Hz)	-
6’’	7.12 (t, *J*=7.2 Hz)	-
9’’	-	-

^{1}H NMR Spectrometer Frequency: 600.05 MHz, Temperature: 298.0 K.

Supplementary Figures

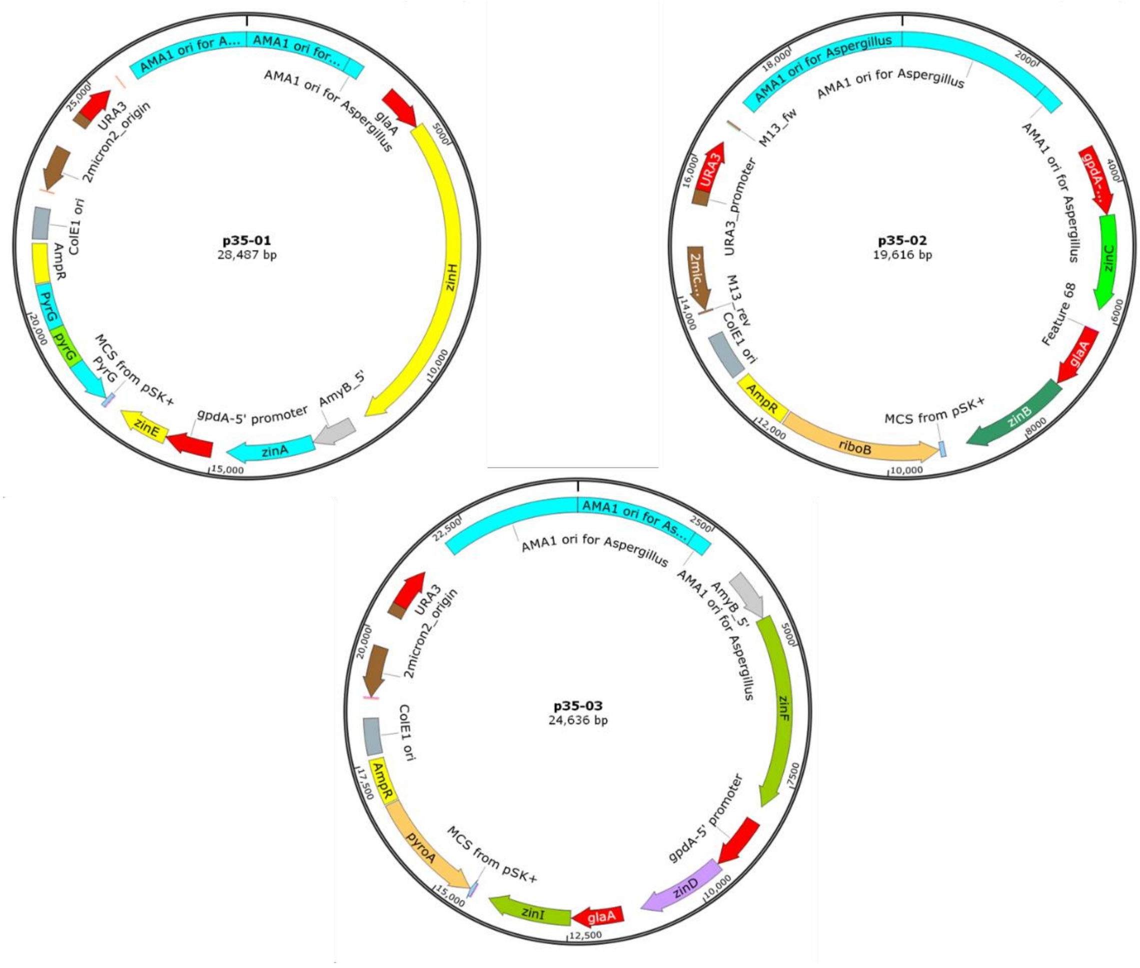

Figure S1: Maps of plasmids for overexpression of *zinABCDEFHI* in *A. nidulans* A1145.

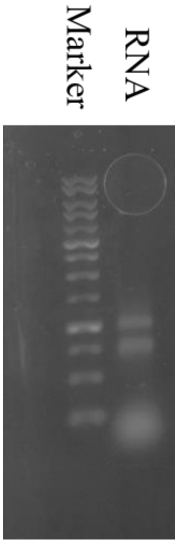

Figure S2: Agarose gel analysis of RNA from *AN-zinABCDEFHI* strain (*zinABCDEFHI* overexpression).

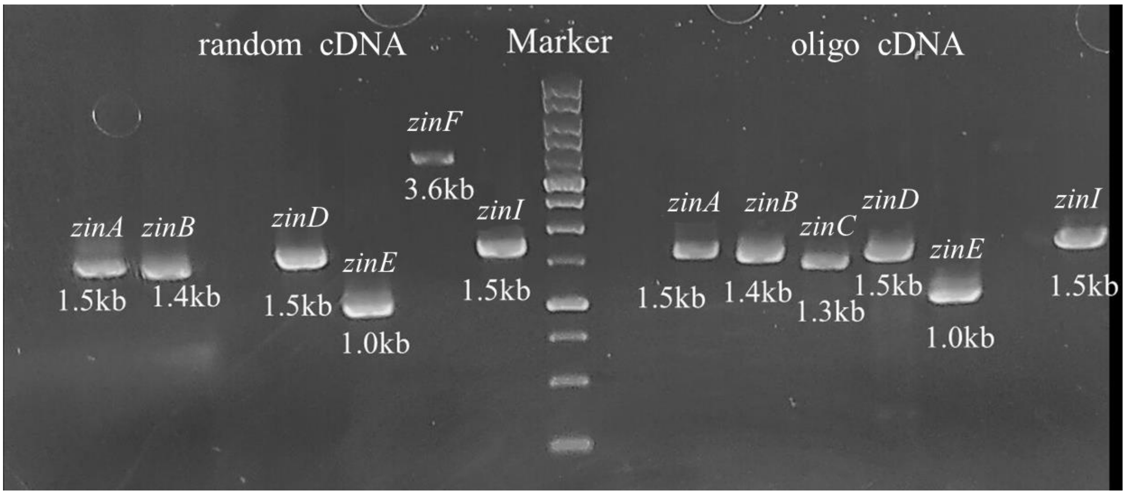

Figure S3: PCR analysis of *zinA-F*, *zinI* transcription in *AN-zinABCDEFHI* strain.

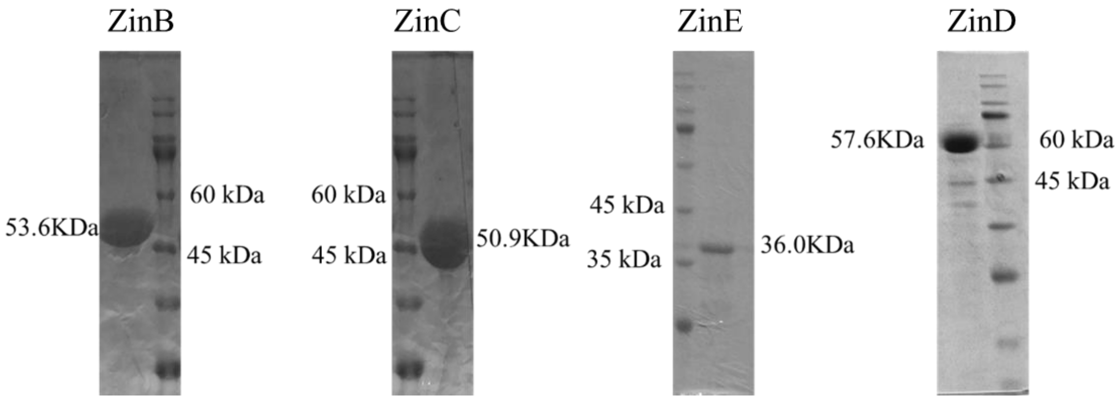

Figure S4: SDS-PAGE analysis of purified ZinB, ZinC, ZinE, and ZinD.

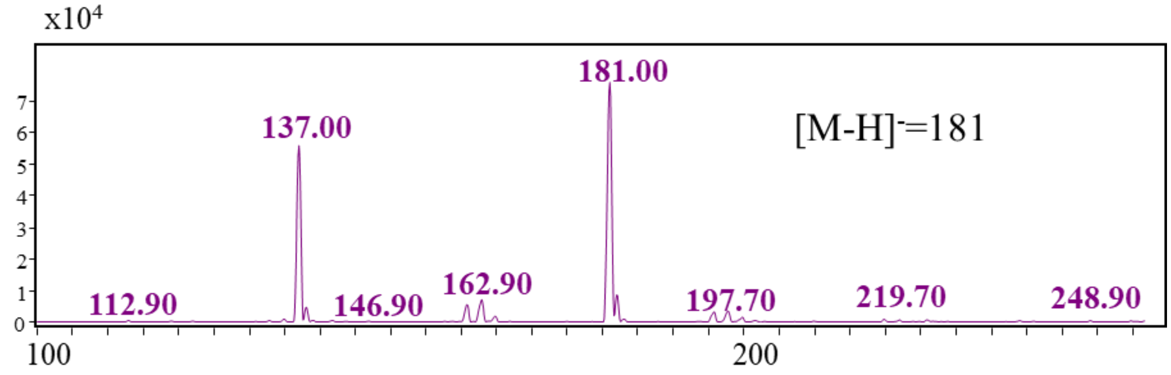

Figure S5: MS spectrum of compound **3** ($C_9H_9O_4^-$, calculated *m/z*=181.1 $[M-H]^-$).

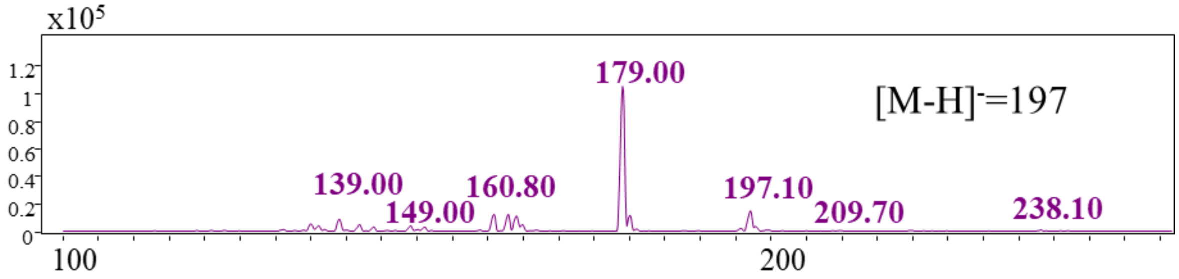

Figure S6: MS spectrum of compound **4** ($C_{10}H_{13}O_4^-$, calculated *m/z*=197.1 $[M-H]^-$).

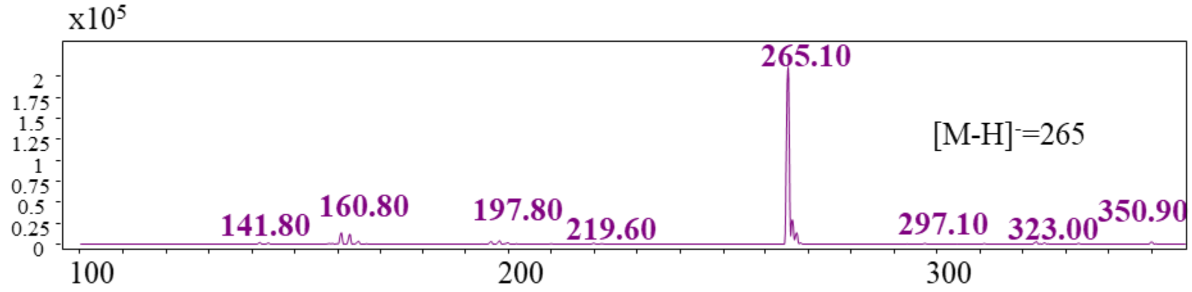

Figure S7: MS spectrum of compound **5** ($C_{15}H_{21}O_4^-$, calculated *m/z*=265.1 $[M-H]^-$).

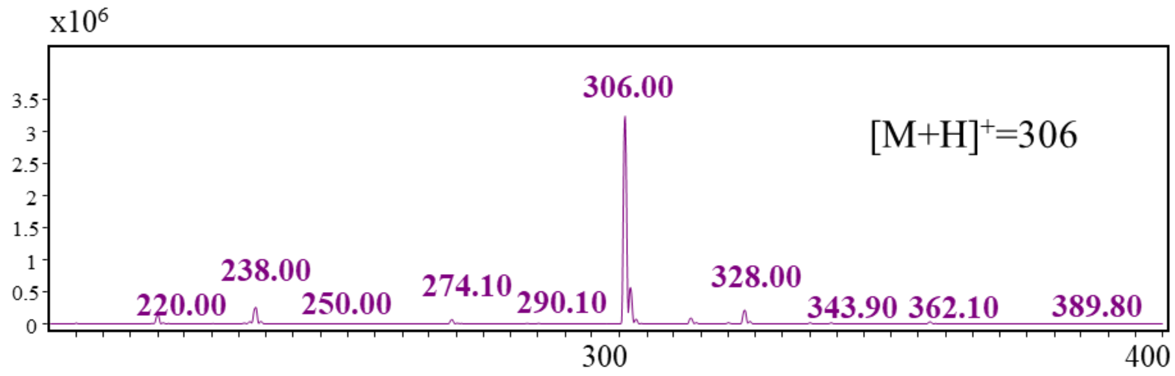

Figure S8: MS spectrum of compound **2** ($C_{17}H_{24}NO_4^+$, calculated *m/z*=306.2 $[M+H]^+$).

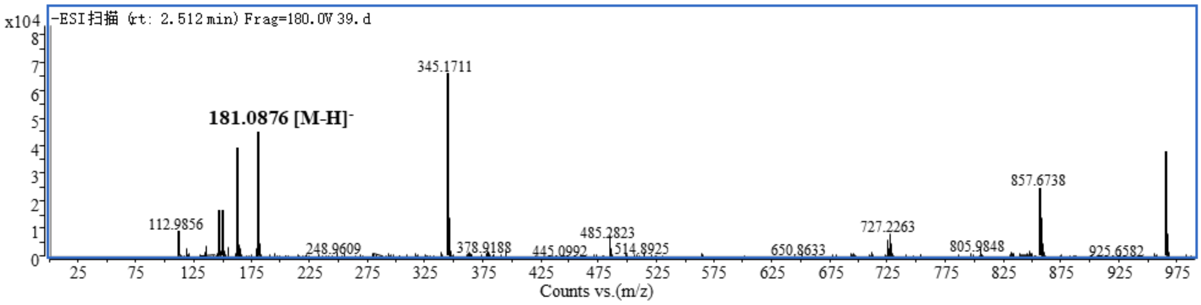

Figure S9: MS spectrum of compound **9** ($C_{10}H_{13}O_3^-$, calculated *m/z*=181.0807 $[M-H]^-$).

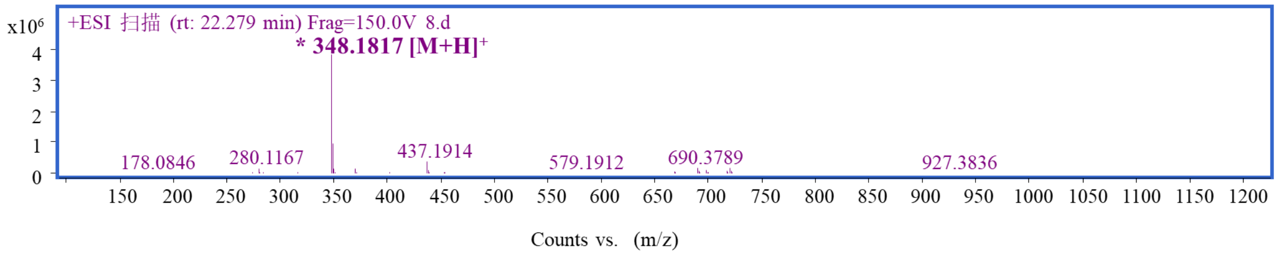

Figure S10: MS spectrum of compound **11** ($C_{19}H_{26}NO_5^+$, calculated *m/z*=348.1805 $[M+H]^+$).

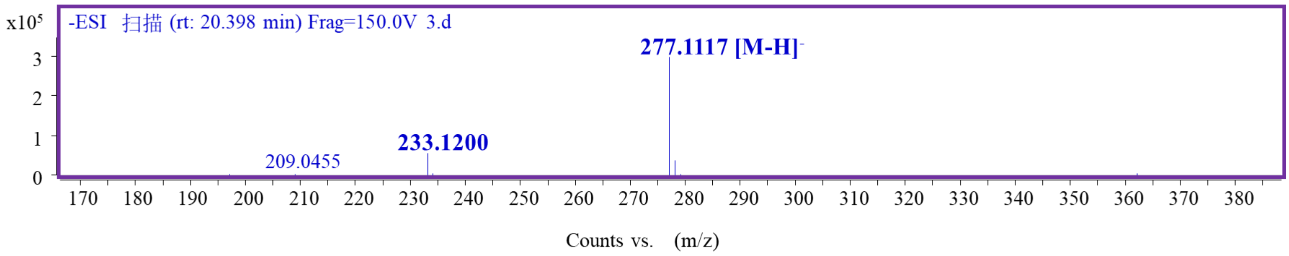

Figure S11: MS spectrum of compound **12** ($C_{15}H_{17}O_5^-$, calculated *m/z*=277.1081 $[M-H]^-$).

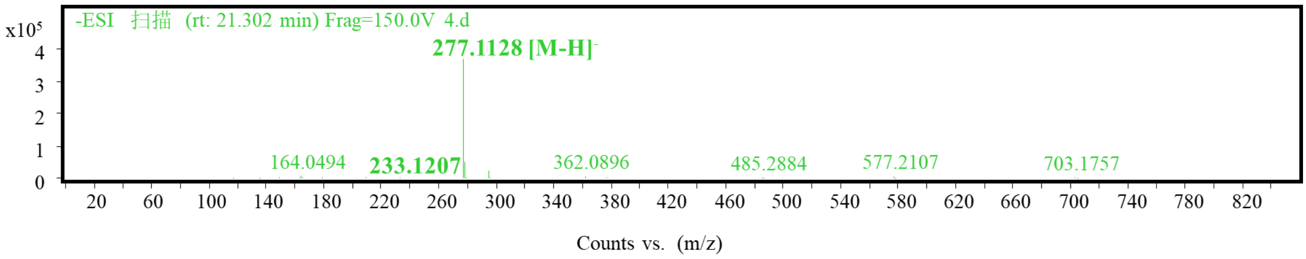

Figure S12: MS spectrum of compound **13** ($C_{15}H_{17}O_5^-$, calculated *m/z*=277.1081 [M-H]$^-$).

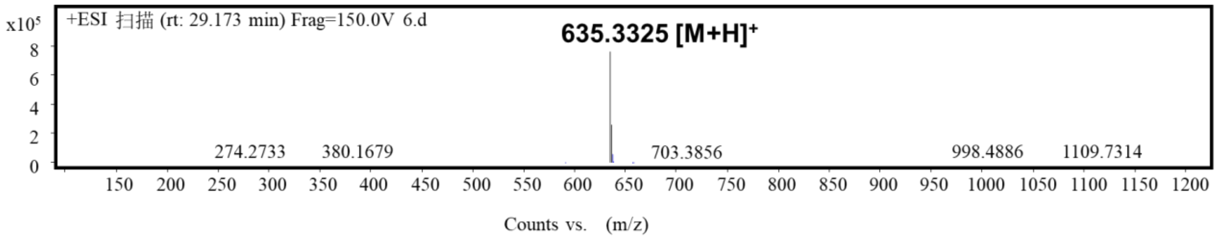

Figure S13: MS spectrum of compound **14** ($C_{36}H_{47}N_2O_8^+$, calculated *m/z*=635.3327 [M+H]$^+$).

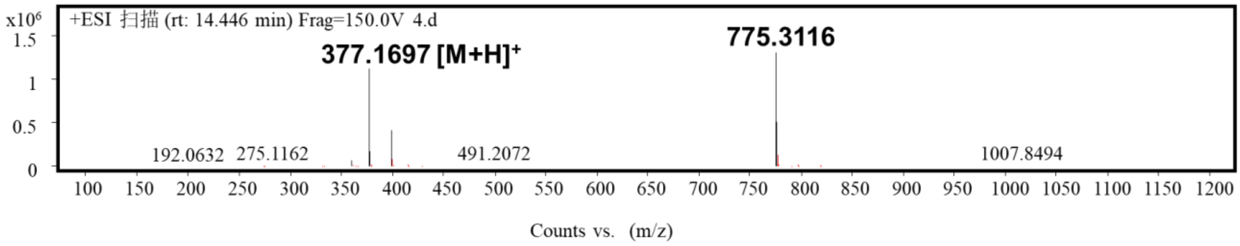

Figure S14: MS spectrum of compound **15** ($C_{19}H_{25}N_2O_6^+$, calculated *m/z*=377.1707 [M+H]$^+$).

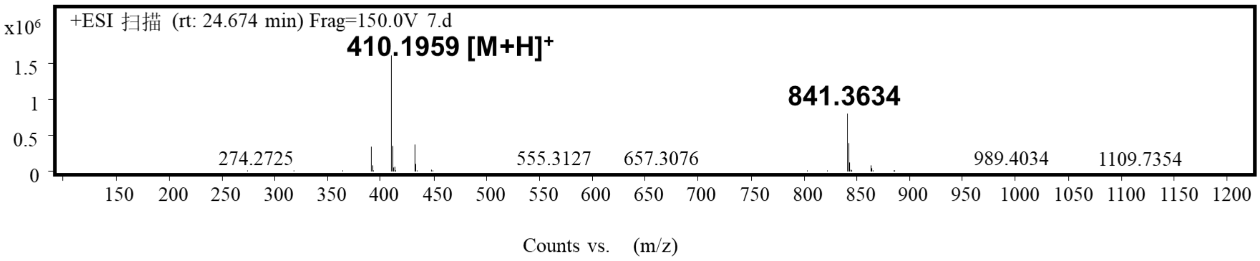

Figure S15: MS spectrum of compound **16** ($C_{24}H_{28}NO_5^+$, calculated *m/z*=410.1962 [M+H]$^+$).

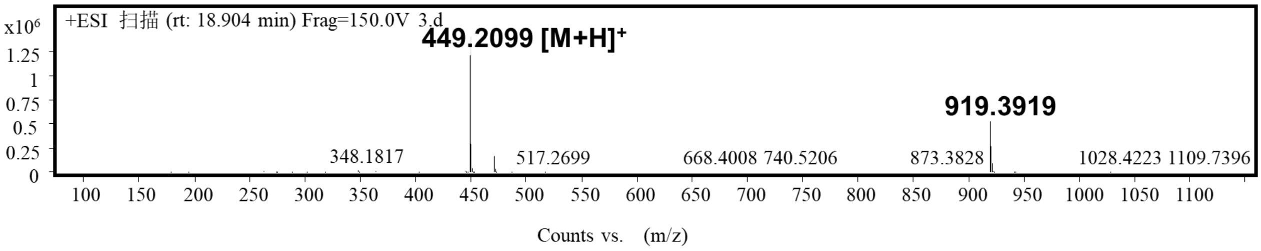

Figure S16: MS spectrum of compound **17** ($C_{26}H_{29}N_2O_5^+$, calculated *m/z*=449.2071 [M+H]$^+$).

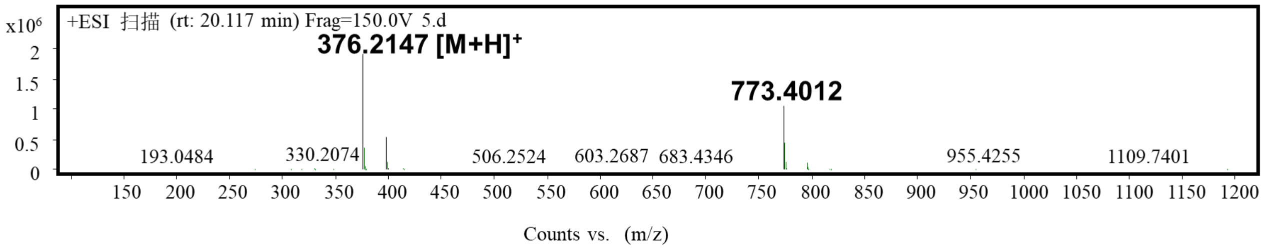

Figure S17: MS spectrum of compound **18** ($C_{21}H_{30}NO_5^+$, calculated *m/z*=376.2118 [M+H]$^+$).

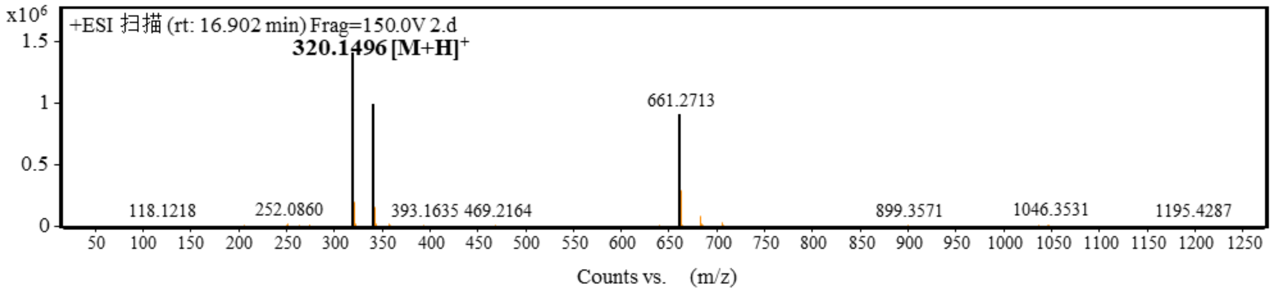

Figure S18: MS spectrum of compound **19** ($C_{17}H_{22}NO_5^+$, calculated *m/z*=320.1492 [M+H]$^+$).

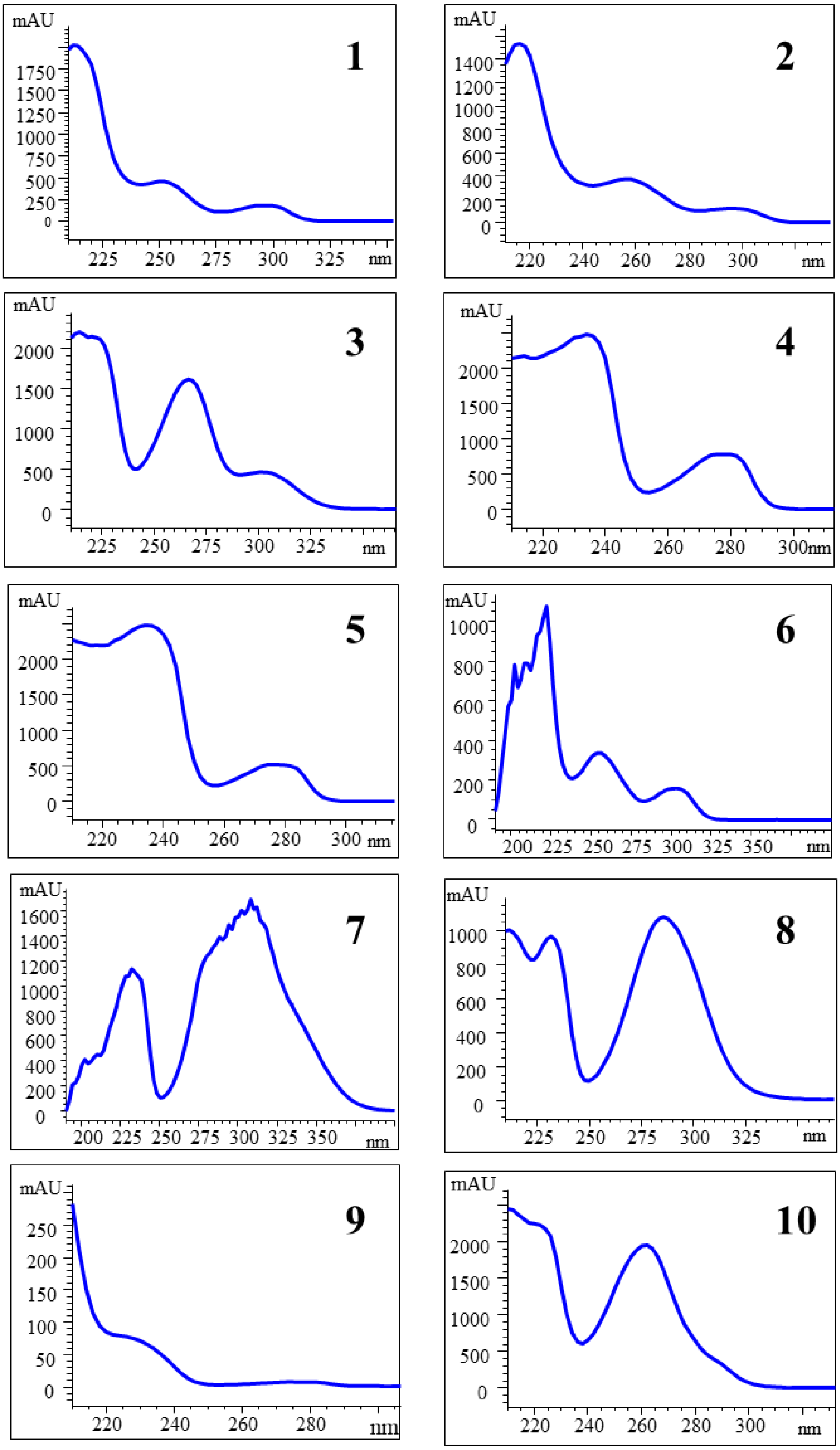

Figure S19: UV spectra of compounds **1-10**.

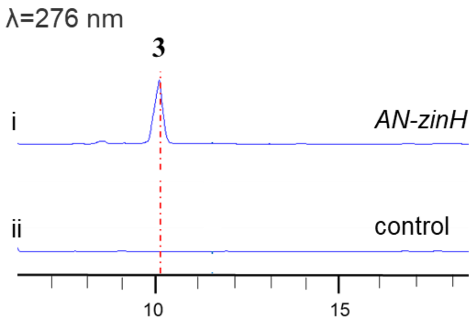

Figure S20: HPLC analysis of product from heterologous expression of *zinH* in *A. nidulans* A1145. The control in trace ii is *A. nidulans* A1145 transformed with empty vector only.

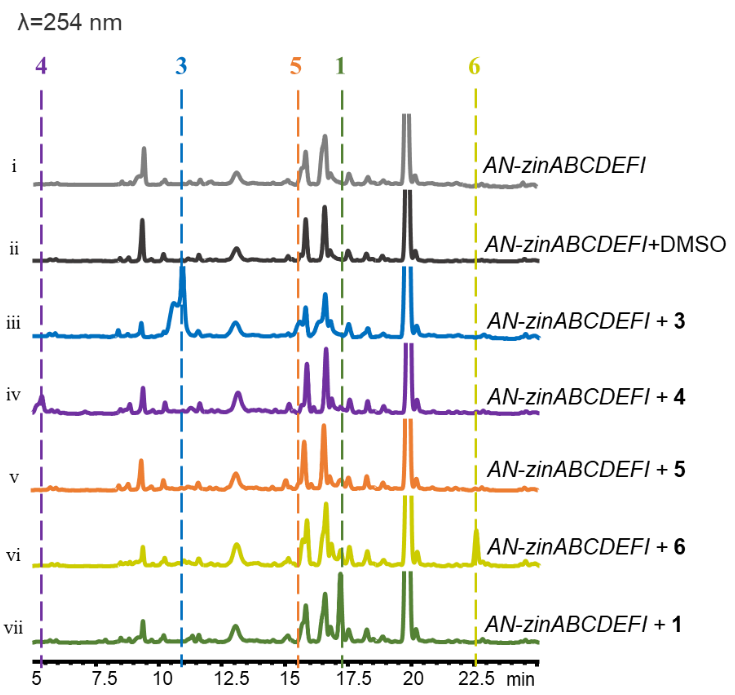

Figure S21: HPLC analysis of *AN-zinABCDEFI* strain cultures after feeding with **1**, **3**-**6**.

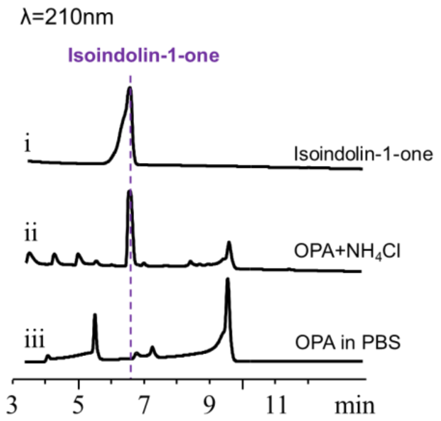

Figure S22: *O*-phthalaldehyde (OPA) reacts with NH_4Cl to form isoindolin-1-one by the non-enzymatic reaction.

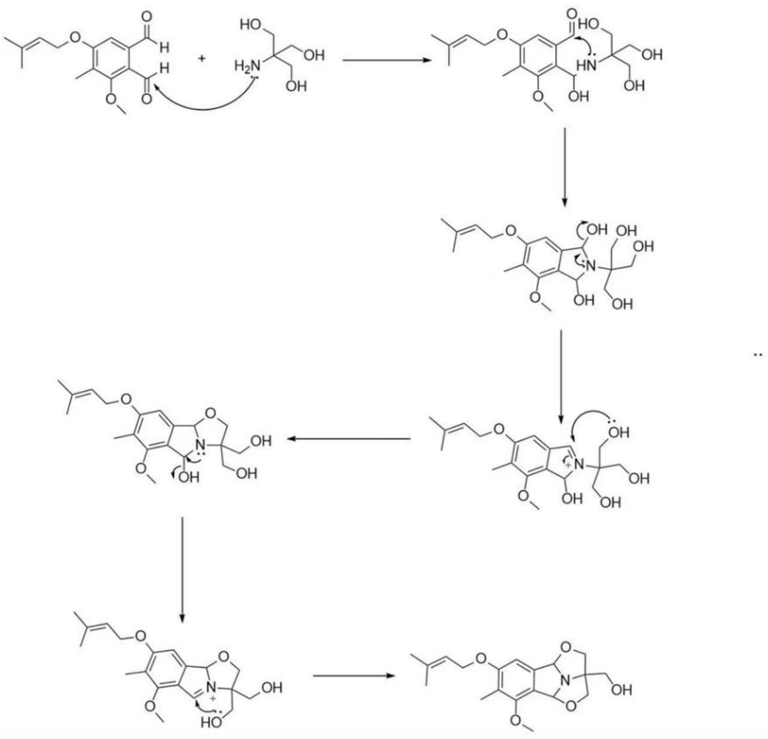

11

Figure S23: Proposed mechanism for the transformation of **11**.

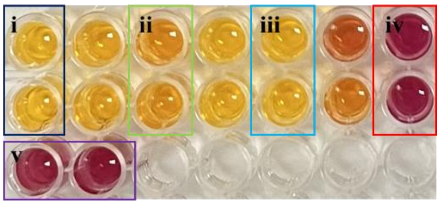

Figure S24: H_2O_2 level detection in ZinD-catalyzed reactions. i) H_2O_2 levels of PBS buffer (pH 7.0). ii) H_2O_2 levels of inactivated ZinD negative control group. iii) H_2O_2 levels of ZinD enzyme reactions with DMSO. iv) H_2O_2 levels of ZinD enzyme reactions with compound **5**. v) H_2O_2 levels of 80 μM or 100 μM H_2O_2.

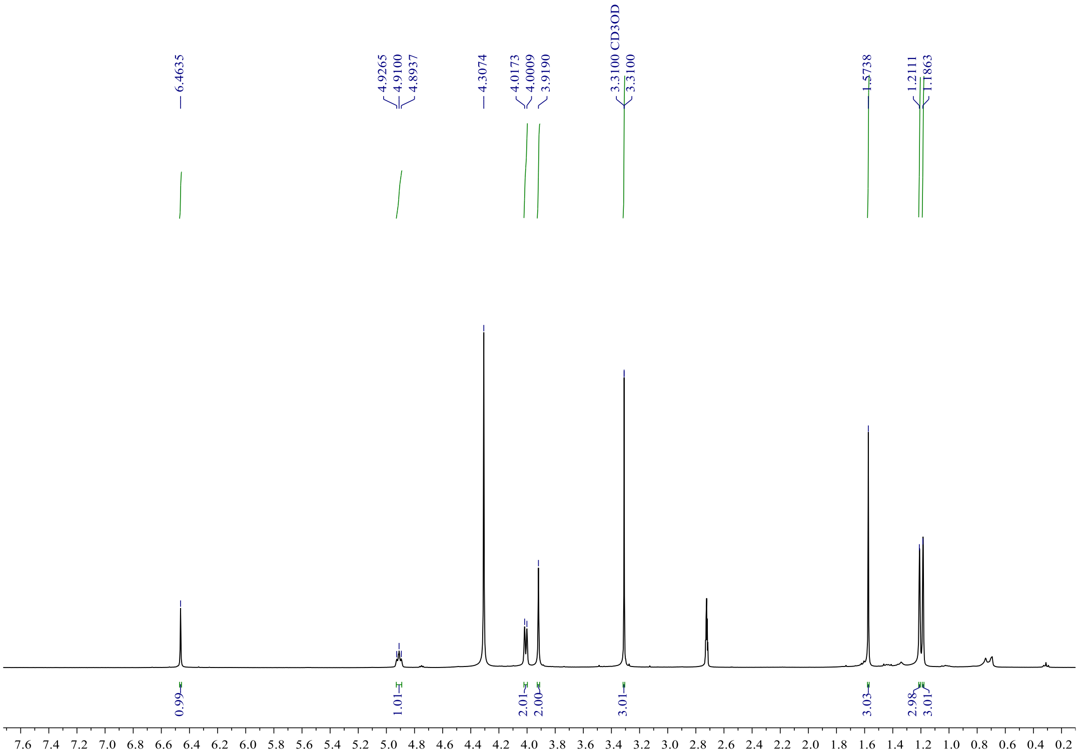

Figure S25: ^{1}H-NMR spectrum of **1** in CD_3OD (400 MHz).

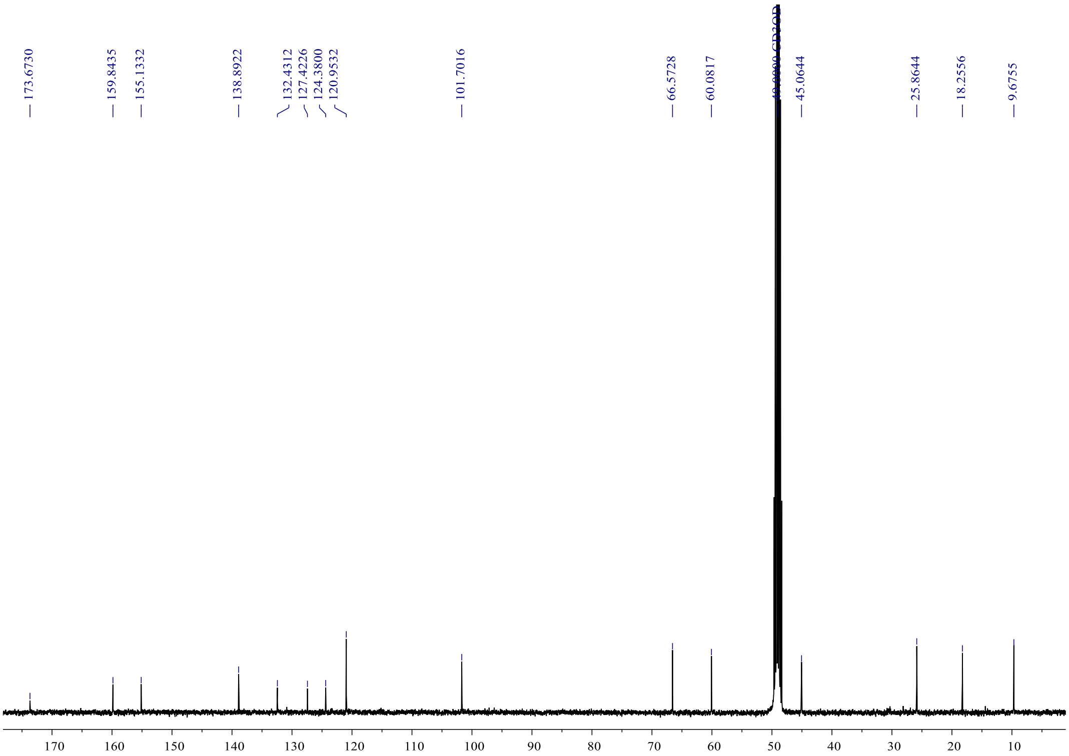

Figure S26: ^{13}C-NMR spectrum of **1** in CD_3OD (100 MHz).

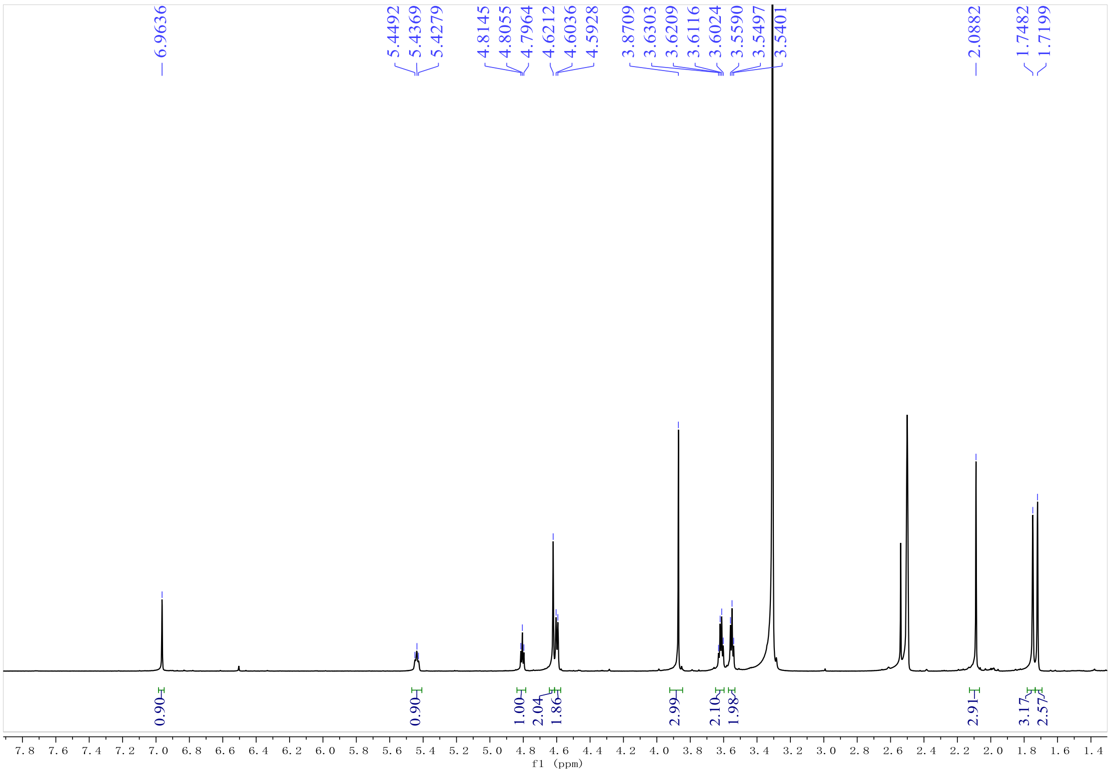

Figure S27: ^{1}H-NMR spectrum of **2** in DMSO-d_6 (600 MHz).

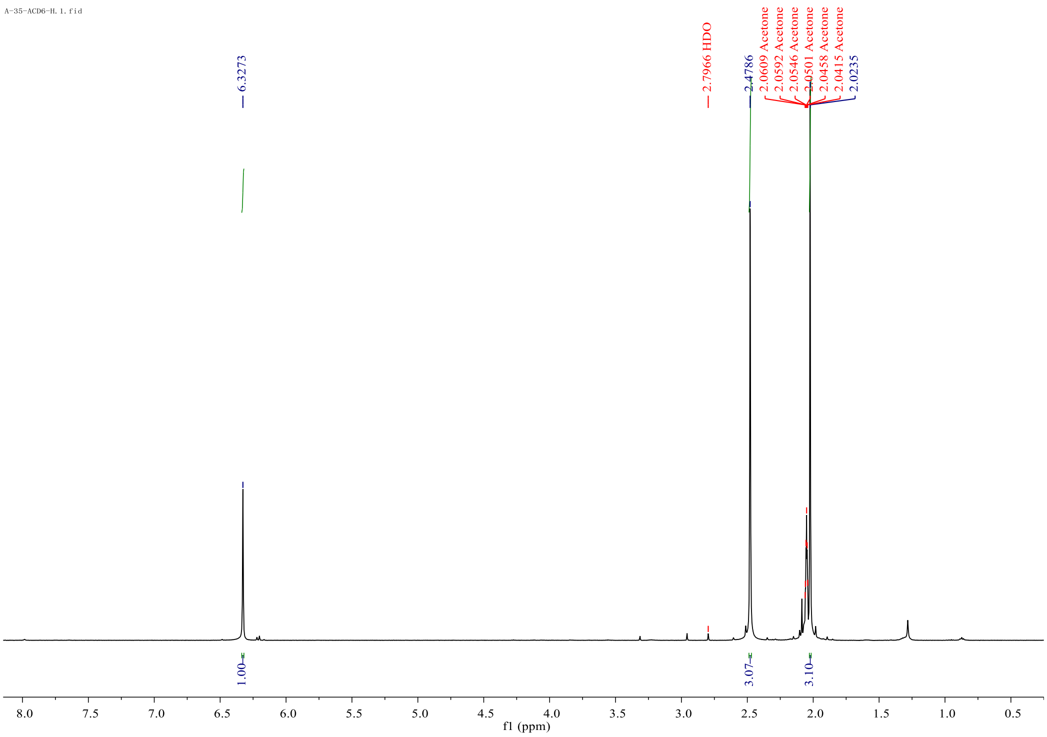

Figure S28: ^{1}H-NMR spectrum of **3** in acetone-d_6 (500 MHz).

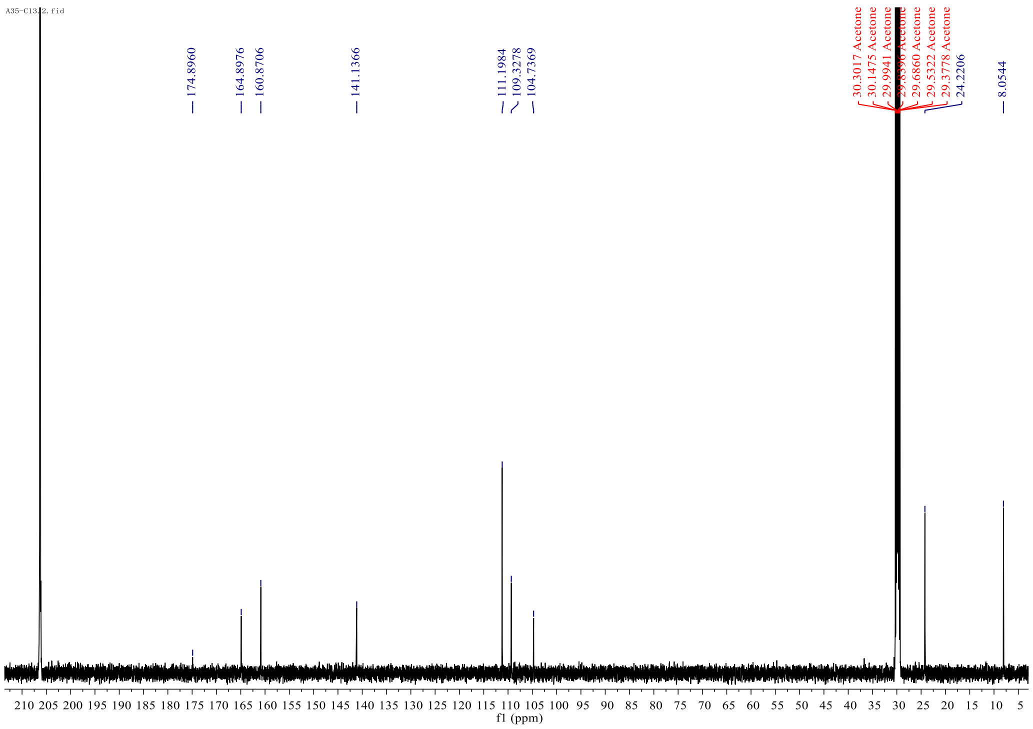

Figure S29: ^{13}C-NMR spectrum of **3** in acetone-d_6 (125 MHz).

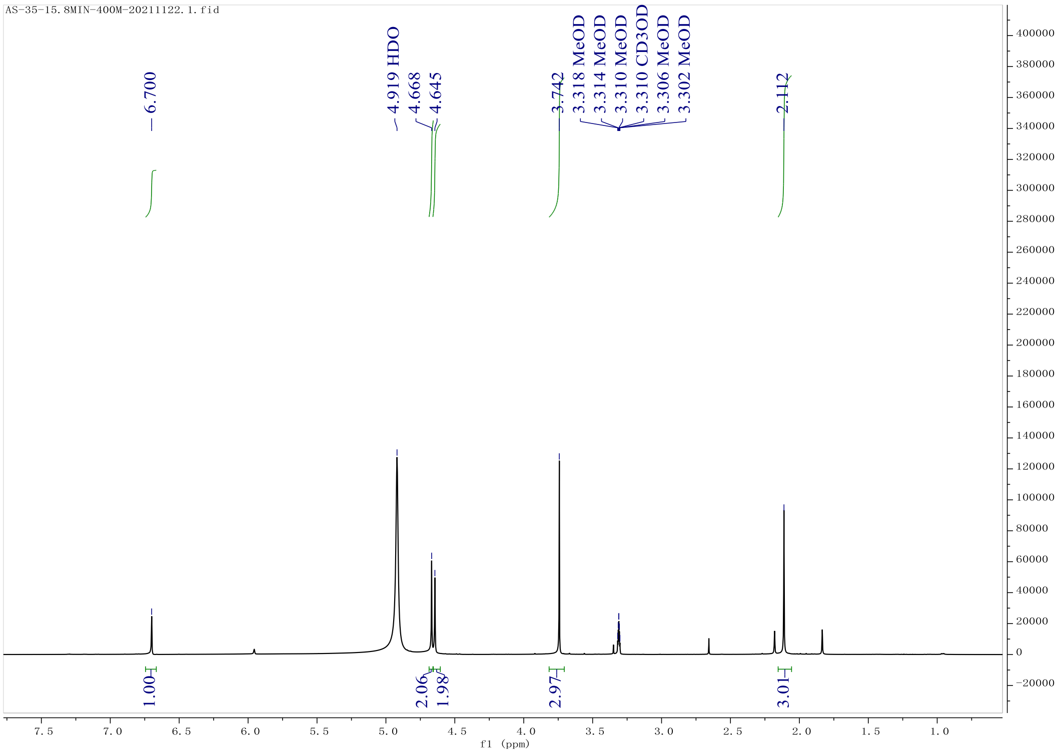

Figure S30: ^{1}H-NMR spectrum of **4** in CD_3OD (400 MHz).

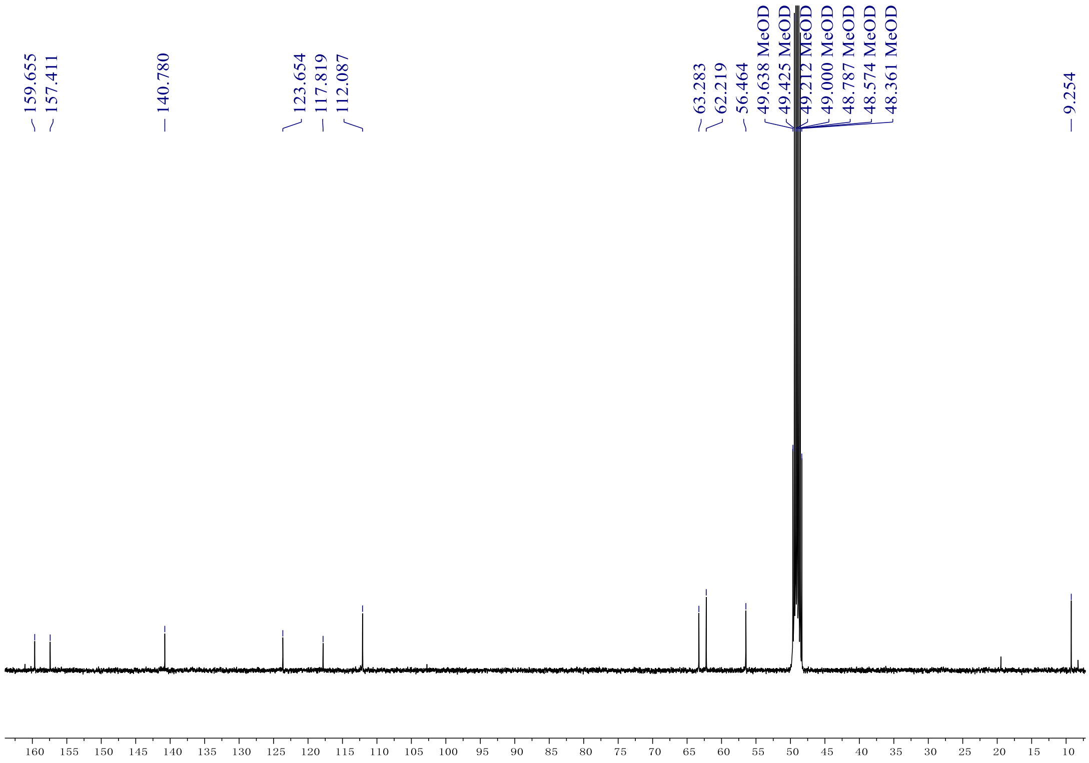

Figure S31: ^{13}C-NMR spectrum of **4** in CD_3OD(100 MHz).

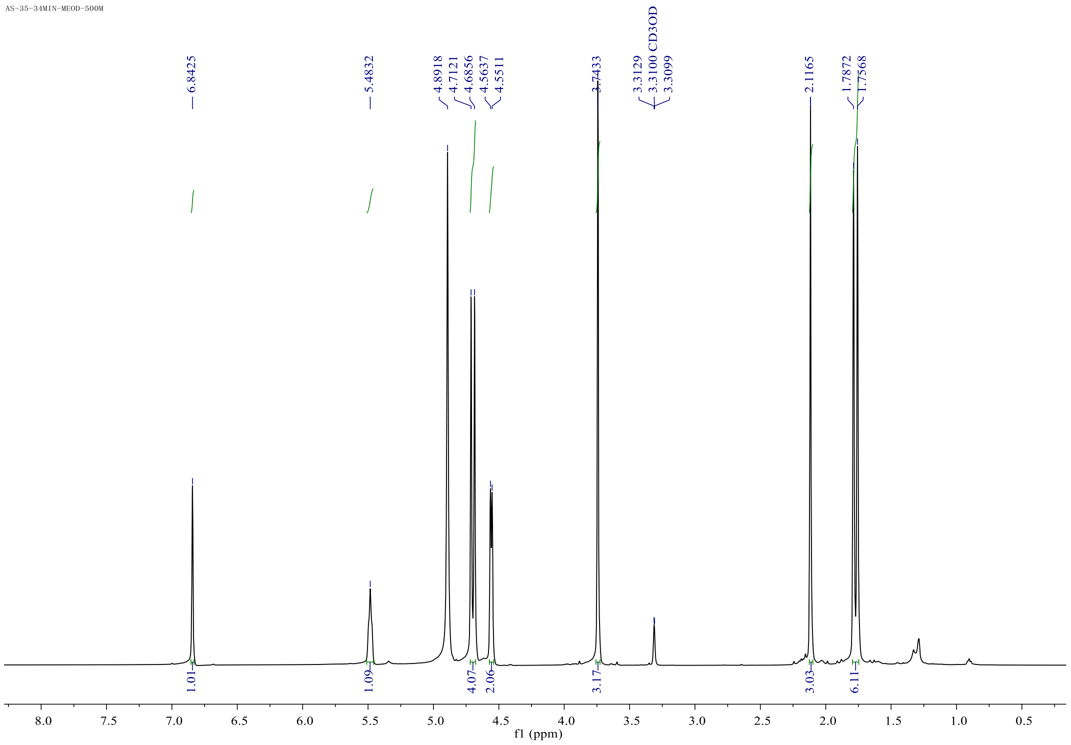

Figure S32: ^{1}H-NMR spectrum of **5** in CD_3OD (500 MHz).

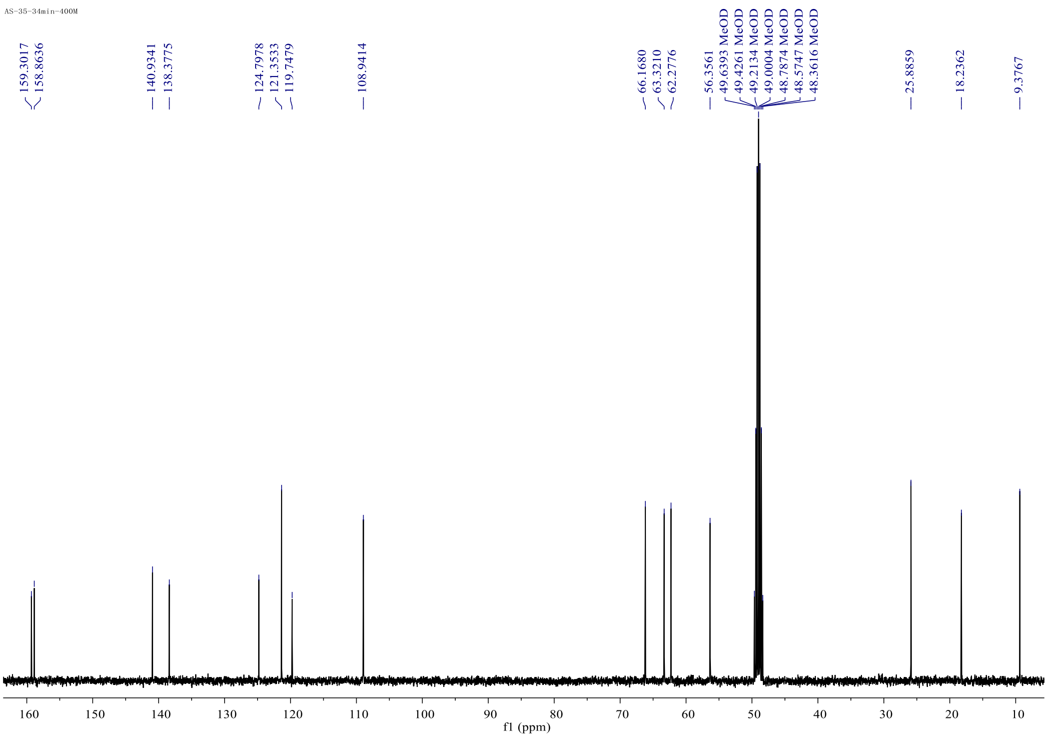

Figure S33: ^{13}C-NMR spectrum of **5** in CD_3OD (100 MHz).

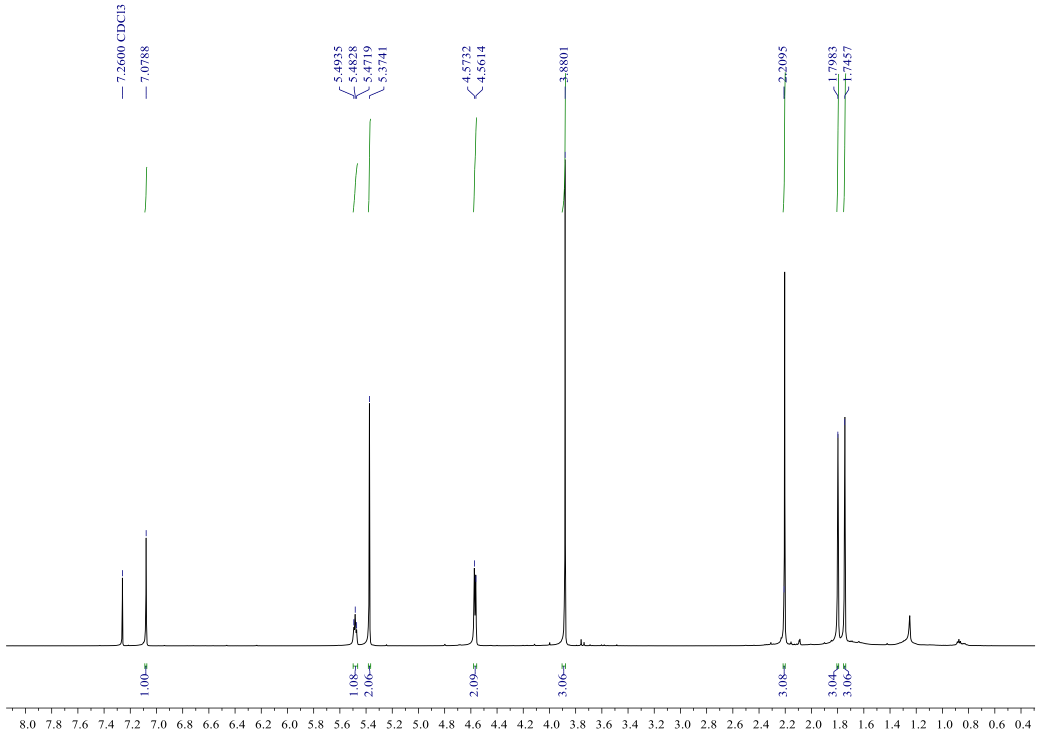

Figure S34: ^{1}H-NMR spectrum of **6** in $CDCl_3$ (600 MHz).

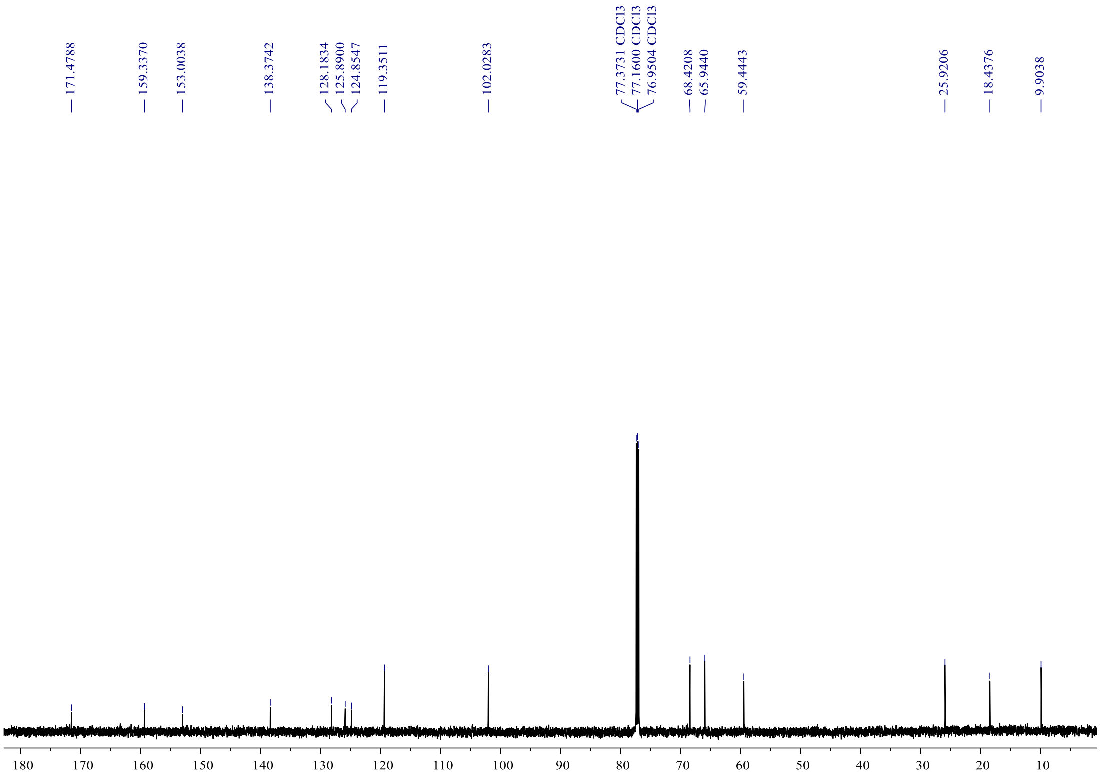

Figure S35: ^{13}C-NMR spectrum of **6** in $CDCl_3$ (150 MHz).

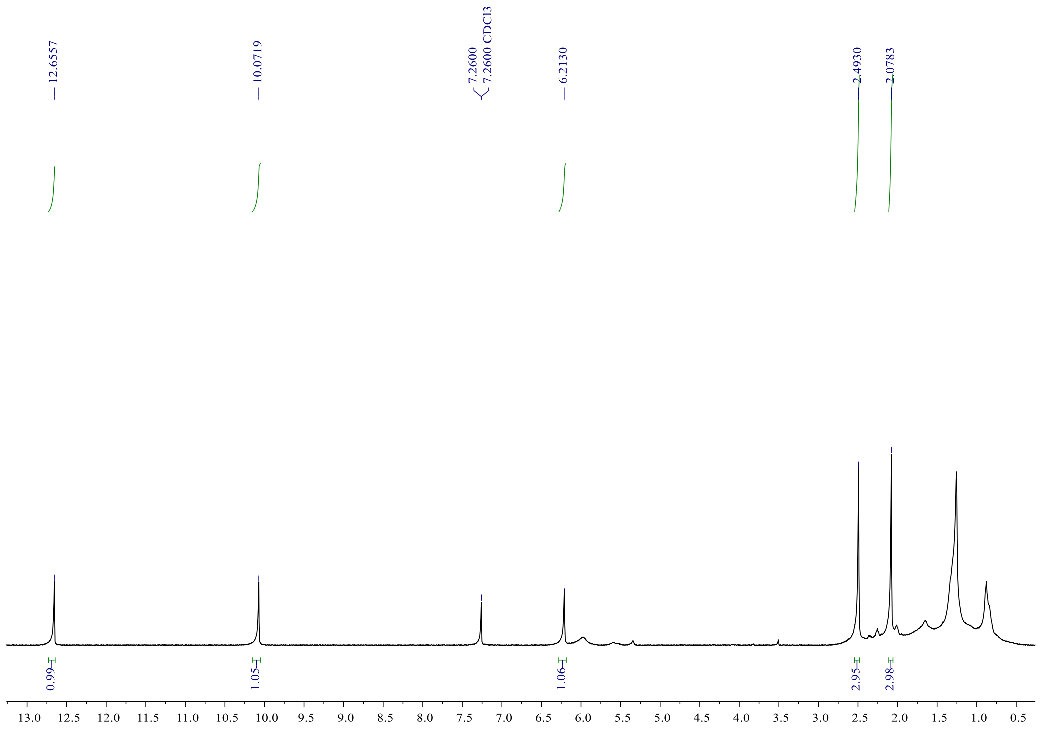

Figure S36: ^{1}H-NMR spectrum of **7** in $CDCl_3$ (500 MHz).

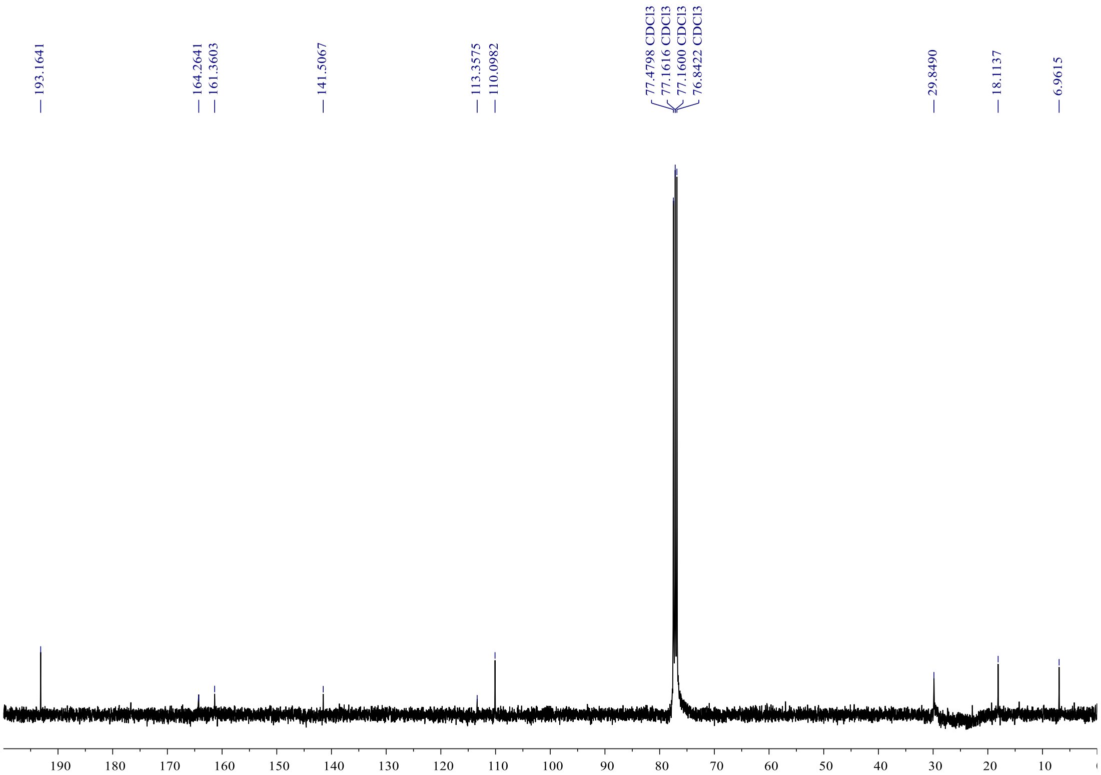

Figure S37: ^{13}C-NMR spectrum of **7** in $CDCl_3$ (100 MHz).

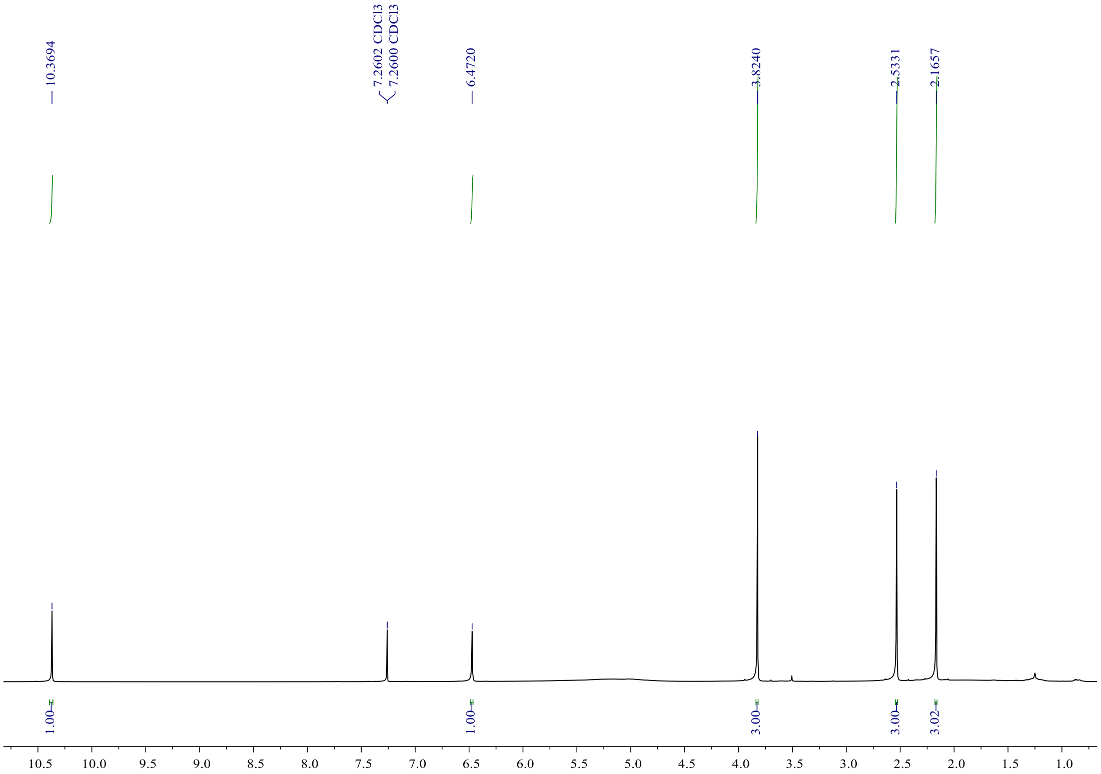

Figure S38: [1] H-NMR spectrum of **8** in $CDCl_3$ (600 MHz).

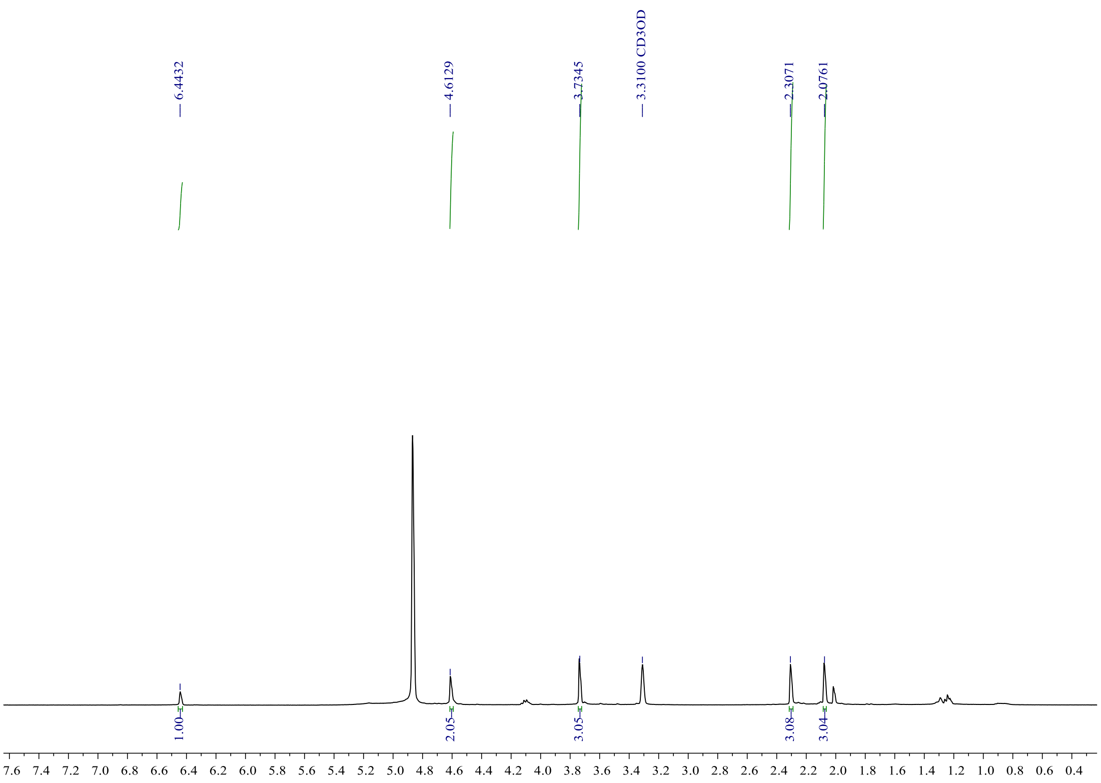

Figure S39: ^{1}H-NMR spectrum of **9** in CD_3OD (400 MHz).

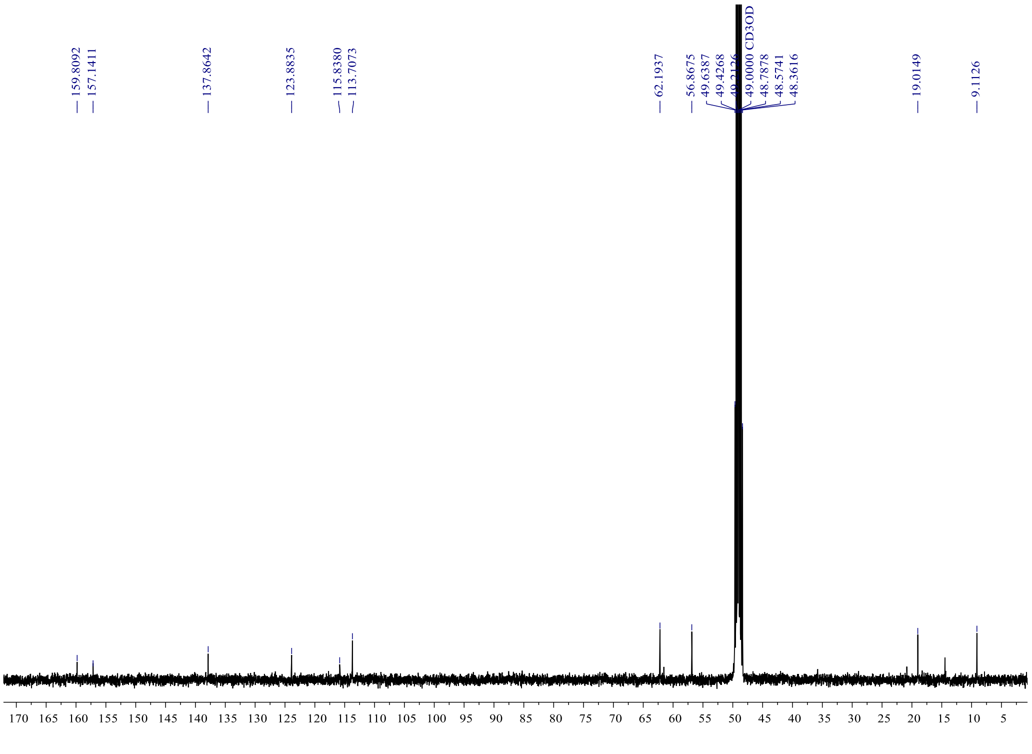

Figure S40: ^{13}C-NMR spectrum of **9** in CD_3OD (100 MHz).

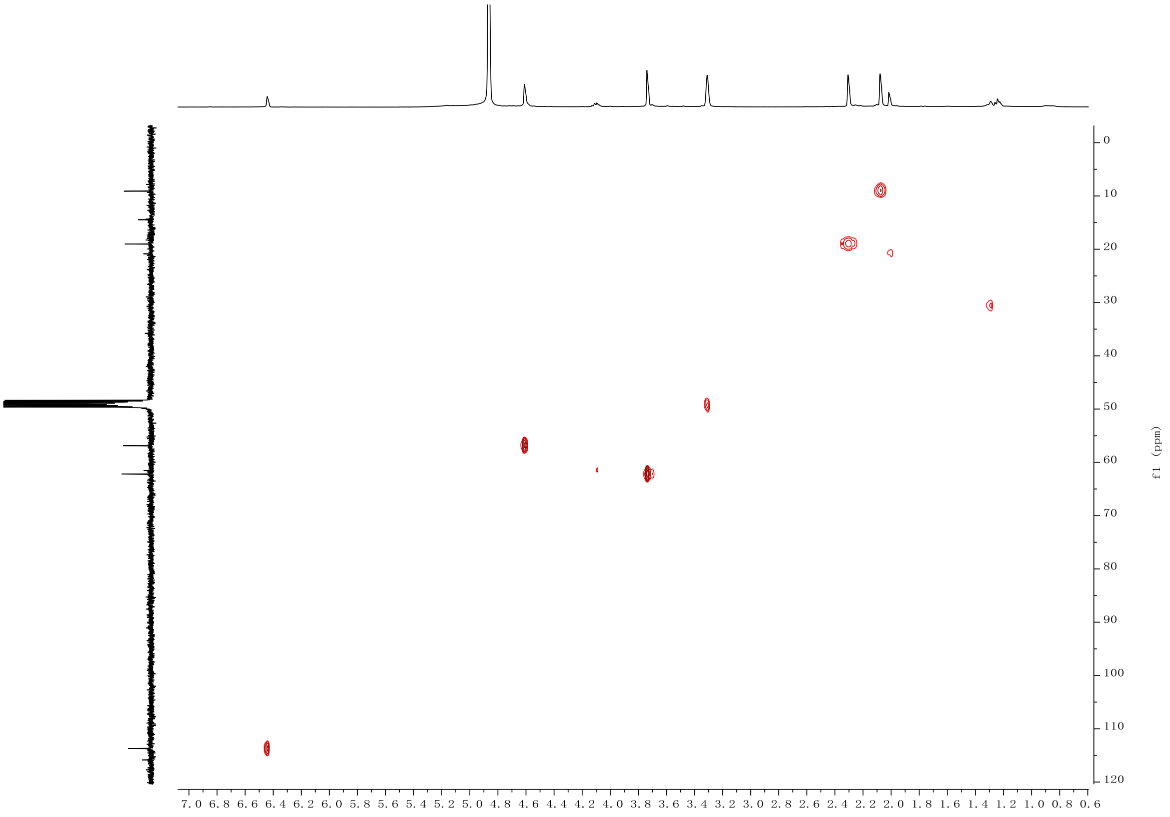

Figure S41: HSQC spectrum of **9** in CD_3OD (100 MHz).

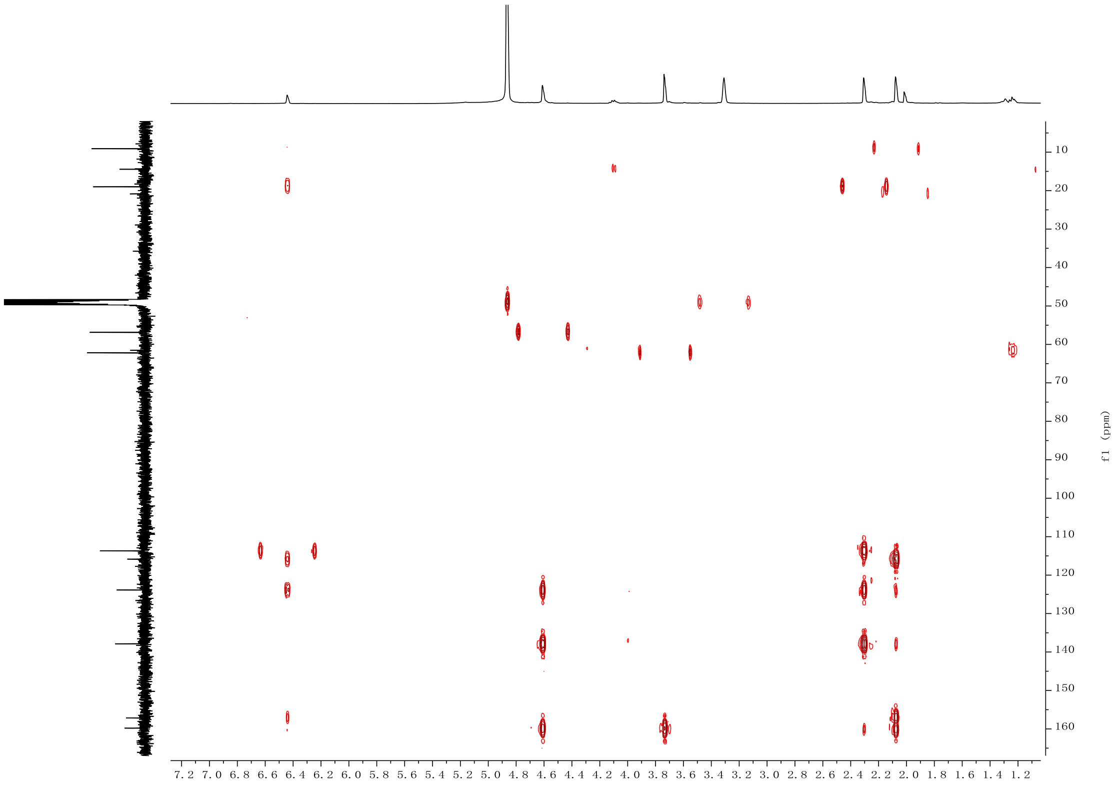

Figure S42: HMBC spectrum of **9** in CD_3OD (100 MHz).

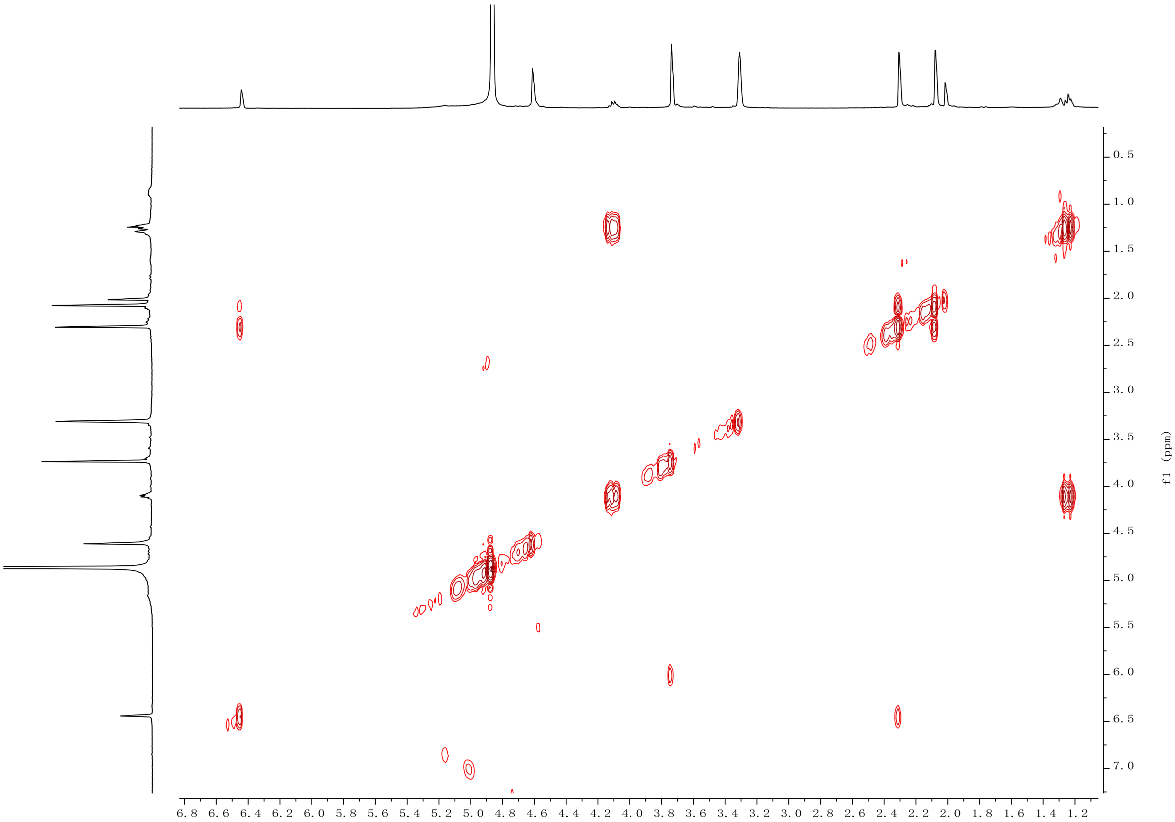

Figure S43: ^{1}H-^{1}H COSY spectrum of **9** in CD_3OD (400 MHz).

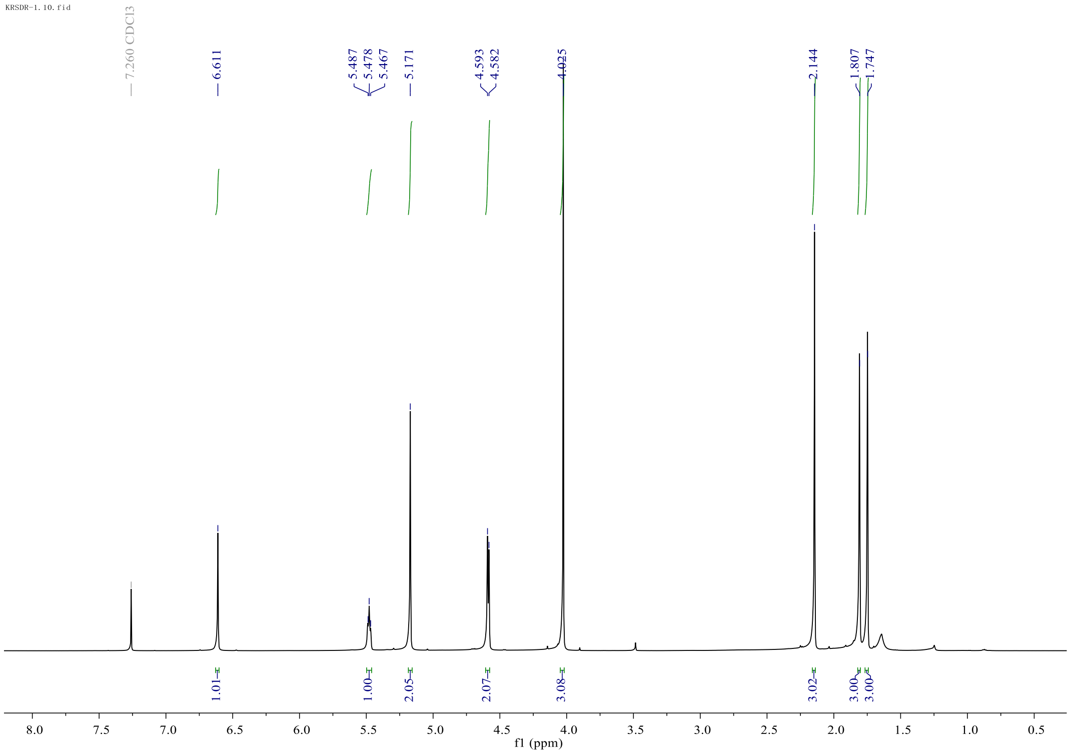

Figure S44: ^{1}H-NMR spectrum of **10** in $CDCl_3$ (600 MHz).

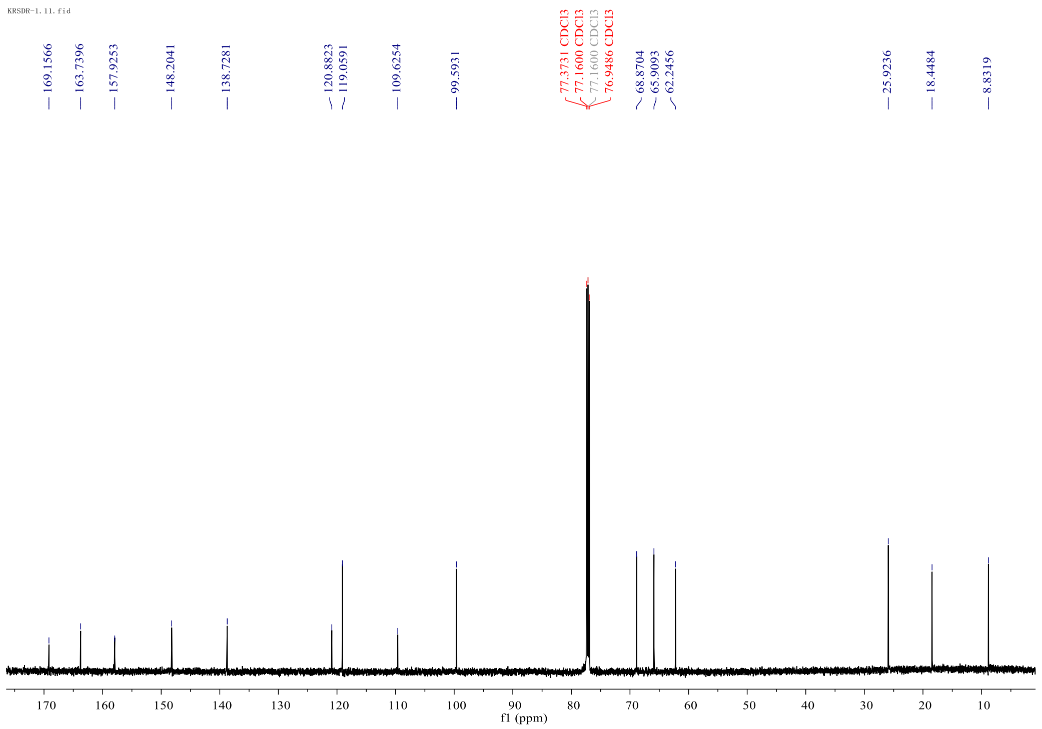

Figure S45: ^{13}C-NMR spectrum of **10** in $CDCl_3$ (150 MHz).

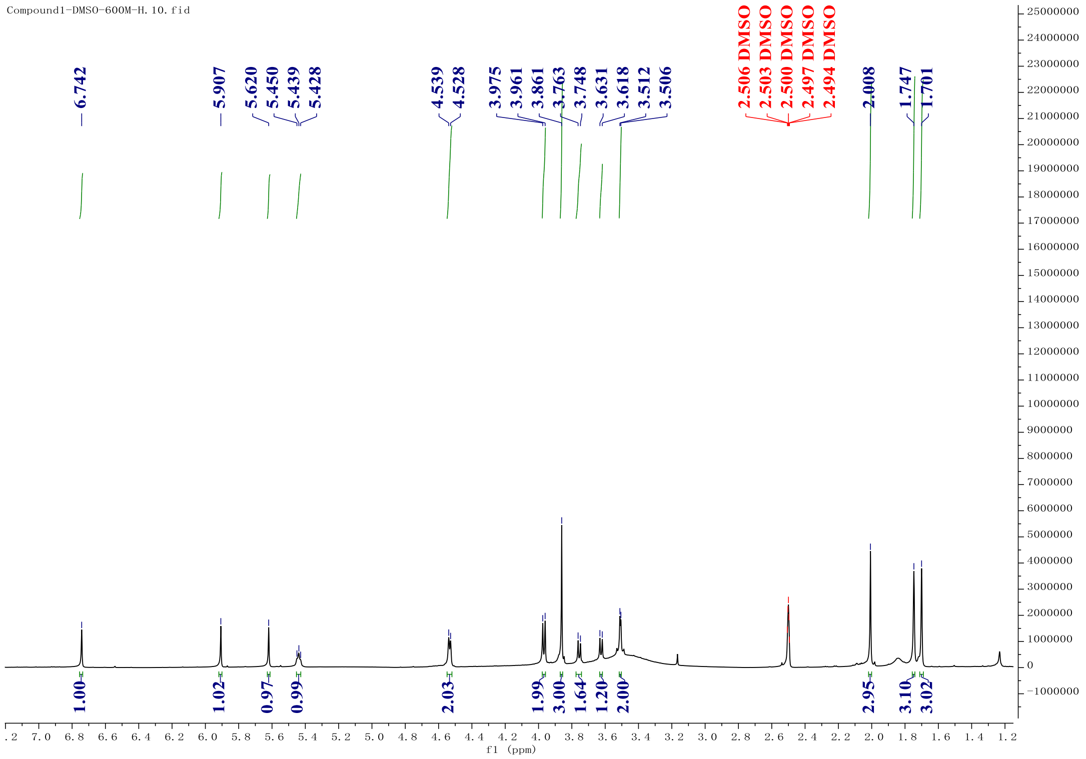

Figure S46: ^{1}H-NMR spectrum of **11** in DMSO-d_6 (600 MHz).

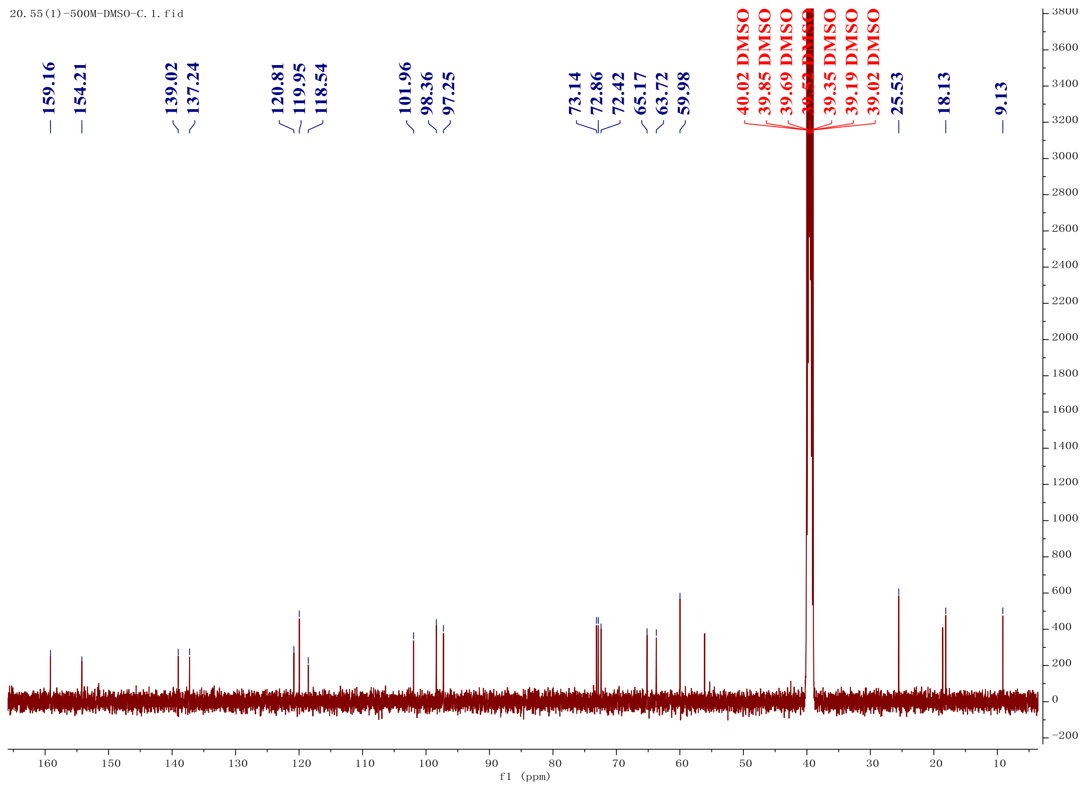

Figure S47: ^{13}C-NMR spectrum of **11** in DMSO-d_6 (125 MHz).

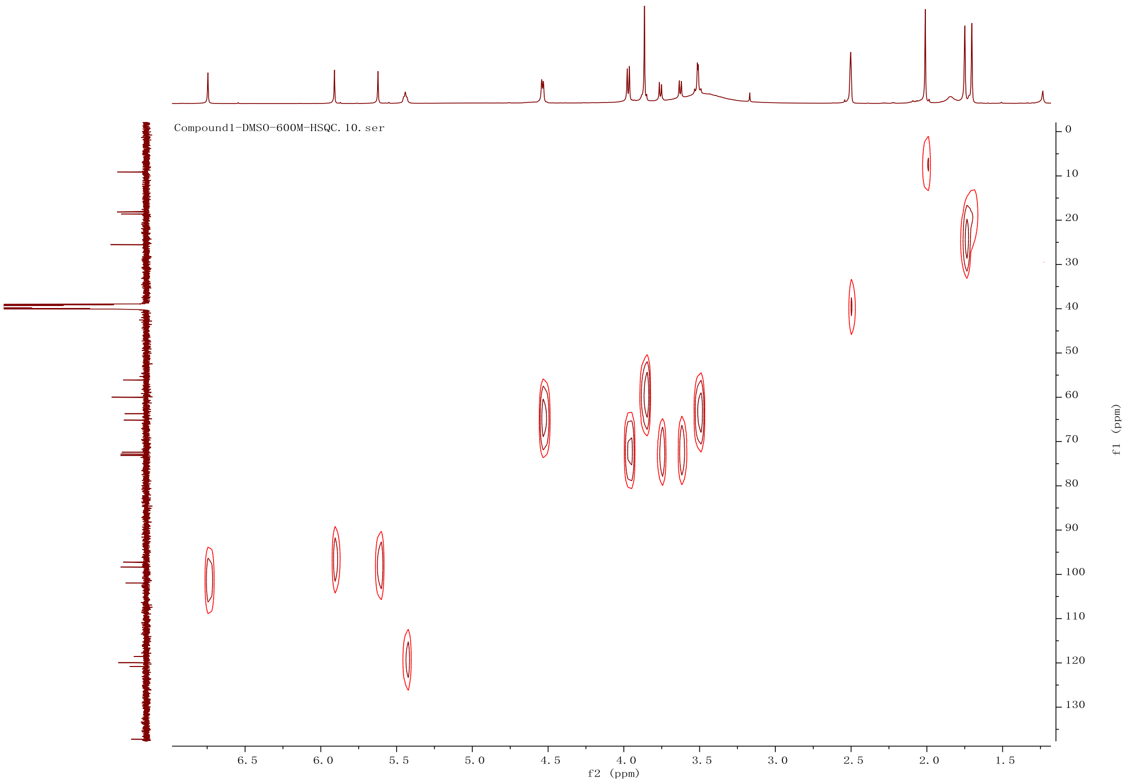

Figure S48: HSQC spectrum of **11** in DMSO-d_6 (125 MHz).

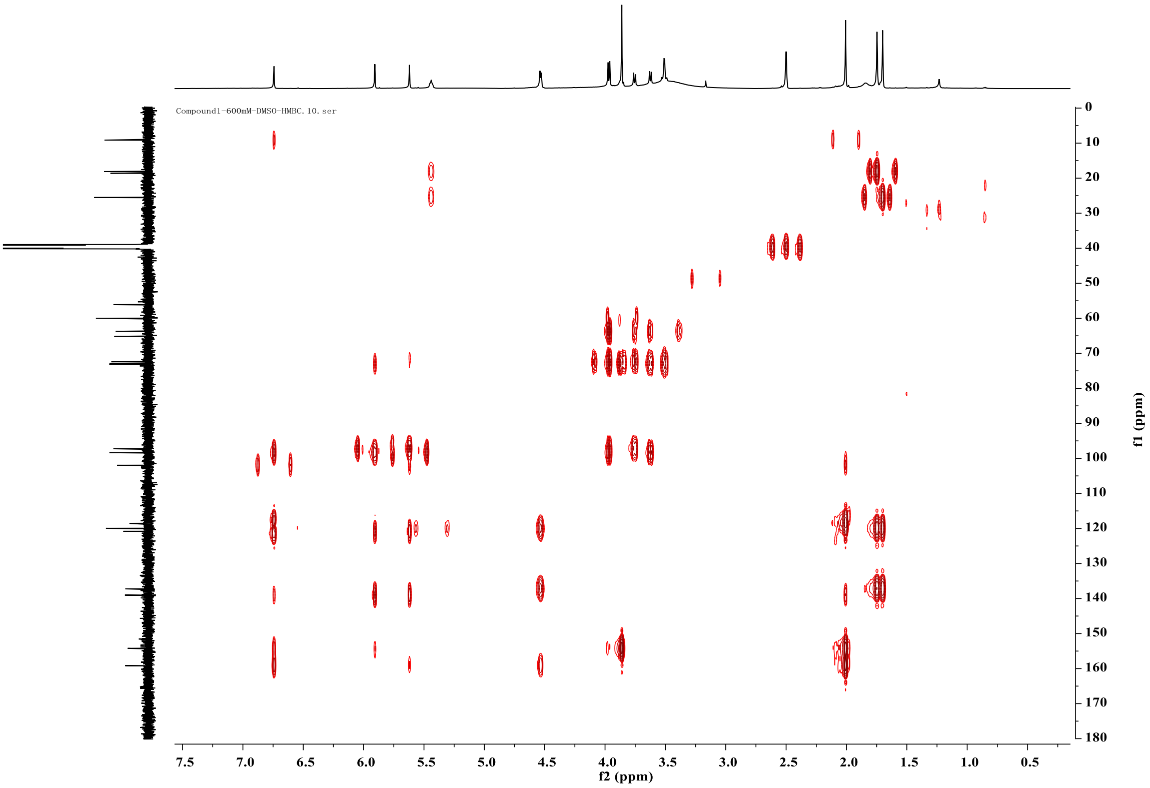

Figure S49: HMBC spectrum of **11** in DMSO-d_6 (125 MHz).

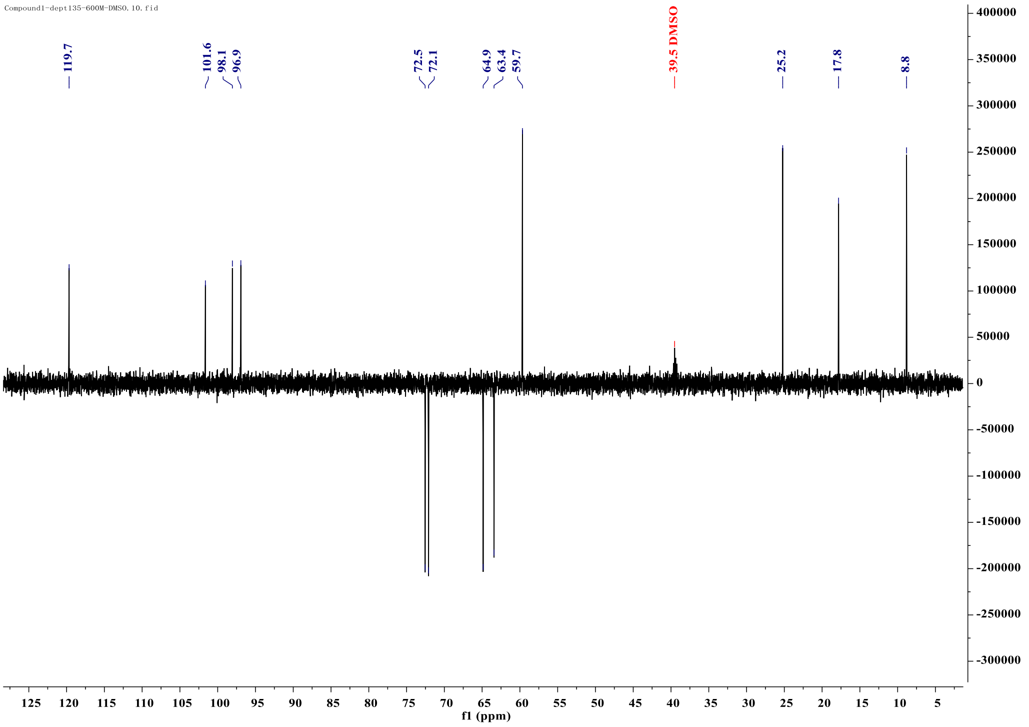

Figure S50: DEPT135 spectrum of **11** in DMSO-d_6 (125 MHz).

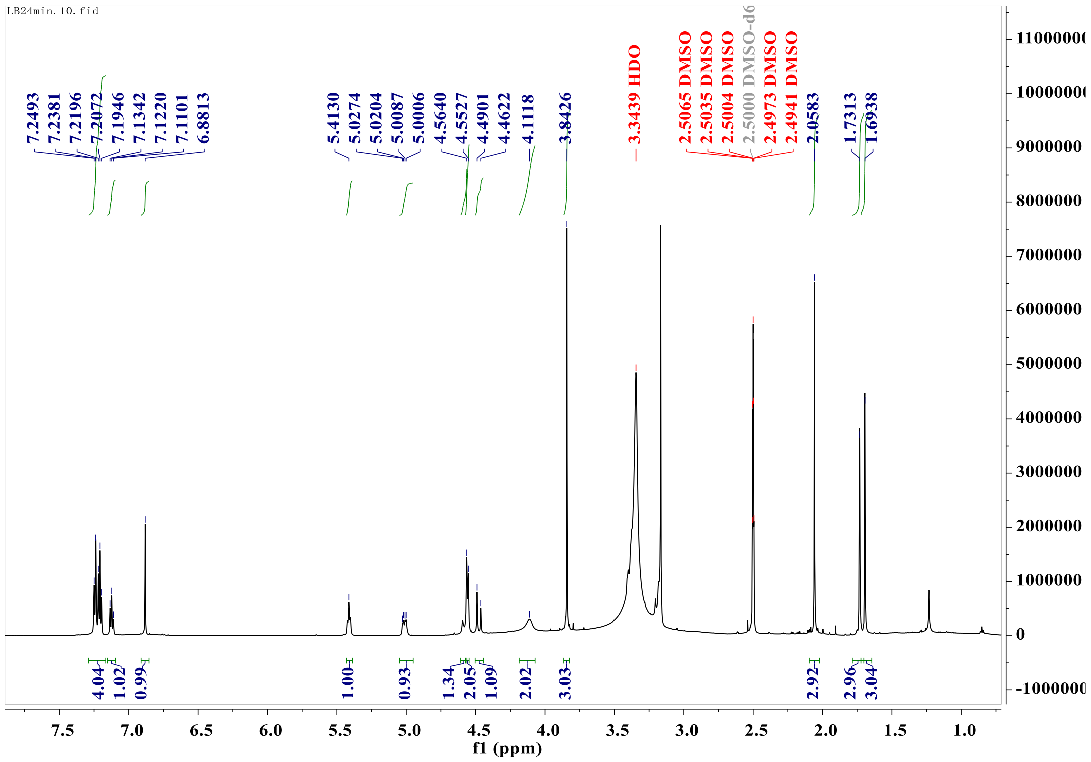

Figure S51: ^{1}H-NMR spectrum of **16** in DMSO-d_6 (600 MHz).

Supplementary Computation Data

Geometry data of conformers of structure 7*R*9*S*2"*R*-**11**

7*R*9*S*2"*R*-**11**-c30, ΔG = 0.0000 kcal/mol, population = 22.03 %

C 1.420366 -0.432712 -0.223124
C 0.842135 -1.550404 -0.851632
C -0.495192 -1.870995 -0.574880
C -1.242262 -1.072378 0.297253
C -0.620020 0.022723 0.911437
C 0.702042 0.352106 0.682342
O 2.720009 -0.196188 -0.555464
C 3.381384 0.952005 0.019942
C 4.746112 1.033778 -0.571744
C 5.909756 0.880895 0.067600
C 7.212364 1.017281 -0.672545
C 6.058259 0.576872 1.532307
C 1.669303 -2.373173 -1.799498
O -1.003283 -2.944691 -1.251298
C -2.004496 -3.748706 -0.617081
C -2.710346 -1.120790 0.743359
N -2.879003 0.161313 1.383939
C -1.627050 0.735701 1.812437
C -3.189073 2.345329 1.077488
O -1.845095 2.153317 1.645398
O -3.764087 -1.115180 -0.252006
C -4.464671 0.175292 -0.175871
C -3.314739 1.062033 0.272305
C -2.427458 1.270400 -0.995541
O -3.181634 2.190737 -1.802632
H 1.143618 1.206919 1.171769
H 2.804158 1.847996 -0.230243
H 3.402030 0.852427 1.105401
H 4.765602 1.245503 -1.636860
H 7.822878 1.813088 -0.234991
H 7.796441 0.095456 -0.593345
H 7.058926 1.240763 -1.728238
H 5.112421 0.407265 2.041970
H 6.681188 -0.312033 1.666203
H 6.574443 1.398195 2.038385
H 1.145767 -3.283225 -2.077899
H 1.894405 -1.812444 -2.710951
H 2.627262 -2.635233 -1.347562
H -2.043595 -4.674964 -1.185672
H -1.724319 -3.970263 0.414792
H -2.981134 -3.266319 -0.646268
H -2.904523 -1.948798 1.426577
H -1.417221 0.550259 2.868795
H -3.158371 3.251576 0.481749
H -3.927850 2.422465 1.875721
H -4.842853 0.392197 -1.169504
H -5.274248 0.124357 0.552591
H -1.460555 1.697835 -0.740452
H -2.275028 0.336529 -1.531816
H -2.683231 2.349974 -2.611734

7*R*9*S*2"*R*-**11**-c27, ΔG = 0.1820 kcal/mol, population = 16.20 %

C 1.412762 -0.413712 -0.217539
C 0.839860 -1.532989 -0.847769
C -0.494773 -1.863162 -0.568420
C -1.244344 -1.071686 0.307923
C -0.627153 0.024973 0.924029
C 0.692076 0.363504 0.692984
O 2.709390 -0.167385 -0.553735
C 3.367297 0.980507 0.027034
C 4.727463 1.077559 -0.573101
C 5.894985 0.891710 0.050343
C 7.192780 1.047722 -0.694227
C 6.051815 0.528302 1.500497
C 1.669486 -2.347296 -1.800723
O -0.997062 -2.938301 -1.246299
C -1.996609 -3.746900 -0.615317
C -2.713035 -1.125425 0.750097
N -2.887876 0.153810 1.395754
C -1.637312 0.731288 1.825178
C -3.205478 2.333170 1.098478
O -1.859355 2.149487 1.657245
O -3.761386 -1.118961 -0.250640
C -4.466497 0.168890 -0.173146
C -3.322441 1.057676 0.281074
C -2.431454 1.271290 -0.994786
O -3.150678 2.113327 -1.905278
H 1.129663 1.219351 1.184151
H 2.782884 1.874829 -0.211270
H 3.395804 0.871135 1.111444
H 4.740232 1.329610 -1.629459

H 7.813196 1.820229 -0.229662
H 7.769889 0.118899 -0.657295
H 7.032807 1.315096 -1.738685
H 6.666206 -0.371698 1.593691
H 6.580566 1.323340 2.034799
H 5.108132 0.347616 2.010353
H 2.629491 -2.606616 -1.351507
H 1.150127 -3.258714 -2.082399
H 1.890172 -1.781098 -2.709811
H -2.030790 -4.672548 -1.185181
H -1.717921 -3.968598 0.416922
H -2.974952 -3.268115 -0.646252
H -2.907858 -1.956708 1.429135
H -1.427733 0.547783 2.881820
H -3.188851 3.258889 0.529703
H -3.943922 2.395755 1.898274
H -4.842279 0.387594 -1.167488
H -5.279837 0.112053 0.550787
H -1.468809 1.709524 -0.746357
H -2.266538 0.335393 -1.516458
H -2.988052 3.034458 -1.675744

7*R*9*S*2"*R*-**11**-c31, ΔG = 0.2761 kcal/mol, population = 13.81 %

C 1.488252 -0.584122 0.197401
C 0.937692 -1.786485 -0.281433
C -0.426975 -2.040336 -0.076824
C -1.225664 -1.099459 0.581330
C -0.629981 0.077063 1.055396
C 0.714104 0.350225 0.890220
O 2.814697 -0.412934 -0.062254
C 3.474067 0.760255 0.464422
C 4.922204 0.658834 0.129933
C 5.603226 1.425749 -0.727118
C 7.076321 1.209304 -0.941821
C 5.016389 2.546386 -1.539179
C 1.815974 -2.771479 -1.001149
O -0.899019 -3.206894 -0.609566
C -1.982824 -3.877107 0.044106
C -2.727054 -1.055899 0.901168
N -2.916442 0.318483 1.299511
C -1.690254 0.943778 1.731809
C -3.141613 2.421747 0.600975
O -1.855786 2.314313 1.308492
O -3.694262 -1.208207 -0.167815
C -4.364182 0.084471 -0.373374
C -3.233107 1.021163 0.017489
C -2.237168 0.998304 -1.185006
O -2.895544 1.776919 -2.198666
H 1.131354 1.274966 1.258826
H 3.011149 1.653570 0.044802
H 3.334910 0.780808 1.549959
H 5.455357 -0.129876 0.652669
H 7.459679 0.390877 -0.332621
H 7.282306 0.986728 -1.993151
H 7.638412 2.116042 -0.698167
H 5.228875 2.386622 -2.600107
H 3.940302 2.654129 -1.422996
H 5.485492 3.496633 -1.267040
H 2.033504 -2.435232 -2.019352
H 2.773942 -2.876797 -0.491561
H 1.333014 -3.742858 -1.064298
H -2.934317 -3.386677 -0.158828
H -2.000236 -4.882901 -0.369158
H -1.808900 -3.932376 1.120693
H -2.997795 -1.753549 1.694836
H -1.574497 0.939272 2.818469
H -3.037071 3.211552 -0.135436
H -3.942686 2.643350 1.306741
H -4.650704 0.132403 -1.418859
H -5.233878 0.168512 0.278801
H -1.283408 1.450236 -0.923496
H -2.066809 -0.015645 -1.539580
H -2.320104 1.801870 -2.971070

7*R*9*S*2"*R*-**11**-c29, ΔG = 0.3451 kcal/mol, population = 12.29 %

C 1.478911 -0.574338 0.176346
C 0.928884 -1.778729 -0.297639
C -0.432510 -2.038947 -0.079025
C -1.228122 -1.101862 0.588002
C -0.633221 0.077156 1.056317
C 0.707403 0.356908 0.876771
O 2.802015 -0.397892 -0.095126
C 3.463807 0.773842 0.431949
C 4.910737 0.671691 0.092682
C 5.591608 1.443340 -0.760190
C 7.063814 1.225236 -0.979371

C 5.005630 2.571469 -1.562397
C 1.804278 -2.759654 -1.026275
O -0.904652 -3.207689 -0.606495
C -1.977413 -3.883722 0.059298
C -2.725183 -1.064641 0.922040
N -2.919168 0.308713 1.322833
C -1.690839 0.940480 1.740625
C -3.158182 2.410070 0.619905
O -1.864589 2.308654 1.313983
O -3.699637 -1.220703 -0.141238
C -4.381811 0.065019 -0.328873
C -3.250061 1.009873 0.038959
C -2.259987 0.996689 -1.180406
O -2.808672 1.826687 -2.212361
H 1.124151 1.283813 1.240477
H 3.000071 1.668401 0.015924
H 3.328292 0.791835 1.517995
H 5.443215 -0.121978 0.608595
H 7.446460 0.401280 -0.377196
H 7.267341 1.010320 -2.032764
H 7.628384 2.128809 -0.729747
H 5.217624 2.420775 -2.624728
H 3.929693 2.679261 -1.444863
H 5.475713 3.518870 -1.282120
H 2.011106 -2.421487 -2.046056
H 2.767278 -2.861622 -0.525577
H 1.324520 -3.732817 -1.085853
H -1.792012 -3.936524 1.134064
H -2.934065 -3.399593 -0.134379
H -1.992987 -4.890109 -0.352551
H -2.987785 -1.764537 1.716374
H -1.564312 0.939875 2.826117
H -3.064616 3.199977 -0.118050
H -3.952270 2.631049 1.333800
H -4.709809 0.097981 -1.364446
H -5.238924 0.144908 0.340211
H -1.306110 1.438950 -0.917349
H -2.090195 -0.011738 -1.547952
H -3.450297 1.316539 -2.718348

7*R*9*S*2"*R*-**11**-c28, ΔG = 0.6231 kcal/mol, population = 7.69 %

C 1.478067 -0.581422 0.179253
C 0.926146 -1.783384 -0.298461
C -0.436110 -2.041733 -0.082003
C -1.230815 -1.105384 0.587143
C -0.633703 0.071119 1.058998
C 0.707698 0.348901 0.882066
O 2.801634 -0.405949 -0.091106
C 3.464912 0.762363 0.441326
C 4.911485 0.660429 0.100535
C 5.594568 1.440595 -0.742745
C 7.066265 1.221344 -0.964217
C 5.011987 2.580375 -1.530890
C 1.799889 -2.764005 -1.029495
O -0.909051 -3.207982 -0.613911
C -1.988719 -3.881167 0.043537
C -2.729889 -1.064626 0.916790
N -2.919357 0.307882 1.323235
C -1.689538 0.933165 1.745825
C -3.153993 2.409627 0.637988
O -1.859359 2.304830 1.323974
O -3.701223 -1.213841 -0.148529
C -4.375429 0.078057 -0.344394
C -3.244886 1.015117 0.041530
C -2.256143 0.995106 -1.178858
O -2.882980 1.677801 -2.272525
H 1.125898 1.273598 1.249692
H 3.002009 1.659619 0.030210
H 3.330039 0.775035 1.527579
H 5.441773 -0.140639 0.607200
H 7.269348 1.017789 -2.019956
H 7.633077 2.120635 -0.704417
H 7.446816 0.389719 -0.371341
H 3.935835 2.687662 -1.414863
H 5.482148 3.523291 -1.235934
H 5.226941 2.444488 -2.594600
H 2.765275 -2.863448 -0.533054
H 1.321410 -3.738044 -1.085511
H 2.001265 -2.427318 -2.050893
H -2.007041 -4.885808 -0.372429
H -1.809147 -3.939153 1.119043
H -2.941716 -3.391203 -0.153276
H -2.995419 -1.766362 1.708619
H -1.564801 0.929299 2.831408
H -3.068294 3.223816 -0.076458
H -3.948454 2.619329 1.354750
H -4.669798 0.129423 -1.387640

H -5.241874 0.156533 0.312886
H -1.303021 1.456031 -0.934833
H -2.078404 -0.018535 -1.519394
H -2.724161 2.623847 -2.185213

7*R*9*S*2"*R*-**11**-c15, ΔG = 0.8685 kcal/mol, population = 5.08 %

C 1.720933 -0.783169 -0.580032
C 1.047930 -2.007283 -0.425077
C -0.247407 -2.011419 0.117418
C -0.857616 -0.808915 0.485076
C -0.141273 0.385818 0.326799
C 1.139719 0.423875 -0.184562
O 2.956668 -0.873333 -1.150729
C 3.836543 0.273507 -1.119097
C 4.330270 0.551344 0.263980
C 4.317009 1.714953 0.919594
C 4.859933 1.812153 2.318941
C 3.781900 3.007354 0.369744
C 1.723572 -3.285396 -0.836631
O -0.852661 -3.234318 0.195477
C -1.783371 -3.477736 1.256251
C -2.246766 -0.478354 1.046894
N -2.309630 0.955894 0.894010
C -1.002816 1.558400 0.789785
C -2.633111 2.637485 -0.532189
O -1.224012 2.666776 -0.108730
O -3.431883 -0.920394 0.336225
C -4.099985 0.261922 -0.220023
C -2.888160 1.141382 -0.477559
C -2.184031 0.550138 -1.750583
O -2.920220 0.970096 -2.906591
H 1.651867 1.365708 -0.294236
H 4.658200 -0.029643 -1.768765
H 3.347921 1.130456 -1.582599
H 4.746681 -0.318972 0.764127
H 5.231384 0.852216 2.677467
H 5.675045 2.540315 2.370307
H 4.083444 2.162489 3.006031
H 4.569386 3.766438 0.359403
H 3.390922 2.925393 -0.642293
H 2.987313 3.391223 1.016737
H 2.770660 -3.279503 -0.533722
H 1.224589 -4.143894 -0.395054
H 1.707741 -3.408678 -1.923702
H -1.915342 -4.556642 1.291753
H -1.380092 -3.131926 2.210271
H -2.744162 -3.002897 1.059956
H -2.352933 -0.784895 2.088327
H -0.647344 1.962631 1.740863
H -2.677327 3.067363 -1.527550
H -3.248562 3.203185 0.167982
H -4.641378 -0.068527 -1.102563
H -4.790615 0.689217 0.507380
H -1.185462 0.954524 -1.869329
H -2.121746 -0.533923 -1.708253
H -3.668336 0.376874 -3.034558

7*R*9*S*2"*R*-**11**-c5, ΔG = 1.0228 kcal/mol, population = 3.91 %

C 1.848820 -1.219306 0.681737
C 1.175579 -2.249357 0.013258
C -0.232683 -2.248613 -0.000001
C -0.941427 -1.201312 0.589024
C -0.221646 -0.210834 1.279537
C 1.151404 -0.218490 1.363384
O 3.218959 -1.247514 0.650331
C 3.910986 0.030787 0.612706
C 3.481258 0.845784 -0.563173
C 2.925771 2.061465 -0.558257
C 2.511621 2.718793 -1.846625
C 2.646084 2.882598 0.669009
C 1.920830 -3.341563 -0.701858
O -0.792780 -3.285798 -0.686595
C -2.138031 -3.672832 -0.388131
C -2.429667 -0.817359 0.594681
N -2.391777 0.546718 1.066896
C -1.189458 0.844881 1.806605
C -1.973907 2.669716 0.531876
O -0.945487 2.232291 1.487664
O -3.135092 -0.690113 -0.665952
C -3.427797 0.732683 -0.893826
C -2.237393 1.354730 -0.184561
C -1.020021 1.144265 -1.140083
O -1.215509 2.116960 -2.181487
H 1.669852 0.566995 1.889770
H 3.787502 0.551656 1.561711
H 4.958018 -0.260062 0.528299

H 3.620409 0.348520 -1.519452
H 2.662975 2.064025 -2.704888
H 1.456076 3.004151 -1.807680
H 3.076350 3.641698 -2.010007
H 1.565962 2.992285 0.806383
H 3.063088 2.460236 1.580616
H 3.057706 3.888350 0.548660
H 1.638165 -3.381370 -1.756111
H 2.993144 -3.182524 -0.633124
H 1.681985 -4.318754 -0.275786
H -2.274234 -3.789881 0.689017
H -2.855569 -2.954179 -0.782284
H -2.282588 -4.634014 -0.875473
H -3.024281 -1.460245 1.244529
H -1.327638 0.767065 2.887588
H -1.527319 3.440658 -0.087310
H -2.847154 3.048265 1.063960
H -3.444515 0.886828 -1.967866
H -4.383327 0.999968 -0.441947
H -0.074938 1.321989 -0.634141
H -1.015320 0.141266 -1.558948
H -0.586518 1.925887 -2.885871

7*R*9*S*2''*R*-**11**-c37, ΔG = 1.1013 kcal/mol, population = 3.43 %

C 1.893971 -1.085389 0.914733
C 1.356460 -2.200688 0.262739
C -0.040706 -2.292436 0.104657
C -0.866179 -1.253195 0.533227
C -0.282032 -0.168389 1.210185
C 1.072458 -0.080197 1.432736
O 3.258420 -1.033172 1.049476
C 3.888656 0.265194 0.865528
C 3.588534 0.819731 -0.487830
C 2.954585 1.957942 -0.787033
C 2.686268 2.328838 -2.220222
C 2.444242 2.954772 0.215293
C 2.234853 -3.291619 -0.282582
O -0.463094 -3.412169 -0.549334
C -1.806307 -3.868112 -0.357663
C -2.367046 -0.973718 0.366273
N -2.465816 0.418766 0.737446
C -1.361496 0.851473 1.560400
C -2.127165 2.523240 0.081554
O -1.173539 2.225851 1.160744
O -2.943692 -0.981812 -0.965673
C -3.312199 0.394835 -1.315847
C -2.235561 1.148234 -0.554305
C -0.909953 0.963678 -1.377066
O -0.941709 1.873111 -2.484426
H 1.488764 0.771233 1.949026
H 3.606564 0.937621 1.674755
H 4.949856 0.041838 0.974470
H 3.903485 0.178544 -1.306827
H 3.076323 1.580867 -2.910851
H 1.610713 2.430638 -2.393310
H 3.139810 3.294847 -2.462055
H 1.353770 3.022685 0.160196
H 2.715534 2.718827 1.241549
H 2.835945 3.949615 -0.014367
H 2.048122 -3.446091 -1.347255
H 3.283252 -3.044530 -0.141981
H 2.029396 -4.242985 0.213984
H -2.048336 -3.914969 0.706108
H -2.521485 -3.232472 -0.878501
H -1.839064 -4.870279 -0.778180
H -2.984397 -1.612681 0.998561
H -1.599437 0.839823 2.626724
H -1.670272 3.277384 -0.550703
H -3.069947 2.878322 0.498872
H -3.263252 0.467528 -2.399138
H -4.320170 0.621475 -0.967559
H -0.040738 1.235277 -0.790764
H -0.791536 -0.059127 -1.723926
H -1.434130 1.471618 -3.208344

7*R*9*S*2''*R*-**11**-c12, ΔG = 1.1032 kcal/mol, population = 3.41 %

C 1.602314 -0.237544 -0.584541
C 1.060611 -1.531226 -0.680446
C -0.236453 -1.766477 -0.197700
C -0.977833 -0.720717 0.360185
C -0.388124 0.548093 0.454586
C 0.890893 0.809338 0.006103
O 2.849836 -0.095944 -1.111548
C 3.586248 1.125276 -0.865262
C 3.935342 1.348863 0.578780
C 4.720412 0.551242 1.308853

C 4.992918 0.843285 2.757368
C 5.363333 -0.697540 0.775847
C 1.881009 -2.634409 -1.288436
O -0.710104 -3.037868 -0.364964
C -1.628696 -3.564529 0.599129
C -2.406102 -0.643368 0.918341
N -2.613176 0.780183 1.037323
C -1.375093 1.517135 1.102226
C -3.085959 2.668750 -0.044775
O -1.695846 2.754218 0.429964
O -3.524133 -1.054032 0.091236
C -4.301508 0.142437 -0.263182
C -3.183215 1.171489 -0.289165
C -2.403185 0.908356 -1.615427
O -3.273697 1.418516 -2.639931
H 1.305763 1.801894 0.090235
H 4.476659 1.002660 -1.480481
H 3.024481 1.972684 -1.262977
H 3.498202 2.220633 1.053660
H 4.489629 1.751161 3.090001
H 4.658978 0.012227 3.386438
H 6.067157 0.955663 2.932537
H 4.900018 -1.580306 1.227848
H 5.272176 -0.794443 -0.303746
H 6.422496 -0.724896 1.045956
H 1.462221 -3.606397 -1.041804
H 1.914790 -2.547572 -2.378554
H 2.910905 -2.583240 -0.934643
H -1.280836 -3.362161 1.614288
H -2.630664 -3.160392 0.457681
H -1.647243 -4.639172 0.433151
H -2.500084 -1.152646 1.878370
H -1.080963 1.758508 2.126774
H -3.156919 3.278143 -0.939585
H -3.770435 3.020656 0.727615
H -4.762428 -0.050704 -1.226359
H -5.052520 0.346749 0.500376
H -1.452761 1.436701 -1.631946
H -2.224564 -0.153711 -1.766839
H -2.836545 1.290813 -3.489031

7*R*9*S*2''*R*-**11**-c8, ΔG = 1.2418 kcal/mol, population = 2.70 %

C 1.847133 -1.196375 0.794295
C 1.207330 -2.172563 0.023592
C -0.200783 -2.176505 -0.042142
C -0.936198 -1.179613 0.597988
C -0.249030 -0.234338 1.380435
C 1.120347 -0.241009 1.510549
O 3.219029 -1.232971 0.848524
C 3.910349 0.041270 0.728468
C 3.566554 0.717358 -0.557186
C 2.984080 1.908369 -0.726776
C 2.666834 2.411763 -2.108573
C 2.591912 2.846080 0.380059
C 1.986083 -3.204660 -0.742460
O -0.726885 -3.164238 -0.820907
C -2.078349 -3.582944 -0.603149
C -2.430413 -0.821437 0.597294
N -2.432105 0.497165 1.186046
C -1.249012 0.754473 1.971132
C -2.058512 2.661728 0.850974
O -1.029731 2.169258 1.774932
O -3.104750 -0.600405 -0.666995
C -3.429370 0.829062 -0.776704
C -2.269694 1.413765 0.009178
C -1.024905 1.313005 -0.944158
O -1.191351 2.257924 -2.008947
H 1.619568 0.500928 2.115683
H 3.711210 0.661085 1.601750
H 4.962260 -0.242843 0.755945
H 3.798627 0.129808 -1.441451
H 2.987937 1.710440 -2.878656
H 1.590527 2.573749 -2.219685
H 3.150913 3.375560 -2.292389
H 1.514942 3.035292 0.354296
H 2.845033 2.480111 1.371900
H 3.083592 3.813401 0.242746
H 3.052847 -3.018169 -0.657363
H 1.775054 -4.209994 -0.369779
H 1.708456 -3.196228 -1.798292
H -2.252634 -3.782821 0.456075
H -2.788918 -2.842360 -0.968262
H -2.194037 -4.505491 -1.166783
H -3.027381 -1.528378 1.174365
H -1.404479 0.583524 3.038972
H -1.633388 3.517283 0.333297
H -2.951992 2.960998 1.399481

H -3.429960 1.075725 -1.833532
H -4.400463 1.032685 -0.324706
H -0.094537 1.492396 -0.413794
H -0.972734 0.339749 -1.418140
H -0.944558 3.135330 -1.696374

7*R*9*S*2"*R*-**11**-c32, ΔG = 1.3379 kcal/mol, population = 2.30 %
C 1.793192 -1.001291 1.103719
C 1.302481 -2.005617 0.264048
C -0.084614 -2.064380 0.016273
C -0.937192 -1.120107 0.585646
C -0.400838 -0.152589 1.454959
C 0.946395 -0.088165 1.731246
O 3.146703 -0.908171 1.357623
C 3.913475 -0.260082 0.295956
C 3.490813 1.152602 0.070331
C 2.858561 1.638557 -1.003308
C 2.493586 3.095294 -1.087145
C 2.458252 0.830706 -2.206469
C 2.210677 -3.031828 -0.352088
O -0.472313 -3.054450 -0.838027
C -1.798087 -3.586244 -0.734118
C -2.441926 -0.853449 0.427297
N -2.589108 0.454093 1.021278
C -1.517265 0.772131 1.933598
C -2.302741 2.646081 0.732973
O -1.361397 2.197897 1.771149
O -2.989451 -0.657564 -0.900585
C -3.393219 0.747920 -1.028011
C -2.356431 1.394090 -0.125172
C -1.008100 1.380667 -0.929147
O -1.039906 2.448492 -1.883696
H 1.364190 0.675035 2.373163
H 4.940014 -0.307110 0.658290
H 3.845218 -0.859531 -0.610865
H 3.711679 1.833400 0.887683
H 2.974639 3.562391 -1.952496
H 1.415733 3.212203 -1.230030
H 2.791574 3.640443 -0.191481
H 3.017891 1.161313 -3.086962
H 2.615533 -0.238141 -2.085381
H 1.401692 0.995921 -2.428413
H 2.356165 -2.850526 -1.420671
H 3.184067 -3.023469 0.132871
H 1.776606 -4.027482 -0.259581
H -2.038843 -3.817061 0.305500
H -2.535714 -2.899826 -1.148327
H -1.792703 -4.505437 -1.314859
H -3.050405 -1.605022 0.931524
H -1.779290 0.578141 2.976508
H -1.857131 3.507696 0.246799
H -3.263963 2.900525 1.180476
H -3.325061 0.999860 -2.082942
H -4.414548 0.885679 -0.672469
H -0.163814 1.584298 -0.281735
H -0.846465 0.427602 -1.425029
H -1.506482 2.153390 -2.673188

7*R*9*S*2"*R*-**11**-c1, ΔG = 1.4282 kcal/mol, population = 1.97 %
C 1.798199 -0.975894 0.790845
C 1.225247 -1.994811 0.017105
C -0.175835 -2.085866 -0.063399
C -0.980839 -1.142774 0.576715
C -0.362154 -0.161550 1.369084
C 1.004849 -0.075671 1.509056
O 3.161606 -0.922309 0.804809
C 3.801334 0.326897 1.155273
C 3.314104 1.520115 0.378544
C 3.179748 1.595087 -0.947859
C 2.604827 2.821910 -1.598354
C 3.523816 0.473330 -1.886769
C 2.075475 -2.977886 -0.738773
O -0.633714 -3.095677 -0.858902
C -1.950186 -3.613174 -0.639978
C -2.491885 -0.876548 0.557850
N -2.581188 0.440165 1.144139
C -1.426418 0.768669 1.944749
C -2.322792 2.627287 0.799420
O -1.287160 2.192552 1.749655
O -3.164271 -0.695843 -0.716010
C -3.576876 0.708212 -0.822082
C -2.457528 1.363876 -0.032119
C -1.192534 1.342534 -0.965214
O -1.329129 2.395512 -1.928586
H 1.444567 0.703296 2.112998
H 3.684959 0.505676 2.226735

H 4.856709 0.122685 0.971364
H 3.017523 2.376122 0.975592
H 1.684706 2.572939 -2.136561
H 2.371943 3.597006 -0.867976
H 3.300065 3.233671 -2.336086
H 2.609006 -0.005351 -2.251443
H 4.050638 0.860295 -2.762663
H 4.131328 -0.297002 -1.417829
H 3.129780 -2.806424 -0.542823
H 1.824699 -4.002857 -0.458412
H 1.905185 -2.894809 -1.814885
H -2.001864 -4.537061 -1.211055
H -2.106170 -3.833841 0.418082
H -2.713923 -2.923030 -0.996674
H -3.054514 -1.618011 1.126140
H -1.586623 0.589144 3.010657
H -1.925689 3.481458 0.260820
H -3.236986 2.889570 1.332926
H -3.611133 0.947036 -1.881703
H -4.559025 0.853558 -0.371384
H -0.288555 1.561721 -0.409149
H -1.075120 0.383001 -1.461304
H -1.855170 2.077423 -2.670113

7*R*9*S*2''*R*-**11**-c35, ΔG = 1.6190 kcal/mol, population = 1.43 %

C 1.793475 -1.001962 1.099261
C 1.301523 -2.005507 0.259222
C -0.085223 -2.060775 0.008842
C -0.936934 -1.116221 0.579152
C -0.399397 -0.149070 1.448341
C 0.948123 -0.085980 1.724634
O 3.146143 -0.916698 1.360207
C 3.924636 -0.283888 0.299011
C 3.535602 1.139388 0.079636
C 2.906775 1.643144 -0.987574
C 2.590332 3.111346 -1.067227
C 2.469630 0.849196 -2.186967
C 2.207437 -3.036356 -0.352616
O -0.473085 -3.049429 -0.846838
C -1.800504 -3.578163 -0.748406
C -2.443267 -0.852450 0.424643
N -2.592251 0.450918 1.027351
C -1.517877 0.767164 1.936789
C -2.329164 2.641535 0.762333
O -1.371735 2.196474 1.782486
O -2.994116 -0.651995 -0.899542
C -3.399832 0.755616 -1.025244
C -2.370250 1.399361 -0.113847
C -1.027886 1.395017 -0.927577
O -1.144630 2.324301 -2.011538
H 1.366892 0.674932 2.368680
H 4.951006 -0.355563 0.657699
H 3.840226 -0.877829 -0.609891
H 3.783564 1.813354 0.894831
H 2.905765 3.644894 -0.170831
H 3.083203 3.565094 -1.932699
H 1.517029 3.267294 -1.206720
H 3.022200 1.170727 -3.075199
H 2.605102 -0.223306 -2.071971
H 1.413222 1.037344 -2.391818
H 2.360715 -2.854802 -1.420073
H 3.177861 -3.034620 0.138351
H 1.766592 -4.029321 -0.264003
H -1.795905 -4.494734 -1.333271
H -2.044207 -3.813171 0.289591
H -2.535243 -2.887889 -1.161247
H -3.047192 -1.608340 0.928072
H -1.774229 0.566832 2.979804
H -1.908828 3.533178 0.304163
H -3.292365 2.872560 1.217876
H -3.305896 1.021776 -2.072970
H -4.424180 0.886900 -0.675772
H -0.178002 1.644795 -0.298865
H -0.850300 0.429660 -1.386485
H -1.045387 3.221157 -1.673191

7*R*9*S*2''*R*-**11**-c10, ΔG = 1.6780 kcal/mol, population = 1.29 %

C 1.790585 -1.145470 0.446014
C 1.097375 -2.238375 -0.091916
C -0.308321 -2.262903 -0.021904
C -1.007804 -1.185824 0.525066
C -0.271157 -0.122537 1.072223
C 1.107830 -0.088982 1.062270
O 3.151809 -1.189995 0.361320
C 3.897856 0.043659 0.502900
C 3.591219 0.997999 -0.605339

C 3.205601 2.273564 -0.506757
C 2.923066 3.085719 -1.740446
C 3.002545 3.015914 0.784051
C 1.822245 -3.383845 -0.741963
O -0.880717 -3.365676 -0.580918
C -2.188699 -3.765891 -0.156463
C -2.505906 -0.860512 0.666091
N -2.481497 0.533595 1.026248
C -1.233080 0.935476 1.613892
C -2.214596 2.622593 0.284985
O -1.094432 2.301871 1.182427
O -3.364842 -0.860203 -0.501311
C -3.755461 0.529396 -0.781732
C -2.502459 1.251115 -0.299816
C -1.469784 0.996269 -1.466954
O -0.195225 1.611694 -1.403325
H 1.631794 0.743469 1.503090
H 3.736409 0.466422 1.494352
H 4.934028 -0.291274 0.452305
H 3.699092 0.567922 -1.597378
H 3.552030 3.980395 -1.769077
H 3.094182 2.511441 -2.650895
H 1.883069 3.423855 -1.737266
H 3.624415 3.915203 0.802099
H 1.963953 3.350616 0.863944
H 3.243989 2.431205 1.669084
H 1.595632 -4.324066 -0.234546
H 1.506816 -3.503562 -1.780881
H 2.896175 -3.223834 -0.720449
H -2.955510 -3.096238 -0.543570
H -2.337051 -4.762464 -0.564866
H -2.241900 -3.811096 0.933170
H -2.984849 -1.476333 1.428632
H -1.255651 0.928976 2.706677
H -1.845534 3.349975 -0.431220
H -3.048731 3.025981 0.860128
H -3.948992 0.589496 -1.849068
H -4.645926 0.797436 -0.213182
H -1.381741 -0.067704 -1.676604
H -1.953099 1.463634 -2.327020
H 0.334809 1.175507 -0.722576

7*R*9*S*2"*R*-**11**-c44, ΔG = 1.7401 kcal/mol, population = 1.16 %

C 1.782810 -0.699320 1.225424
C 1.472203 -1.625497 0.227544
C 0.120434 -1.795807 -0.142357
C -0.876279 -1.030475 0.461025
C -0.514643 -0.117550 1.470818
C 0.793328 0.051308 1.865534
O 3.091080 -0.550856 1.639261
C 3.752866 0.652750 1.142244
C 4.156849 0.514212 -0.288837
C 3.492012 0.967341 -1.356035
C 3.987828 0.683103 -2.746429
C 2.207649 1.743132 -1.300045
C 2.528763 -2.458816 -0.438940
O -0.081857 -2.706939 -1.137213
C -1.339255 -3.386758 -1.222610
C -2.402838 -0.972878 0.275595
N -2.763892 0.200228 1.033844
C -1.771758 0.555478 2.018539
C -2.846495 2.425676 1.061659
O -1.848314 1.996677 2.054032
O -2.962278 -0.685824 -1.029898
C -3.590551 0.642885 -0.982864
C -2.686362 1.312628 0.038428
C -1.358101 1.623042 -0.716657
O -1.683880 2.738990 -1.561247
H 1.065478 0.748491 2.647447
H 3.099379 1.509444 1.312258
H 4.626889 0.757759 1.782860
H 5.057011 -0.069071 -0.458282
H 4.916968 0.113500 -2.737492
H 3.239149 0.114793 -3.307578
H 4.153283 1.613007 -3.298875
H 2.297742 2.675881 -1.863958
H 1.411769 1.161445 -1.773546
H 1.888745 1.979096 -0.288177
H 3.493447 -2.298404 0.032698
H 2.272248 -3.518415 -0.386354
H 2.617456 -2.204405 -1.497483
H -2.125281 -2.729292 -1.591985
H -1.185481 -4.199666 -1.928123
H -1.618789 -3.800706 -0.251701
H -2.889431 -1.871833 0.655951
H -2.015532 0.181420 3.016072
H -2.547537 3.407820 0.710800

H -3.839129 2.452014 1.511989
H -3.530755 1.058632 -1.983295
H -4.625366 0.560969 -0.649722
H -0.564068 1.892436 -0.025872
H -1.036265 0.775928 -1.318330
H -0.889958 2.983737 -2.049445

7*R*9*S*2"*R*-**11**-c6, ΔG = 1.7526 kcal/mol, population = 1.14 %

C 1.774961 -0.947191 0.819681
C 1.217476 -1.951116 0.016371
C -0.182433 -2.046913 -0.085321
C -1.001502 -1.124250 0.566847
C -0.397590 -0.152001 1.382311
C 0.967515 -0.059204 1.537223
O 3.138694 -0.900813 0.876292
C 3.768138 0.363648 1.196617
C 3.316484 1.506165 0.330013
C 3.364879 1.528579 -1.004584
C 2.822637 2.694860 -1.781148
C 3.908004 0.401715 -1.836503
C 2.082443 -2.918267 -0.743419
O -0.623127 -3.042288 -0.908286
C -1.935993 -3.579101 -0.714981
C -2.519025 -0.887578 0.562773
N -2.629701 0.409301 1.186995
C -1.477635 0.739588 1.989663
C -2.429605 2.610831 0.912389
O -1.374292 2.171906 1.838638
O -3.207657 -0.686144 -0.697745
C -3.648563 0.714388 -0.767008
C -2.541959 1.369212 0.042535
C -1.294808 1.399578 -0.897797
O -1.596949 2.439116 -1.844610
H 1.395912 0.708410 2.164051
H 3.603666 0.597420 2.250563
H 4.829475 0.149109 1.071023
H 2.885377 2.356748 0.846709
H 2.411432 3.463718 -1.127016
H 3.603015 3.144496 -2.402484
H 2.033315 2.364195 -2.463825
H 4.546652 0.791219 -2.633555
H 4.472816 -0.320851 -1.251828
H 3.087869 -0.136933 -2.322047
H 1.838590 -3.948676 -0.476781
H 1.919877 -2.824880 -1.819767
H 3.133467 -2.740015 -0.536309
H -2.097434 -3.832786 0.334806
H -2.704487 -2.886840 -1.057039
H -1.973394 -4.486316 -1.313269
H -3.059527 -1.656232 1.116423
H -1.626252 0.523642 3.050532
H -2.058432 3.490656 0.397459
H -3.345258 2.831862 1.461612
H -3.666340 0.991392 -1.815979
H -4.633548 0.824861 -0.312639
H -0.386458 1.641867 -0.351777
H -1.160701 0.449807 -1.410454
H -0.854143 2.507729 -2.454346

7*R*9*S*2"*R*-**11**-c2, ΔG = 2.9016 kcal/mol, population = 0.16 %

C 1.758478 -0.905622 0.664117
C 1.175513 -1.957568 -0.056807
C -0.226144 -2.073159 -0.090682
C -1.029447 -1.118619 0.535938
C -0.402177 -0.088998 1.256344
C 0.969959 0.025694 1.351965
O 3.118738 -0.861504 0.662342
C 3.785626 0.330551 1.126508
C 3.426284 1.590343 0.386005
C 3.321437 1.734991 -0.937461
C 2.913595 3.049910 -1.539889
C 3.540785 0.620267 -1.922587
C 2.016737 -2.962329 -0.793332
O -0.687904 -3.125265 -0.823459
C -1.985961 -3.658813 -0.537962
C -2.552315 -0.902747 0.607939
N -2.644357 0.438220 1.125231
C -1.465427 0.839504 1.843244
C -2.467186 2.611098 0.645575
O -1.387772 2.252338 1.577622
O -3.336613 -0.816380 -0.607700
C -3.799272 0.571564 -0.755761
C -2.628439 1.303091 -0.109335
C -1.512956 1.246079 -1.225269
O -0.281155 1.908268 -1.001231
H 1.412387 0.829033 1.920827

H 3.596591 0.458619 2.195135
H 4.841544 0.082000 1.011508
H 3.229099 2.452032 1.015928
H 2.780936 3.820590 -0.780256
H 3.661726 3.393687 -2.260269
H 1.970096 2.938738 -2.081704
H 2.577511 0.233939 -2.272290
H 4.073539 0.990310 -2.801789
H 4.092553 -0.214922 -1.498299
H 1.798171 -3.975780 -0.450806
H 1.801566 -2.938539 -1.864169
H 3.073910 -2.761072 -0.648744
H -2.774991 -2.996753 -0.891941
H -2.038239 -4.604509 -1.071807
H -2.098689 -3.841897 0.532500
H -3.034356 -1.631976 1.260691
H -1.559482 0.704869 2.923884
H -2.101486 3.437213 0.043710
H -3.360507 2.896216 1.202359
H -3.929306 0.744920 -1.820377
H -4.739124 0.718870 -0.223820
H -1.352590 0.222219 -1.556231
H -1.968268 1.793161 -2.053028
H 0.222634 1.423484 -0.333284

Geometry data of conformers of structure 7*R*9*S*2"*S*-**11**

7*R*9*S*2"*S*-**11**-c28, ΔG = 0.0000 kcal/mol, population = 19.16 %
C 1.730543 0.346999 -0.435176
C 1.331169 1.695315 -0.493057
C -0.036842 2.001908 -0.532754
C -0.981126 0.965789 -0.523623
C -0.541068 -0.349221 -0.475892
C 0.798296 -0.692016 -0.439053
O 3.076137 0.146723 -0.380218
C 3.572200 -1.210752 -0.392267
C 5.060457 -1.152657 -0.424128
C 5.898799 -1.529080 0.546403
C 7.387054 -1.428934 0.352059
C 5.483051 -2.077153 1.882918
C 2.374443 2.777336 -0.503607
O -0.338922 3.332681 -0.522434
C -1.556092 3.793344 -1.115651
C -2.480190 0.991751 -0.426911
N -2.898061 -0.431125 -0.460804
C -1.706551 -1.302080 -0.426212
C -2.637339 -1.377921 1.684130
O -1.796264 -2.086909 0.770506
O -2.912104 1.522227 0.837454
C -4.028048 0.726815 1.240015
C -3.666727 -0.684055 0.783155
C -4.875102 -1.570015 0.478879
O -5.561816 -1.113768 -0.679585
H 1.095812 -1.728619 -0.396731
H 3.194282 -1.740727 0.482209
H 3.191818 -1.712610 -1.287630
H 5.480091 -0.761539 -1.346375
H 7.830699 -0.781666 1.114604
H 7.857346 -2.410787 0.462899
H 7.642854 -1.032680 -0.630556
H 5.840100 -3.105010 1.997870
H 5.946936 -1.494570 2.683831
H 4.406993 -2.072493 2.041354
H 3.191742 2.516286 -1.176179
H 1.943432 3.725494 -0.812886
H 2.811865 2.912163 0.490105
H -1.443222 4.869946 -1.218248
H -1.699519 3.349792 -2.102775
H -2.414546 3.580967 -0.478067
H -2.986658 1.535010 -1.225119
H -1.696512 -2.018084 -1.248607
H -2.063795 -0.638502 2.250684
H -3.074088 -2.103733 2.368742
H -4.134718 0.808458 2.320310
H -4.947355 1.066415 0.753430
H -4.537114 -2.604115 0.347333
H -5.579418 -1.546407 1.312123
H -4.855380 -0.878032 -1.304463

7*R*9*S*2"*S*-**11**-c29, ΔG = 0.1092 kcal/mol, population = 15.94 %
C 1.744305 0.346738 0.094208
C 1.350479 1.696704 0.128011
C -0.006224 2.019146 -0.031113
C -0.946994 1.000154 -0.231177
C -0.510961 -0.317525 -0.267427
C 0.815635 -0.675330 -0.117580

O 3.076430 0.118204 0.264678
C 3.554091 -1.244691 0.215636
C 5.021855 -1.223590 0.470671
C 5.991479 -1.512932 -0.402641
C 7.435715 -1.468647 0.015770
C 5.773735 -1.903483 -1.837703
C 2.365136 2.786102 0.337751
O -0.291146 3.347989 0.077727
C -1.470574 3.881739 -0.530072
C -2.447466 1.028056 -0.313956
N -2.850640 -0.379792 -0.549070
C -1.670305 -1.260218 -0.455059
C -2.863186 -1.545582 1.501632
O -1.903370 -2.159803 0.637622
O -3.036289 1.423689 0.936768
C -4.194213 0.603919 1.104206
C -3.771825 -0.754816 0.551737
C -4.925324 -1.594508 0.002490
O -5.458662 -1.014092 -1.180824
H 1.106662 -1.713875 -0.152826
H 3.042666 -1.821990 0.992265
H 3.305048 -1.680127 -0.752512
H 5.303910 -0.942588 1.481135
H 7.547427 -1.183208 1.061638
H 7.908665 -2.444647 -0.130089
H 7.992030 -0.756351 -0.600948
H 4.731661 -1.867655 -2.147100
H 6.345747 -1.240789 -2.493344
H 6.147155 -2.916366 -2.015858
H 2.112647 3.663983 -0.255114
H 2.394405 3.103019 1.384871
H 3.360764 2.442434 0.068352
H -1.360901 4.962292 -0.481493
H -1.542089 3.573071 -1.574694
H -2.367073 3.584824 0.014242
H -2.855330 1.656806 -1.105961
H -1.556463 -1.891312 -1.337076
H -2.375222 -0.872301 2.212428
H -3.379612 -2.336598 2.043834
H -4.441680 0.572947 2.163810
H -5.042408 1.003357 0.540195
H -4.566248 -2.611512 -0.191937
H -5.731922 -1.653672 0.735077
H -4.678288 -0.720345 -1.680713

7*R*9*S*2"*S*-**11**-c4, ΔG = 0.3502 kcal/mol, population = 10.61 %

C 2.065375 0.862350 0.594153
C 1.486962 2.124909 0.370547
C 0.139422 2.203303 -0.015150
C -0.603292 1.027994 -0.187302
C 0.014567 -0.196449 0.028989
C 1.337139 -0.314110 0.410252
O 3.364636 0.888405 1.005947
C 4.100047 -0.350963 1.111632
C 4.429181 -0.919860 -0.231278
C 4.237977 -2.170891 -0.658407
C 4.642381 -2.576827 -2.049046
C 3.622775 -3.273861 0.156494
C 2.311850 3.367795 0.555424
O -0.354603 3.468921 -0.143146
C -1.461200 3.720094 -1.013351
C -2.062046 0.803508 -0.469401
N -2.221265 -0.668913 -0.550931
C -0.955132 -1.329603 -0.178876
C -2.377512 -1.571578 1.623682
O -1.222069 -2.122358 0.985445
O -2.881778 1.248934 0.624924
C -3.922111 0.279695 0.759013
C -3.235049 -1.050726 0.462822
C -4.166232 -2.120563 -0.108090
O -4.593121 -1.775852 -1.419775
H 1.773535 -1.286999 0.563462
H 5.009184 -0.052778 1.634976
H 3.561553 -1.046507 1.755368
H 4.887485 -0.205799 -0.910267
H 3.776216 -2.947768 -2.605789
H 5.079922 -1.746387 -2.603021
H 5.368956 -3.394488 -2.019231
H 2.750045 -3.682770 -0.361615
H 4.332025 -4.098757 0.270592
H 3.316003 -2.963883 1.153190
H 2.374806 3.647359 1.611657
H 3.331114 3.206107 0.206704
H 1.873568 4.204044 0.016841
H -1.298662 3.262515 -1.991129
H -2.396148 3.363607 -0.580663
H -1.506503 4.800667 -1.125373

H -2.431468 1.257373 -1.389341
H -0.618128 -2.031635 -0.942373
H -2.101144 -0.752699 2.294383
H -2.853564 -2.366520 2.196082
H -4.315284 0.337136 1.772548
H -4.727033 0.466304 0.041672
H -3.645004 -3.084534 -0.110390
H -5.056478 -2.217528 0.515495
H -3.795914 -1.423094 -1.850151

7*R*9*S*2"*S*-**11**-c1, ΔG = 0.4273 kcal/mol, population = 9.31 %

C 1.889529 0.343619 0.709993
C 1.511528 1.638891 0.315042
C 0.202895 1.861985 -0.142733
C -0.699248 0.793315 -0.218618
C -0.275512 -0.472875 0.164443
C 1.002325 -0.730652 0.621130
O 3.159621 0.227074 1.186889
C 3.736214 -1.091798 1.341376
C 3.904583 -1.839356 0.049136
C 4.682290 -1.447926 -0.964624
C 4.771896 -2.251058 -2.231326
C 5.498062 -0.186507 -0.948121
C 2.500435 2.768595 0.387995
O -0.088519 3.160927 -0.440359
C -1.127300 3.465953 -1.374692
C -2.164934 0.747108 -0.546699
N -2.536672 -0.686655 -0.468684
C -1.395011 -1.472228 0.039273
C -2.894986 -1.286792 1.784129
O -1.814422 -2.071813 1.272501
O -2.946631 1.432427 0.447211
C -4.122843 0.644252 0.635601
C -3.628863 -0.795979 0.529646
C -4.686274 -1.785876 0.040077
O -5.016784 -1.546402 -1.321957
H 1.286972 -1.730038 0.911936
H 4.694644 -0.885288 1.815735
H 3.137300 -1.669275 2.047955
H 3.335883 -2.756735 -0.057651
H 5.804912 -2.558891 -2.419864
H 4.145819 -3.142579 -2.192534
H 4.463720 -1.648672 -3.091572
H 5.524915 0.289200 0.029657
H 6.522131 -0.392235 -1.271457
H 5.083609 0.537455 -1.656848
H 2.270277 3.528458 -0.355729
H 2.478552 3.253466 1.369060
H 3.512299 2.400253 0.230574
H -1.012930 2.879370 -2.288278
H -2.113479 3.300172 -0.940589
H -1.010301 4.521345 -1.608429
H -2.432708 1.135494 -1.529653
H -1.140735 -2.302309 -0.620713
H -2.521383 -0.438645 2.365394
H -3.500724 -1.927867 2.423152
H -4.539732 0.878737 1.613475
H -4.864380 0.854904 -0.140811
H -4.310055 -2.806348 0.174487
H -5.601198 -1.682101 0.625932
H -4.163739 -1.366017 -1.751841

7*R*9*S*2"*S*-**11**-c41, ΔG = 0.4706 kcal/mol, population = 8.65 %

C 1.723078 0.344865 -0.439711
C 1.320313 1.692406 -0.501715
C -0.048114 1.995716 -0.542071
C -0.992328 0.958736 -0.526250
C -0.546919 -0.353711 -0.469812
C 0.792946 -0.695727 -0.436121
O 3.069030 0.147405 -0.386858
C 3.566280 -1.210172 -0.382668
C 5.054635 -1.154363 -0.419321
C 5.892479 -1.478178 0.570555
C 7.380904 -1.390843 0.371936
C 5.475125 -1.948850 1.935688
C 2.362212 2.775588 -0.523200
O -0.355056 3.326286 -0.541642
C -1.537931 3.784083 -1.203406
C -2.502205 0.993199 -0.489403
N -2.908497 -0.429738 -0.456498
C -1.710544 -1.290898 -0.360920
C -3.122540 -1.953815 1.314535
O -1.747562 -1.923361 0.927512
O -3.070109 1.611424 0.677308
C -3.312415 0.579799 1.636436
C -3.690783 -0.639427 0.786807

C -5.183906 -0.690040 0.460450
O -5.429989 -1.525905 -0.663117
H 1.092530 -1.731086 -0.384023
H 3.190261 -1.727897 0.499997
H 3.183886 -1.724185 -1.269975
H 5.474919 -0.812389 -1.360532
H 7.848553 -2.367010 0.532847
H 7.637864 -1.045835 -0.629503
H 7.826075 -0.706817 1.100681
H 5.849160 -2.960615 2.118439
H 5.922358 -1.308254 2.701139
H 4.397955 -1.952308 2.086182
H 2.810947 2.909612 0.465487
H 3.172176 2.516227 -1.205393
H 1.926285 3.723324 -0.826674
H -1.411889 4.857797 -1.321344
H -1.636078 3.322542 -2.187852
H -2.428219 3.590743 -0.605227
H -2.948729 1.503743 -1.338881
H -1.730788 -2.065040 -1.133216
H -3.171057 -2.030952 2.399410
H -3.638514 -2.806053 0.861979
H -2.412957 0.373626 2.224113
H -4.110244 0.917213 2.296550
H -5.741880 -1.097260 1.305340
H -5.544596 0.327703 0.273007
H -4.714577 -1.312681 -1.285978

7*R*9*S*2"*S*-**11**-c27, ΔG = 0.4964 kcal/mol, population = 8.28 %

C 1.731602 0.193733 0.117363
C 1.381235 1.554121 0.027646
C 0.036869 1.903155 -0.166284
C -0.935233 0.897347 -0.271887
C -0.540793 -0.429558 -0.179411
C 0.774899 -0.815890 0.002350
O 3.057488 -0.047748 0.308938
C 3.501148 -1.420018 0.403454
C 4.968100 -1.408493 0.662703
C 5.932201 -1.826173 -0.163422
C 7.375666 -1.771596 0.256558
C 5.708698 -2.379887 -1.542875
C 2.453632 2.601549 0.139532
O -0.223715 3.243324 -0.184174
C -1.316264 3.747536 -0.957794
C -2.436761 0.979452 -0.416728
N -2.887058 -0.429476 -0.453646
C -1.737726 -1.329327 -0.220602
C -3.366032 -1.961523 1.257455
O -1.952689 -1.966528 1.048057
O -3.123961 1.604100 0.680958
C -3.521751 0.571731 1.585672
C -3.825794 -0.626588 0.678056
C -5.266582 -0.628504 0.166141
O -5.393340 -1.444380 -0.991312
H 1.034143 -1.861050 0.071475
H 2.972788 -1.900368 1.233035
H 3.243513 -1.946861 -0.515728
H 5.254293 -1.017440 1.634680
H 7.491633 -1.366666 1.261744
H 7.821848 -2.770477 0.231090
H 7.953670 -1.153222 -0.436788
H 6.304137 -1.819006 -2.268863
H 6.050908 -3.417836 -1.594548
H 4.669606 -2.350061 -1.862855
H 2.085446 3.567433 -0.194859
H 2.793287 2.707165 1.174078
H 3.325817 2.322942 -0.452411
H -1.150064 4.819332 -1.036652
H -1.320015 3.309152 -1.957620
H -2.270249 3.567449 -0.462696
H -2.760801 1.512149 -1.307183
H -1.687845 -2.098872 -0.996148
H -3.553951 -2.045909 2.326434
H -3.843218 -2.795104 0.733227
H -2.713564 0.329507 2.282139
H -4.387265 0.929600 2.141632
H -5.937972 -1.027462 0.928368
H -5.570798 0.401857 -0.050239
H -4.600659 -1.244779 -1.517674

7*R*9*S*2"*S*-**11**-c10, ΔG = 0.6752 kcal/mol, population = 6.12 %

C 2.056199 0.856107 0.646521
C 1.478176 2.112825 0.389399
C 0.137838 2.179346 -0.021259
C -0.601809 0.999636 -0.178581
C 0.013311 -0.216680 0.083187

C 1.331522 -0.325147 0.482873
O 3.350172 0.893008 1.074311
C 4.107385 -0.336369 1.145133
C 4.422263 -0.870132 -0.215283
C 4.238908 -2.112323 -0.670511
C 4.620278 -2.476402 -2.079229
C 3.654858 -3.244396 0.126903
C 2.301261 3.359534 0.554608
O -0.359351 3.439883 -0.191562
C -1.382307 3.676377 -1.163310
C -2.049783 0.776393 -0.546734
N -2.219682 -0.693738 -0.523240
C -0.973945 -1.335116 -0.051549
C -2.668158 -2.185721 1.231349
O -1.262888 -1.928175 1.223742
O -3.006692 1.317197 0.379430
C -3.342257 0.283384 1.307614
C -3.271044 -1.003786 0.476964
C -4.588392 -1.319990 -0.232072
O -4.379014 -2.210606 -1.320485
H 1.764978 -1.292570 0.674909
H 5.019377 -0.032916 1.660256
H 3.591481 -1.053978 1.783108
H 4.857350 -0.133176 -0.885030
H 5.032816 -1.625206 -2.620674
H 5.359681 -3.282952 -2.085387
H 3.748296 -2.846853 -2.627172
H 3.382393 -2.970084 1.143712
H 2.765821 -3.638750 -0.374636
H 4.369703 -4.070016 0.188201
H 2.373490 3.648922 1.607481
H 3.318098 3.196777 0.198654
H 1.855765 4.188865 0.011493
H -1.404198 4.753598 -1.310761
H -1.140044 3.188062 -2.109209
H -2.356216 3.343127 -0.805222
H -2.320030 1.182644 -1.518313
H -0.660819 -2.123050 -0.742451
H -3.000268 -2.240958 2.266622
H -2.897052 -3.126335 0.721257
H -2.623812 0.249958 2.132073
H -4.336086 0.495690 1.699381
H -5.284132 -1.798830 0.458921
H -5.041752 -0.384322 -0.578330
H -3.563591 -1.890736 -1.742324

7*R*9*S*2"*S*-**11**-c7, ΔG = 0.7398 kcal/mol, population = 5.49 %

C 1.885216 0.350754 0.729497
C 1.504270 1.640358 0.317763
C 0.198658 1.852132 -0.152457
C -0.701011 0.780052 -0.219192
C -0.275581 -0.476988 0.189756
C 1.001511 -0.726876 0.653961
O 3.154458 0.245044 1.210780
C 3.742502 -1.069152 1.363050
C 3.903765 -1.818123 0.070690
C 4.661115 -1.418488 -0.955185
C 4.742070 -2.222590 -2.221852
C 5.459729 -0.146030 -0.953345
C 2.493696 2.770246 0.378810
O -0.098653 3.145223 -0.473966
C -1.065138 3.424459 -1.491073
C -2.155456 0.725490 -0.623059
N -2.535640 -0.695867 -0.462059
C -1.409629 -1.453069 0.124198
C -3.245068 -1.920979 1.409145
O -1.817638 -1.864219 1.438102
O -3.049639 1.487992 0.206118
C -3.555605 0.611973 1.215293
C -3.649212 -0.750299 0.516959
C -4.977446 -0.948672 -0.213297
O -4.871843 -1.974013 -1.192662
H 1.286681 -1.719453 0.966568
H 4.703723 -0.854177 1.827994
H 3.154749 -1.649695 2.076476
H 3.346021 -2.743319 -0.025696
H 4.128822 -3.122432 -2.172008
H 4.412149 -1.625807 -3.077952
H 5.776087 -2.516564 -2.426270
H 5.498764 0.329483 0.024100
H 6.480089 -0.337562 -1.296426
H 5.021861 0.572529 -1.653473
H 2.238042 3.543243 -0.342177
H 2.505585 3.234603 1.369834
H 3.500436 2.406310 0.181119
H -0.928574 4.472825 -1.745692
H -0.885157 2.812443 -2.376981

H -2.081178 3.272599 -1.127284
H -2.339143 1.062626 -1.640103
H -1.193679 -2.344644 -0.471408
H -3.612332 -1.822218 2.429193
H -3.590999 -2.867784 0.983691
H -2.870743 0.559030 2.066716
H -4.517815 1.001683 1.544975
H -5.756008 -1.243487 0.492271
H -5.276645 -0.000754 -0.674699
H -4.008585 -1.821937 -1.612845

7*R*9*S*2"*S*-**11**-c6, ΔG = 0.7486 kcal/mol, population = 5.41 %

C 2.172417 1.108315 -0.323407
C 1.540399 2.242868 0.211644
C 0.136648 2.296927 0.244858
C -0.615916 1.218219 -0.233328
C 0.052869 0.119171 -0.759489
C 1.428613 0.038572 -0.828330
O 3.536854 1.142239 -0.347457
C 4.267665 -0.072726 -0.631288
C 4.131851 -1.068701 0.474925
C 3.783737 -2.354829 0.381773
C 3.690963 -3.214402 1.612517
C 3.447709 -3.066526 -0.898571
C 2.334627 3.401788 0.747772
O -0.363947 3.431316 0.810343
C -1.711467 3.832171 0.550564
C -2.089908 0.927115 -0.192961
N -2.248026 -0.378498 -0.878934
C -0.921129 -0.945659 -1.187890
C -1.737133 -2.127280 0.621059
O -0.816940 -2.184695 -0.472248
O -2.551128 0.742428 1.156899
C -3.493967 -0.329288 1.108633
C -2.908524 -1.295264 0.082379
C -3.951790 -2.146433 -0.642122
O -4.753424 -1.345871 -1.501098
H 1.899312 -0.835329 -1.246880
H 3.973660 -0.465807 -1.604526
H 5.297700 0.275473 -0.713598
H 4.349945 -0.662866 1.459147
H 2.682347 -3.626948 1.713714
H 4.373074 -4.067155 1.543646
H 3.928234 -2.654437 2.516998
H 3.541153 -2.442882 -1.785137
H 4.103497 -3.931494 -1.032366
H 2.425000 -3.453426 -0.854742
H 2.101143 3.580587 1.799720
H 3.400563 3.216209 0.654779
H 2.089479 4.321135 0.211307
H -1.905013 3.869049 -0.523194
H -2.425663 3.170048 1.040217
H -1.803387 4.832525 0.966169
H -2.723155 1.669569 -0.679296
H -0.820490 -1.205593 -2.242112
H -1.284946 -1.636692 1.487983
H -2.010089 -3.148927 0.882121
H -3.570857 -0.763065 2.104048
H -4.476816 0.026946 0.785720
H -3.440528 -2.932407 -1.209492
H -4.615684 -2.625962 0.079154
H -4.124628 -0.733126 -1.918746

7*R*9*S*2"*S*-**11**-c12, ΔG = 0.9469 kcal/mol, population = 3.87 %

C 2.152774 1.050579 -0.347312
C 1.549757 2.213323 0.160557
C 0.148074 2.303428 0.192872
C -0.633672 1.233120 -0.258072
C 0.008774 0.105526 -0.754808
C 1.382037 -0.012267 -0.824815
O 3.517736 1.048343 -0.372094
C 4.216105 -0.198714 -0.593590
C 4.045107 -1.135429 0.558401
C 3.651311 -2.411315 0.526694
C 3.525411 -3.205702 1.797658
C 3.293331 -3.172652 -0.718546
C 2.374055 3.365579 0.664935
O -0.324545 3.463795 0.730881
C -1.634601 3.931258 0.398686
C -2.128121 1.010739 -0.249208
N -2.311049 -0.330814 -0.846003
C -0.993609 -0.952834 -1.097446
C -2.220417 -2.484632 0.081037
O -0.882458 -2.078218 -0.212392
O -2.727954 0.964801 1.057913
C -2.775780 -0.404046 1.465175

C -3.001529 -1.175914 0.159516
C -4.481951 -1.308952 -0.198436
O -4.646562 -1.601737 -1.580009
H 1.830552 -0.910294 -1.215374
H 3.917157 -0.629811 -1.549030
H 5.255853 0.116636 -0.685004
H 4.275286 -0.690212 1.522650
H 4.177414 -4.084072 1.773655
H 3.779720 -2.610591 2.674663
H 2.502746 -3.577462 1.914643
H 3.412933 -2.597407 -1.634190
H 3.917074 -4.066735 -0.806141
H 2.256846 -3.518914 -0.660907
H 2.129539 4.282429 0.124446
H 2.168759 3.559191 1.720324
H 3.434578 3.162261 0.549910
H -1.685328 4.949691 0.776080
H -1.780627 3.941121 -0.683163
H -2.406385 3.330023 0.878534
H -2.687446 1.760843 -0.802327
H -0.927563 -1.310323 -2.128921
H -2.213647 -3.030435 1.022904
H -2.618659 -3.124537 -0.712137
H -1.833699 -0.706343 1.931932
H -3.588453 -0.511050 2.182515
H -4.936038 -2.122878 0.369230
H -4.999147 -0.379528 0.065517
H -3.998298 -1.032630 -2.028356

7*R*9*S*2"*S*-**11**-c2, ΔG = 1.2569 kcal/mol, population = 2.29 %

C 2.001151 0.584894 -0.747498
C 1.560698 1.828920 -0.266107
C 0.182643 2.065865 -0.131487
C -0.734391 1.058650 -0.454016
C -0.252929 -0.157022 -0.925533
C 1.092134 -0.419202 -1.091785
O 3.349711 0.444420 -0.881729
C 3.899492 -0.869806 -1.134576
C 3.673793 -1.852207 -0.020274
C 4.146431 -1.722957 1.222837
C 3.847511 -2.749421 2.278673
C 4.974986 -0.556966 1.681839
C 2.536175 2.910706 0.107152
O -0.125545 3.295243 0.369321
C -1.431588 3.844386 0.177787
C -2.224067 0.965774 -0.277553
N -2.599935 -0.360506 -0.825116
C -1.383786 -1.118471 -1.178901
C -2.174618 -2.026336 0.790344
O -1.370867 -2.296643 -0.361030
O -2.587863 0.956538 1.114290
C -3.654247 0.014396 1.237527
C -3.280335 -1.101112 0.265875
C -4.476887 -1.871286 -0.293030
O -5.248334 -1.051854 -1.162153
H 1.417878 -1.377123 -1.466259
H 3.502273 -1.254725 -2.075722
H 4.959082 -0.666890 -1.284342
H 3.068650 -2.721024 -0.255591
H 3.232941 -3.562607 1.892298
H 4.774216 -3.172091 2.678658
H 3.323401 -2.290973 3.123052
H 4.404687 0.053282 2.389407
H 5.862550 -0.908760 2.214866
H 5.287333 0.090467 0.865411
H 3.556578 2.592564 -0.084612
H 2.338238 3.824221 -0.457395
H 2.445317 3.169082 1.164902
H -1.365558 4.877101 0.510981
H -1.709782 3.823310 -0.877648
H -2.175888 3.317961 0.775220
H -2.800395 1.741390 -0.782741
H -1.406907 -1.475414 -2.209010
H -1.590062 -1.522578 1.565691
H -2.543734 -2.977597 1.171303
H -3.697049 -0.321127 2.272182
H -4.610786 0.465811 0.957511
H -4.115024 -2.761841 -0.819232
H -5.128159 -2.197996 0.519367
H -4.589355 -0.561110 -1.681863

7*R*9*S*2"*S*-**11**-c8, ΔG = 1.5556 kcal/mol, population = 1.38 %

C 1.994803 0.585742 -0.762885
C 1.554539 1.824945 -0.268543
C 0.177100 2.058127 -0.124063
C -0.741682 1.051818 -0.446759

C -0.258219 -0.159335 -0.927078
C 1.085978 -0.418255 -1.107260
O 3.342709 0.449412 -0.907497
C 3.895412 -0.864991 -1.153156
C 3.676200 -1.838977 -0.030165
C 4.148705 -1.695403 1.211455
C 3.855587 -2.712949 2.277482
C 4.971167 -0.520609 1.659021
C 2.530422 2.907673 0.101292
O -0.131056 3.284882 0.385146
C -1.415053 3.865105 0.140803
C -2.244879 0.987170 -0.309754
N -2.609839 -0.355196 -0.813046
C -1.386353 -1.124045 -1.126533
C -2.661672 -2.457287 0.227317
O -1.316991 -2.207083 -0.186292
O -2.739140 1.069764 1.038935
C -2.895467 -0.264244 1.526671
C -3.299497 -1.072167 0.287617
C -4.809416 -1.066913 0.046883
O -5.110763 -1.409704 -1.299730
H 1.412053 -1.374665 -1.484968
H 3.496201 -1.258291 -2.089943
H 4.953852 -0.660056 -1.308375
H 3.075368 -2.712975 -0.257169
H 3.245916 -3.533607 1.899283
H 4.784636 -3.126044 2.682003
H 3.328559 -2.248984 3.117036
H 5.280718 0.120140 0.836309
H 4.397406 0.093913 2.360066
H 5.860115 -0.862521 2.196138
H 2.320407 3.825151 -0.452200
H 2.453152 3.156129 1.162476
H 3.549504 2.595810 -0.107040
H -1.656036 3.839795 -0.923680
H -2.193775 3.366095 0.716828
H -1.334038 4.899773 0.464989
H -2.768807 1.763320 -0.861658
H -1.441671 -1.539811 -2.136612
H -2.635123 -2.954167 1.195613
H -3.183114 -3.090633 -0.496745
H -1.956251 -0.641556 1.941767
H -3.657295 -0.248659 2.305055
H -5.297866 -1.798784 0.692295
H -5.207512 -0.075990 0.293132
H -4.447353 -0.932733 -1.826567

7*R*9*S*2"*S*-**11**-c58, ΔG = 1.7049 kcal/mol, population = 1.07 %

C 2.246132 0.674025 -0.995274
C 1.826692 1.867548 -0.398673
C 0.452220 2.061064 -0.155160
C -0.458631 1.061316 -0.504773
C 0.003528 -0.097053 -1.125579
C 1.339712 -0.306619 -1.391776
O 3.591036 0.446562 -1.203326
C 4.283622 -0.081350 -0.023012
C 3.803581 -1.444891 0.347814
C 2.874344 -1.736151 1.265187
C 2.412585 -3.151899 1.469412
C 2.174621 -0.724485 2.128121
C 2.805558 2.939137 -0.011437
O 0.152122 3.232402 0.471875
C -1.169026 3.774435 0.393924
C -1.927625 0.911678 -0.217905
N -2.322162 -0.368938 -0.851667
C -1.126729 -1.059443 -1.374343
C -1.732817 -2.164896 0.560112
O -1.022332 -2.306075 -0.671968
O -2.164422 0.767294 1.192186
C -3.199174 -0.209364 1.319891
C -2.894291 -1.222209 0.219325
C -4.122614 -1.964583 -0.307626
O -4.979681 -1.085410 -1.024368
H 1.698282 -1.216984 -1.850516
H 5.329132 -0.100467 -0.324653
H 4.174544 0.633247 0.793215
H 4.208419 -2.258255 -0.247208
H 2.556925 -3.463385 2.508201
H 1.340664 -3.232013 1.263233
H 2.940758 -3.849791 0.819774
H 2.201385 -1.034501 3.176153
H 2.594449 0.275126 2.052934
H 1.120346 -0.661529 1.842277
H 3.784826 2.735881 -0.437813
H 2.462332 3.914269 -0.358622
H 2.908903 3.010816 1.074787
H -1.095632 4.781493 0.796674

H -1.506476 3.823986 -0.643068
H -1.871627 3.195517 0.993427
H -2.559580 1.713953 -0.599330
H -1.230513 -1.321924 -2.427443
H -1.096045 -1.721849 1.331462
H -2.052045 -3.156388 0.878128
H -3.142857 -0.638609 2.318573
H -4.184159 0.241752 1.167840
H -3.793404 -2.791994 -0.946381
H -4.696282 -2.383460 0.520704
H -4.377691 -0.536673 -1.554919

7*R*9*S*2"*S*-**11**-c80, ΔG = 2.0124 kcal/mol, population = 0.64 %

C 2.084412 0.130657 -1.349784
C 1.881143 1.374160 -0.745671
C 0.573078 1.741058 -0.367990
C -0.486711 0.855621 -0.581837
C -0.234467 -0.364408 -1.205341
C 1.027217 -0.740302 -1.611537
O 3.362039 -0.255724 -1.698491
C 3.942578 -1.241246 -0.783129
C 4.221030 -0.654528 0.561478
C 3.425651 -0.707533 1.635552
C 3.794835 0.025072 2.895493
C 2.110575 -1.431129 1.700628
C 3.016401 2.323031 -0.490604
O 0.488523 2.954501 0.245850
C -0.745011 3.678365 0.249562
C -1.942715 0.931083 -0.179315
N -2.536283 -0.330579 -0.673415
C -1.481910 -1.191468 -1.251017
C -2.630994 -2.486043 0.248334
O -1.367433 -2.343862 -0.402706
O -2.172280 0.967249 1.239445
C -2.378331 -0.377212 1.679353
C -3.088503 -1.052110 0.499834
C -4.607102 -0.880873 0.550423
O -5.185343 -1.095777 -0.730421
H 1.213851 -1.695090 -2.084944
H 3.277608 -2.104429 -0.731946
H 4.863079 -1.546078 -1.277277
H 5.130050 -0.064719 0.629120
H 3.846404 -0.661197 3.746035
H 4.752446 0.537170 2.802337
H 3.028539 0.768216 3.138808
H 1.903836 -2.035805 0.822176
H 2.074482 -2.074416 2.584107
H 1.295624 -0.707704 1.801893
H 3.933677 1.946408 -0.933269
H 2.794610 3.308460 -0.904047
H 3.180385 2.455770 0.581087
H -1.161499 3.738328 -0.757632
H -1.467008 3.230922 0.932048
H -0.494999 4.678690 0.594419
H -2.467211 1.791519 -0.586248
H -1.761444 -1.520538 -2.255708
H -2.480255 -3.050021 1.167212
H -3.345395 -3.011722 -0.392417
H -1.424488 -0.870494 1.887566
H -2.976928 -0.342771 2.588550
H -5.045640 -1.605737 1.238207
H -4.842095 0.124140 0.918835
H -4.580668 -0.651152 -1.348317

7*R*9*S*2"*S*-**11**-c62, ΔG = 2.1235 kcal/mol, population = 0.53 %

C 2.252399 0.709073 -0.978380
C 1.816804 1.892471 -0.371993
C 0.438632 2.072757 -0.143304
C -0.461390 1.067797 -0.507677
C 0.018623 -0.080044 -1.133052
C 1.358081 -0.273717 -1.396045
O 3.601820 0.489904 -1.163633
C 4.270426 -0.060319 0.022430
C 3.782399 -1.430685 0.355321
C 2.817766 -1.737154 1.230606
C 2.332554 -3.151912 1.377688
C 2.090469 -0.741830 2.089209
C 2.783686 2.966969 0.036852
O 0.117971 3.236812 0.489251
C -1.182246 3.810221 0.323727
C -1.947005 0.932411 -0.261536
N -2.306867 -0.380387 -0.840629
C -1.089774 -1.068755 -1.322848
C -2.187757 -2.558028 0.026202
O -0.897943 -2.219439 -0.485611
O -2.322037 0.889446 1.125142

C -2.383192 -0.484403 1.514832
C -2.867344 -1.210700 0.254277
C -4.391835 -1.252030 0.149838
O -4.804324 -1.507342 -1.186509
H 1.729312 -1.179125 -1.853983
H 5.321964 -0.072063 -0.257382
H 4.142085 0.639142 0.848948
H 4.205462 -2.233077 -0.241944
H 1.276669 -3.210850 1.094749
H 2.896683 -3.842746 0.750941
H 2.403104 -3.484837 2.417399
H 1.044580 -0.683261 1.774185
H 2.090555 -1.067676 3.132834
H 2.505919 0.261118 2.039800
H 2.876713 3.026571 1.124724
H 3.768454 2.776161 -0.382596
H 2.435608 3.943332 -0.301847
H -1.457924 3.845629 -0.731919
H -1.933732 3.263486 0.892907
H -1.106298 4.823920 0.709433
H -2.540384 1.728191 -0.704168
H -1.219552 -1.402455 -2.356006
H -2.052143 -3.124730 0.945744
H -2.751208 -3.157067 -0.695448
H -1.396881 -0.850562 1.814627
H -3.070535 -0.562615 2.356010
H -4.791868 -2.049628 0.777969
H -4.800104 -0.299800 0.506811
H -4.212128 -0.964306 -1.733510

7*R*9*S*2"*S*-**11**-c91, ΔG = 2.1693 kcal/mol, population = 0.49 %

C 1.950953 0.010058 1.277126
C 1.792896 1.238113 0.629881
C 0.603067 1.480621 -0.087559
C -0.397178 0.506365 -0.121781
C -0.200103 -0.697113 0.553029
C 0.957386 -0.968926 1.248721
O 3.122268 -0.253030 1.956881
C 4.025466 -1.161047 1.247148
C 4.596882 -0.526491 0.022685
C 4.137457 -0.647287 -1.227519
C 4.756273 0.140515 -2.348506
C 2.979123 -1.509916 -1.641960
C 2.860887 2.292744 0.661776
O 0.545872 2.708168 -0.674711
C -0.339457 2.941422 -1.773019
C -1.790901 0.528986 -0.688323
N -2.344249 -0.819899 -0.421293
C -1.402908 -1.592130 0.413601
C -3.126259 -0.934303 1.802601
O -2.081242 -1.896558 1.638796
O -2.622805 1.465373 0.016313
C -3.904045 0.844329 0.132834
C -3.584723 -0.629337 0.370329
C -4.669266 -1.590333 -0.116778
O -4.755454 -1.585823 -1.535978
H 1.106557 -1.910424 1.760877
H 4.802845 -1.379137 1.976941
H 3.490696 -2.086079 1.028198
H 5.420425 0.158517 0.199410
H 5.130478 -0.524343 -3.132788
H 4.005576 0.785225 -2.816722
H 5.578373 0.765955 -2.000776
H 3.255373 -2.128538 -2.500228
H 2.615140 -2.159498 -0.850403
H 2.144027 -0.878563 -1.961158
H 3.244547 2.483465 -0.342720
H 2.463532 3.237857 1.037077
H 3.686203 1.979223 1.293564
H -0.064679 3.916440 -2.167387
H -0.199690 2.185888 -2.548376
H -1.379708 2.961871 -1.447844
H -1.851627 0.739574 -1.756143
H -1.153843 -2.555425 -0.032706
H -2.751737 -0.027869 2.287280
H -3.900713 -1.384671 2.422086
H -4.432305 1.303251 0.966623
H -4.485244 0.970152 -0.785483
H -4.448294 -2.597015 0.255615
H -5.642942 -1.290350 0.274362
H -3.831576 -1.581056 -1.837561

7*R*9*S*2"*S*-**11**-c30, ΔG = 2.3155 kcal/mol, population = 0.38 %

C 2.094908 1.126850 -0.224210
C 1.450605 2.242765 0.341338
C 0.053791 2.339236 0.256602

C -0.675741 1.323348 -0.374773
C 0.006450 0.261583 -0.947270
C 1.381339 0.138486 -0.901285
O 3.451446 1.109437 -0.079915
C 4.166453 -0.121979 -0.341094
C 3.829765 -1.171533 0.668725
C 3.402806 -2.417590 0.446493
C 3.080308 -3.332783 1.595754
C 3.184605 -3.026950 -0.909767
C 2.270168 3.294594 1.035689
O -0.510138 3.420929 0.872125
C -1.699686 3.992371 0.317466
C -2.169906 1.069348 -0.449033
N -2.288269 -0.304736 -1.005268
C -0.972986 -0.720173 -1.545127
C -1.414489 -2.232814 0.115202
O -0.796396 -2.070721 -1.168184
O -2.821509 1.029863 0.815061
C -2.521752 -0.253574 1.370951
C -2.559085 -1.202975 0.163098
C -3.947072 -1.818890 -0.010646
O -4.092907 -2.416654 -1.291385
H 1.864481 -0.715411 -1.345502
H 3.996373 -0.441545 -1.369076
H 5.211016 0.178401 -0.256409
H 3.945157 -0.841966 1.697783
H 2.037493 -3.660425 1.538481
H 3.695010 -4.237191 1.559179
H 3.237120 -2.846719 2.558602
H 2.133170 -3.305426 -1.030216
H 3.462785 -2.373300 -1.733728
H 3.766737 -3.947588 -1.006946
H 2.720615 2.898563 1.949858
H 3.091686 3.627838 0.399354
H 1.655759 4.150741 1.298972
H -1.628703 4.053775 -0.770354
H -2.582690 3.422790 0.605606
H -1.768387 4.995994 0.731051
H -2.715796 1.788673 -1.055120
H -0.961450 -0.699999 -2.635893
H -0.688121 -2.045904 0.910638
H -1.764653 -3.260343 0.180910
H -1.534583 -0.247747 1.840704
H -3.277249 -0.474566 2.122871
H -4.110295 -2.600060 0.733298
H -4.699480 -1.035793 0.140060
H -3.770565 -1.748427 -1.916753

7*R*9*S*2"*S*-**11**-c115, ΔG = 2.4906 kcal/mol, population = 0.28 %

C 2.020595 0.355316 1.196940
C 1.686464 1.636803 0.747999
C 0.457467 1.828146 0.084930
C -0.413856 0.748610 -0.081417
C -0.045680 -0.505776 0.401573
C 1.159024 -0.727573 1.033429
O 3.234322 0.140621 1.817249
C 4.351323 -0.060690 0.890527
C 4.221130 -1.326383 0.110983
C 3.721368 -1.449301 -1.123715
C 3.582303 -2.801532 -1.765231
C 3.219304 -0.308779 -1.963381
C 2.606284 2.807687 0.950327
O 0.227724 3.112898 -0.305494
C -0.729978 3.398928 -1.327882
C -1.815788 0.673680 -0.622497
N -2.184493 -0.761148 -0.558415
C -1.126691 -1.520132 0.137977
C -2.873594 -1.306546 1.631457
O -1.723187 -2.092221 1.309005
O -2.738073 1.376494 0.226554
C -3.925449 0.582922 0.261582
C -3.413562 -0.854844 0.268287
C -4.381002 -1.865579 -0.347973
O -4.501382 -1.664555 -1.750339
H 1.452591 -1.706577 1.384441
H 4.434060 0.814630 0.246137
H 5.223276 -0.089432 1.541292
H 4.509986 -2.230346 0.639067
H 3.942099 -3.599182 -1.115409
H 2.533890 -2.999758 -2.009655
H 4.136178 -2.843243 -2.707961
H 3.661724 -0.347053 -2.962460
H 2.135941 -0.397051 -2.091279
H 3.420593 0.668859 -1.533328
H 3.024771 3.150922 0.000530
H 2.065571 3.652715 1.378330
H 3.424394 2.543644 1.615570

H -0.529656 2.805658 -2.222022
H -1.748249 3.226515 -0.979581
H -0.601269 4.452988 -1.560436
H -1.936440 1.032503 -1.644730
H -0.769745 -2.363810 -0.453473
H -2.597959 -0.440486 2.240317
H -3.564456 -1.938316 2.187933
H -4.481496 0.839402 1.161466
H -4.546459 0.767703 -0.619955
H -4.026814 -2.879445 -0.130145
H -5.374130 -1.751846 0.089746
H -3.593632 -1.486871 -2.049603

7*R*9*S*2"*S*-**11**-c16, ΔG = 3.2128 kcal/mol, population = 0.08 %
C 2.122434 1.086753 -0.317718
C 1.513137 2.200655 0.283992
C 0.115752 2.330819 0.224269
C -0.653850 1.341301 -0.396160
C -0.004672 0.276582 -1.004806
C 1.366457 0.129966 -0.998123
O 3.484599 1.015422 -0.222152
C 4.121129 -0.278764 -0.370647
C 3.704045 -1.215614 0.716292
C 3.146835 -2.423438 0.591192
C 2.749258 -3.211896 1.808934
C 2.846903 -3.107384 -0.712801
C 2.323383 3.247960 0.996847
O -0.384487 3.430280 0.858734
C -1.616193 3.999747 0.403015
C -2.153327 1.107372 -0.425634
N -2.307747 -0.255820 -0.999932
C -1.016744 -0.679017 -1.590206
C -1.419030 -2.211852 0.059119
O -0.847969 -2.038599 -1.244009
O -2.762754 1.053360 0.859195
C -2.462605 -0.243161 1.383371
C -2.550070 -1.170797 0.161959
C -3.949016 -1.770490 0.023941
O -4.141417 -2.350530 -1.258844
H 1.821420 -0.721950 -1.474905
H 3.942837 -0.672092 -1.371171
H 5.181755 -0.039100 -0.294306
H 3.867251 -0.825453 1.717409
H 3.271823 -4.172610 1.838896
H 2.963587 -2.671706 2.731032
H 1.678874 -3.438714 1.780762
H 3.295901 -4.104327 -0.725694
H 1.766911 -3.243519 -0.824122
H 3.214727 -2.568798 -1.583539
H 3.382851 3.013745 0.950378
H 2.160176 4.232422 0.553101
H 2.028594 3.323438 2.046033
H -1.658319 4.996962 0.834734
H -1.625920 4.076708 -0.686093
H -2.472019 3.420184 0.747174
H -2.710080 1.843985 -1.000071
H -1.040029 -0.638375 -2.680157
H -0.661285 -2.042392 0.829000
H -1.776766 -3.236690 0.125389
H -1.461437 -0.258575 1.822069
H -3.197518 -0.467528 2.154438
H -4.096289 -2.559786 0.762738
H -4.688399 -0.982258 0.208472
H -3.835793 -1.676999 -1.886760

Geometry data of conformers of structure 7*S*9*S*2"*R*-**11**

7*S*9*S*2"*R*-**11**-c5, ΔG = 0.0000 kcal/mol, population = 30.68 %
C -2.216210 0.960217 -0.495271
C -1.641941 2.162103 -0.070876
C -0.256745 2.215960 0.202778
C 0.536933 1.089244 0.012835
C -0.089682 -0.108443 -0.405733
C -1.435806 -0.193004 -0.666207
O -3.555008 0.996241 -0.765927
C -4.268202 -0.241109 -0.985623
C -4.391367 -1.041180 0.270977
C -4.091746 -2.329908 0.454393
C -4.279487 -2.976841 1.799270
C -3.548224 -3.247919 -0.604459
C -2.467974 3.405977 0.104409
O 0.170700 3.436168 0.625266
C 1.554158 3.633915 0.913262
C 1.926349 0.618774 0.385373
N 2.142726 -0.438779 -0.620119
C 0.942484 -1.215666 -0.549276

C 2.546510 -2.175476 0.897118
O 1.126373 -2.056368 0.635291
O 3.195685 1.233201 0.410903
C 4.037520 0.051479 0.607158
C 3.268654 -1.155144 -0.043982
C 4.077054 -1.854492 -1.132507
O 4.485195 -0.940516 -2.141627
H -1.864454 -1.123933 -0.997913
H -5.247997 0.098485 -1.323100
H -3.811688 -0.794560 -1.806261
H -4.777981 -0.475680 1.114560
H -4.970137 -3.822784 1.730820
H -3.329102 -3.377184 2.165784
H -4.666388 -2.274129 2.537168
H -2.578150 -3.647068 -0.292608
H -4.211698 -4.107587 -0.734546
H -3.428483 -2.775252 -1.577054
H -2.386189 3.786558 1.124830
H -2.116945 4.202539 -0.556028
H -3.513475 3.209276 -0.113364
H 1.651539 4.683182 1.180219
H 1.876160 3.013597 1.750206
H 2.172462 3.413268 0.042918
H 1.796741 0.174280 1.384943
H 0.780424 -1.862616 -1.412299
H 2.870033 -3.197041 0.684252
H 2.714179 -1.964975 1.952781
H 4.985348 0.246112 0.114905
H 4.191089 -0.107981 1.676098
H 4.978338 -2.300471 -0.709520
H 3.472143 -2.661863 -1.562482
H 3.708077 -0.392848 -2.330524

7*S*9*S*2"*R*-**11**-c15, ΔG = 0.3370 kcal/mol, population = 17.36 %

C -1.850722 0.320158 0.122869
C -1.472623 1.656477 -0.046711
C -0.103025 1.997702 -0.054058
C 0.865177 1.003568 0.059663
C 0.437518 -0.331937 0.240202
C -0.889582 -0.692333 0.267879
O -3.191929 0.070753 0.119903
C -3.644768 -1.289343 0.301719
C -5.135137 -1.276581 0.324507
C -5.950893 -1.696271 -0.647734
C -7.443698 -1.633066 -0.475273
C -5.500859 -2.253210 -1.969189
C -2.497181 2.743447 -0.216801
O 0.129097 3.329581 -0.200712
C 1.472493 3.805582 -0.274332
C 2.350294 0.913642 0.337808
N 2.658290 -0.420693 -0.210551
C 1.640952 -1.254889 0.353443
C 3.536999 -1.320152 1.764046
O 2.101639 -1.512948 1.719312
O 3.474721 1.649450 -0.090358
C 4.541500 0.771982 0.394572
C 3.952901 -0.685533 0.396051
C 4.766232 -1.652467 -0.459100
O 4.895882 -1.181646 -1.793996
H -1.168590 -1.727776 0.389125
H -3.247650 -1.912399 -0.500307
H -3.254953 -1.665444 1.252448
H -5.576755 -0.874574 1.231480
H -7.893795 -1.009395 -1.253566
H -7.885693 -2.628874 -0.577821
H -7.723667 -1.229035 0.497509
H -4.421294 -2.244685 -2.101405
H -5.852453 -3.282533 -2.086745
H -5.946306 -1.676675 -2.785010
H -2.420186 3.480437 0.586029
H -2.338718 3.282689 -1.153278
H -3.501916 2.331441 -0.215873
H 1.395300 4.876205 -0.446111
H 2.008299 3.623737 0.657614
H 2.011451 3.335537 -1.097306
H 2.412868 0.897937 1.437388
H 1.516505 -2.210557 -0.157223
H 4.027514 -2.285092 1.911068
H 3.765555 -0.675532 2.612222
H 5.377623 0.873483 -0.290217
H 4.833778 1.080887 1.399784
H 5.772369 -1.764203 -0.052175
H 4.284868 -2.637623 -0.440692
H 4.014223 -0.873813 -2.053135

7*S*9*S*2"*R*-**11**-c10, ΔG = 0.4945 kcal/mol, population = 13.31 %

C -1.826586 0.192852 -0.359351
C -1.506403 1.543587 -0.185230
C -0.163804 1.918405 0.037627
C 0.840902 0.954460 0.032100
C 0.470535 -0.399163 -0.141592
C -0.831233 -0.796428 -0.339678
O -3.146822 -0.091689 -0.551657
C -3.532888 -1.463242 -0.789188
C -5.001054 -1.483978 -1.042921
C -5.944983 -2.002370 -0.251240
C -7.392350 -1.959400 -0.658960
C -5.693513 -2.663466 1.075053
C -2.566351 2.609157 -0.221117
O 0.008546 3.254751 0.220967
C 1.324698 3.776283 0.401064
C 2.292484 0.846559 0.447583
N 2.726964 -0.314540 -0.350739
C 1.704855 -1.286892 -0.106899
C 3.450325 -1.623360 1.450387
O 2.038442 -1.839065 1.207610
O 3.413126 1.694371 0.326464
C 4.470941 0.760514 0.716451
C 3.966267 -0.674109 0.319152
C 4.914090 -1.389996 -0.638532
O 5.152632 -0.617198 -1.807422
H -1.064966 -1.840653 -0.479859
H -2.991398 -1.832853 -1.665821
H -3.250100 -2.073477 0.068959
H -5.307517 -1.020563 -1.976145
H -7.806592 -2.970570 -0.716871
H -7.985061 -1.420822 0.086628
H -7.527920 -1.474467 -1.625628
H -6.293522 -2.179229 1.850784
H -6.011681 -3.709742 1.042398
H -4.651863 -2.635304 1.386739
H -2.329899 3.365386 -0.972588
H -3.538041 2.180238 -0.447460
H -2.629685 3.129136 0.737849
H 1.206885 4.855229 0.461835
H 1.773737 3.406184 1.323039
H 1.967838 3.520019 -0.441253
H 2.242618 0.578993 1.515033
H 1.685672 -2.101978 -0.831365
H 3.973434 -2.581944 1.421628
H 3.560425 -1.190647 2.444201
H 5.366569 1.043022 0.172084
H 4.640275 0.835514 1.792162
H 5.878323 -1.563228 -0.158401
H 4.488330 -2.366309 -0.899563
H 4.286318 -0.284159 -2.086242

7*S*9*S*2"*R*-**11**-c3, ΔG = 0.5384 kcal/mol, population = 12.35 %

C -2.239181 0.940029 0.506359
C -1.655302 2.154833 0.134705
C -0.255219 2.231901 -0.040173
C 0.528200 1.090018 0.093743
C -0.106680 -0.118441 0.466333
C -1.461946 -0.216175 0.669595
O -3.589758 0.961600 0.711150
C -4.306461 -0.285940 0.846838
C -4.363821 -1.036306 -0.444966
C -4.062794 -2.319623 -0.660652
C -4.181129 -2.914459 -2.037162
C -3.586164 -3.281237 0.391422
C -2.488465 3.388810 -0.073016
O 0.190940 3.476628 -0.359309
C 1.573546 3.672733 -0.653186
C 1.995244 0.744785 0.233891
N 1.992579 -0.667051 -0.192770
C 0.916311 -1.239474 0.557999
C 2.923704 -1.546108 1.767174
O 1.482887 -1.460154 1.890044
O 3.176006 1.212112 -0.379613
C 4.117171 0.194938 0.091627
C 3.281515 -1.119365 0.305091
C 3.801961 -2.291897 -0.520802
O 3.855600 -1.969693 -1.904117
H -1.901844 -1.162435 0.937240
H -3.887860 -0.869188 1.667304
H -5.303478 0.036948 1.148550
H -4.700551 -0.436232 -1.286012
H -4.524926 -2.182511 -2.767954
H -3.214309 -3.306897 -2.367532
H -4.877932 -3.758081 -2.037304
H -4.269955 -4.132027 0.461798
H -2.609301 -3.688646 0.113578
H -3.505601 -2.841897 1.383342

H -3.546162 3.141415 -0.084457
H -2.315703 4.119684 0.721967
H -2.224914 3.877365 -1.012014
H 1.664783 4.712859 -0.955820
H 2.193987 3.492756 0.224901
H 1.899225 3.020269 -1.463912
H 2.181412 0.815502 1.317417
H 0.561832 -2.194771 0.168722
H 3.243837 -2.572410 1.961429
H 3.366454 -0.890482 2.516265
H 4.875247 0.077347 -0.676422
H 4.571846 0.529559 1.025870
H 4.812657 -2.557188 -0.207157
H 3.158133 -3.163173 -0.350500
H 3.019009 -1.523568 -2.104769

7*S*9*S*2"*R*-**11**-c1, ΔG = 0.8013 kcal/mol, population = 7.92 %

C -2.061163 0.468012 0.735854
C -1.672040 1.680913 0.160721
C -0.308638 1.908302 -0.132757
C 0.627948 0.902617 0.084644
C 0.186909 -0.312999 0.660335
C -1.127360 -0.548870 0.984148
O -3.384254 0.347707 1.046143
C -3.929711 -0.968060 1.301214
C -3.827069 -1.909557 0.134763
C -4.381084 -1.706306 -1.064095
C -4.199483 -2.693574 -2.182059
C -5.190092 -0.488604 -1.410293
C -2.681033 2.752126 -0.145535
O -0.054230 3.142081 -0.645759
C 1.264231 3.459592 -1.090216
C 2.133980 0.769368 0.153624
N 2.298836 -0.677451 -0.082062
C 1.357117 -1.270664 0.818424
C 3.455646 -1.145914 1.898546
O 2.026242 -1.228229 2.120115
O 3.201547 1.291665 -0.606152
C 4.298811 0.479340 -0.078089
C 3.664382 -0.887237 0.370105
C 4.289625 -2.088037 -0.333194
O 4.211431 -1.959248 -1.746688
H -1.421545 -1.494713 1.412331
H -3.452977 -1.395586 2.185503
H -4.966742 -0.756517 1.559598
H -3.242406 -2.809833 0.290180
H -3.720867 -2.215591 -3.042476
H -3.590342 -3.544837 -1.877784
H -5.168259 -3.064871 -2.529960
H -6.117220 -0.778682 -1.911986
H -5.432235 0.120048 -0.542140
H -4.636061 0.142914 -2.112133
H -2.597894 3.587312 0.555953
H -2.517359 3.160490 -1.143302
H -3.690215 2.354750 -0.082481
H 1.193294 4.447593 -1.538125
H 1.967158 3.486075 -0.257490
H 1.612457 2.739900 -1.831830
H 2.373510 1.015779 1.200334
H 1.114302 -2.308505 0.586628
H 3.924157 -2.084976 2.202252
H 3.847621 -0.339578 2.517723
H 5.020178 0.352642 -0.879371
H 4.756452 0.997164 0.766876
H 5.344892 -2.174324 -0.070313
H 3.783946 -3.001737 0.001463
H 3.311393 -1.652682 -1.934798

7*S*9*S*2"*R*-**11**-c2, ΔG = 1.0197 kcal/mol, population = 5.48 %

C -2.011742 0.390404 -0.785786
C -1.609493 1.685863 -0.447345
C -0.257390 1.935260 -0.122685
C 0.674066 0.902592 -0.170468
C 0.218441 -0.396054 -0.500006
C -1.091716 -0.668665 -0.811450
O -3.325102 0.237043 -1.120353
C -3.876278 -1.096682 -1.226782
C -3.873716 -1.862753 0.065076
C -4.570504 -1.527197 1.154833
C -4.484697 -2.343279 2.413610
C -5.462806 -0.321313 1.235226
C -2.589781 2.825346 -0.418991
O -0.001229 3.231542 0.199822
C 1.333238 3.625089 0.516640
C 2.086827 0.642549 0.306889
N 2.483124 -0.466122 -0.582027

C 1.381231 -1.375522 -0.490425
C 3.002795 -1.993326 1.114166
O 1.596301 -2.076562 0.776823
O 3.270919 1.408596 0.330138
C 4.235142 0.361914 0.674655
C 3.651612 -0.984026 0.110557
C 4.597588 -1.676895 -0.865631
O 4.953998 -0.817568 -1.940263
H -1.389553 -1.671366 -1.076171
H -4.888316 -0.914680 -1.585310
H -3.346931 -1.648587 -2.005952
H -3.242785 -2.744034 0.106079
H -4.122905 -1.730532 3.245198
H -5.474038 -2.710249 2.703230
H -3.816999 -3.197222 2.298991
H -5.036447 0.416993 1.921666
H -5.607442 0.166706 0.273749
H -6.439677 -0.598984 1.640674
H -3.598828 2.469819 -0.605603
H -2.566164 3.332670 0.547257
H -2.339479 3.575456 -1.173608
H 1.290755 4.698140 0.685402
H 1.688430 3.127853 1.419689
H 2.013443 3.405317 -0.306699
H 1.955227 0.277218 1.337840
H 1.346057 -2.113112 -1.293101
H 3.457450 -2.982182 1.019900
H 3.083227 -1.665026 2.149959
H 5.178058 0.626384 0.206203
H 4.348909 0.319013 1.759360
H 5.519011 -1.967749 -0.359144
H 4.118217 -2.588893 -1.241315
H 4.130259 -0.391321 -2.221658

7*S*9*S*2"*R*-**11**-c23, ΔG = 1.1785 kcal/mol, population = 4.19 %

C -2.244050 0.489283 -1.073734
C -1.852665 1.736568 -0.596077
C -0.517575 1.922497 -0.158139
C 0.392308 0.877565 -0.254717
C -0.048805 -0.369784 -0.768689
C -1.340165 -0.572917 -1.184159
O -3.554086 0.276350 -1.453025
C -4.439527 -0.054566 -0.332234
C -4.115034 -1.379803 0.273826
C -3.336766 -1.595521 1.340480
C -3.024973 -2.994639 1.793093
C -2.671692 -0.519010 2.150542
C -2.814859 2.887114 -0.518524
O -0.266904 3.167348 0.327240
C 1.059628 3.514644 0.723614
C 1.757459 0.532965 0.301949
N 2.215094 -0.475750 -0.670969
C 1.098527 -1.365435 -0.779415
C 2.567274 -2.198526 0.875184
O 1.194879 -2.212823 0.411664
O 2.942899 1.264201 0.517006
C 3.860492 0.164232 0.819482
C 3.312676 -1.094649 0.054607
C 4.334236 -1.689928 -0.909709
O 4.796305 -0.719934 -1.840212
H -1.676153 -1.525668 -1.567953
H -4.396294 0.755262 0.396644
H -5.431724 -0.060317 -0.779456
H -4.504744 -2.242573 -0.258429
H -1.945081 -3.170664 1.760355
H -3.335459 -3.146064 2.831283
H -3.515779 -3.742809 1.170655
H -1.587525 -0.572321 2.010345
H -2.989001 0.485096 1.881627
H -2.863023 -0.670866 3.216225
H -2.374865 3.785051 -0.954115
H -3.735368 2.652786 -1.047181
H -3.061023 3.129624 0.518839
H 1.378085 2.934380 1.589396
H 1.765636 3.359657 -0.093032
H 1.021258 4.569021 0.985168
H 1.527281 0.056032 1.268254
H 1.123129 -2.004450 -1.662766
H 3.017204 -3.179443 0.705988
H 2.560732 -1.992926 1.945094
H 4.843768 0.459681 0.466987
H 3.878365 -0.005456 1.897615
H 5.203072 -2.056695 -0.361367
H 3.881702 -2.542184 -1.430707
H 4.006579 -0.250126 -2.148118

7*S*9*S*2"*R*-**11**-c27, ΔG = 1.2751 kcal/mol, population =

3.56 %
C -2.131009 0.279504 1.284393
C -1.944850 1.382046 0.457964
C -0.660607 1.622336 -0.094781
C 0.378263 0.736521 0.157961
C 0.146767 -0.359582 1.030587
C -1.081684 -0.597081 1.590436
O -3.379659 0.022584 1.812872
C -4.084571 -1.076234 1.148506
C -4.466352 -0.722227 -0.250447
C -3.785403 -1.018300 -1.362874
C -4.238836 -0.494309 -2.697229
C -2.523523 -1.832477 -1.412035
C -3.065282 2.325481 0.130627
O -0.601564 2.732911 -0.876275
C 0.619770 3.067220 -1.534412
C 1.880398 0.690759 -0.026526
N 2.121400 -0.764503 -0.005055
C 1.406218 -1.201527 1.156446
C 3.642946 -0.763414 1.775051
O 2.285636 -0.854134 2.273135
O 2.752676 1.097584 -1.055995
C 3.988852 0.459065 -0.600938
C 3.558106 -0.813535 0.213798
C 4.147993 -2.102931 -0.349449
O 3.814581 -2.269736 -1.720973
H -1.257303 -1.447995 2.235498
H -3.463283 -1.971105 1.198105
H -4.964319 -1.234679 1.769321
H -5.346261 -0.093169 -0.344172
H -4.418465 -1.314106 -3.399169
H -5.149817 0.098074 -2.612614
H -3.458834 0.132931 -3.140623
H -2.606195 -2.613006 -2.173292
H -1.682160 -1.195015 -1.701220
H -2.270182 -2.299506 -0.464102
H -2.796354 3.351087 0.391117
H -3.280329 2.314821 -0.940050
H -3.966203 2.046676 0.668169
H 0.947211 2.259094 -2.188798
H 0.402618 3.955339 -2.122340
H 1.407647 3.290014 -0.814396
H 2.270889 1.155946 0.892423
H 1.217450 -2.275388 1.182643
H 4.227309 -1.603015 2.158741
H 4.076616 0.165825 2.142890
H 4.563023 0.202530 -1.485821
H 4.547229 1.158110 0.024338
H 5.236300 -2.084712 -0.276511
H 3.785578 -2.951148 0.243603
H 2.872533 -2.053474 -1.792183

7*S*9*S*2"*R*-**11**-c26, ΔG = 1.4602 kcal/mol, population = 2.60 %
C -2.095832 -0.213183 -1.321125
C -1.915522 1.117763 -0.961833
C -0.659009 1.530838 -0.448741
C 0.382642 0.618315 -0.350133
C 0.154097 -0.726690 -0.743170
C -1.056181 -1.147752 -1.230177
O -3.323005 -0.635857 -1.789693
C -4.097585 -1.389749 -0.801367
C -4.528921 -0.528391 0.339300
C -3.907484 -0.393325 1.515910
C -4.399892 0.601342 2.530024
C -2.676702 -1.147407 1.934223
C -3.019272 2.127583 -1.084587
O -0.617886 2.842619 -0.095480
C 0.602263 3.404084 0.387074
C 1.732065 0.530430 0.330592
N 2.396845 -0.498739 -0.490034
C 1.422293 -1.545071 -0.566985
C 2.854823 -1.983248 1.261541
O 1.538148 -2.242157 0.715510
O 2.788067 1.440896 0.536519
C 3.818474 0.520094 1.019957
C 3.507125 -0.876465 0.369445
C 4.671607 -1.417704 -0.454444
O 5.070279 -0.493914 -1.458301
H -1.220248 -2.172518 -1.536720
H -4.953711 -1.755388 -1.365096
H -3.508308 -2.247940 -0.475817
H -5.389671 0.103159 0.141938
H -3.619613 1.338663 2.744657
H -5.287199 1.130836 2.183090
H -4.634950 0.109286 3.478519
H -2.815810 -1.576002 2.930463
H -2.401455 -1.945463 1.249935

H -1.826667 -0.461154 1.999562
H -3.888741 1.683550 -1.559561
H -3.314314 2.502271 -0.101941
H -2.691068 2.989322 -1.669045
H 1.407751 3.271236 -0.335749
H 0.402255 4.463119 0.528574
H 0.893998 2.957238 1.337583
H 1.492145 0.127803 1.327644
H 1.602758 -2.261361 -1.369258
H 3.444839 -2.902249 1.235827
H 2.735141 -1.669462 2.298027
H 4.777882 0.914545 0.700218
H 3.773255 0.467143 2.109232
H 5.536257 -1.598879 0.185609
H 4.378770 -2.374873 -0.902523
H 4.250511 -0.173739 -1.864182

7*S*9*S*2"*R*-**11**-c33, ΔG = 1.8637 kcal/mol, population = 1.32 %

C -2.227133 0.582699 1.126111
C -1.866852 1.784910 0.524787
C -0.533142 1.964693 0.078980
C 0.383277 0.929821 0.211960
C -0.021348 -0.264797 0.863945
C -1.301707 -0.449411 1.319520
O -3.530425 0.377331 1.532018
C -4.416194 -0.083813 0.458102
C -4.067509 -1.455757 -0.014438
C -3.297486 -1.760209 -1.065280
C -2.954316 -3.190149 -1.376788
C -2.668884 -0.755886 -1.988825
C -2.856825 2.898261 0.333615
O -0.302185 3.181302 -0.482327
C 1.006512 3.503665 -0.951483
C 1.876325 0.726737 0.063337
N 1.937849 -0.727719 -0.175734
C 1.119862 -1.268944 0.868121
C 3.360768 -1.236039 1.613114
O 1.979846 -1.239704 2.051517
O 2.839414 1.195999 -0.851941
C 3.966153 0.333789 -0.490942
C 3.345744 -0.998131 0.066633
C 3.801121 -2.232287 -0.706110
O 3.512737 -2.111195 -2.092555
H -1.618714 -1.374568 1.779201
H -5.404761 -0.064957 0.913045
H -4.395345 0.649226 -0.348786
H -4.428660 -2.268720 0.608579
H -1.869886 -3.335235 -1.338488
H -3.419893 -3.882673 -0.675618
H -3.269701 -3.454469 -2.390522
H -3.016130 0.261037 -1.825752
H -1.583154 -0.760768 -1.850609
H -2.859050 -1.025143 -3.031198
H -2.456499 3.838884 0.714467
H -3.074375 3.055805 -0.726107
H -3.786971 2.677428 0.850730
H 1.319481 2.822831 -1.742914
H 0.937572 4.516563 -1.339861
H 1.734211 3.473523 -0.139646
H 2.275508 0.965476 1.061880
H 0.801348 -2.297236 0.693128
H 3.820306 -2.197687 1.853104
H 3.885115 -0.449401 2.154555
H 4.546401 0.168439 -1.393465
H 4.574313 0.832897 0.265532
H 4.879229 -2.365563 -0.606165
H 3.312391 -3.118515 -0.283815
H 2.609062 -1.764841 -2.145213

7*S*9*S*2"*R*-**11**-c12, ΔG = 1.9679 kcal/mol, population = 1.10 %

C -2.207652 0.992642 -0.444781
C -1.670674 2.195085 0.024747
C -0.285867 2.285059 0.291142
C 0.543656 1.194014 0.051634
C -0.046361 -0.006389 -0.407880
C -1.390665 -0.126548 -0.664178
O -3.548647 0.995753 -0.707043
C -4.218650 -0.251760 -0.993753
C -4.314109 -1.123573 0.216858
C -3.980616 -2.412425 0.325848
C -4.148066 -3.140380 1.631374
C -3.416984 -3.253432 -0.785193
C -2.537399 3.402029 0.254622
O 0.103670 3.502531 0.756724
C 1.479953 3.732669 1.053484
C 1.949024 0.752025 0.395730

N 2.197874 -0.254622 -0.657510
C 1.020918 -1.072351 -0.595425
C 2.669592 -2.051975 0.769852
O 1.241289 -1.937127 0.562166
O 3.196144 1.408214 0.450843
C 4.066641 0.249142 0.621501
C 3.355160 -0.949972 -0.108991
C 4.257926 -1.537084 -1.187763
O 3.586411 -2.480549 -2.020402
H -1.790216 -1.059225 -1.025965
H -5.209898 0.071314 -1.313752
H -3.743384 -0.743349 -1.842685
H -4.712851 -0.617010 1.091684
H -4.550265 -2.491249 2.409094
H -4.817836 -3.997813 1.514973
H -3.187090 -3.537582 1.972764
H -4.061318 -4.118228 -0.967229
H -3.307947 -2.720766 -1.727559
H -2.438234 -3.649017 -0.496736
H -2.241991 4.225150 -0.400880
H -3.581401 3.168701 0.067756
H -2.434233 3.764917 1.279232
H 2.104852 3.567018 0.175657
H 1.543899 4.773099 1.362050
H 1.821734 3.090225 1.865551
H 1.837704 0.260052 1.375054
H 0.865304 -1.699761 -1.473334
H 3.000486 -3.043874 0.459602
H 2.863687 -1.922172 1.834189
H 5.028807 0.497753 0.179768
H 4.193093 0.043390 1.686139
H 4.671342 -0.721438 -1.787399
H 5.085928 -2.071672 -0.719042
H 3.007904 -1.990511 -2.616710

7*S*9*S*2"*R*-**11**-c41, ΔG = 3.2536 kcal/mol, population = 0.13 %

C -2.215403 0.546416 -1.074130
C -1.871269 1.780352 -0.530376
C -0.547748 1.987765 -0.066836
C 0.397771 0.980025 -0.204899
C 0.003610 -0.254379 -0.783328
C -1.275566 -0.479106 -1.224067
O -3.512323 0.309311 -1.483565
C -4.402417 -0.108348 -0.396390
C -4.032225 -1.443574 0.158837
C -3.272293 -1.672040 1.235925
C -2.905903 -3.074528 1.634008
C -2.676626 -0.604727 2.110010
C -2.872300 2.893260 -0.407126
O -0.344872 3.215226 0.482089
C 0.962444 3.582470 0.921497
C 1.769449 0.650177 0.343058
N 2.274100 -0.284570 -0.683569
C 1.184925 -1.207927 -0.831159
C 2.687564 -2.093091 0.749009
O 1.303932 -2.096002 0.323698
O 2.923254 1.410349 0.619861
C 3.862615 0.327341 0.894366
C 3.392567 -0.895997 0.023209
C 4.503976 -1.349559 -0.916105
O 4.058265 -2.297378 -1.883842
H -1.575978 -1.421974 -1.658437
H -4.408010 0.669683 0.367248
H -5.385064 -0.136811 -0.863601
H -4.366752 -2.298083 -0.422058
H -1.818326 -3.198336 1.623553
H -3.235540 -3.287625 2.655285
H -3.344157 -3.815258 0.965271
H -1.589632 -0.592941 1.982791
H -3.044433 0.394170 1.890094
H -2.871695 -0.822497 3.163467
H -2.466468 3.821438 -0.811851
H -3.786712 2.646233 -0.940575
H -3.120563 3.089246 0.639419
H 0.886520 4.621164 1.232989
H 1.282301 2.970443 1.764727
H 1.688337 3.489034 0.113090
H 1.547092 0.107275 1.275685
H 1.229122 -1.809894 -1.738923
H 3.154736 -3.036696 0.464164
H 2.702692 -1.995263 1.834173
H 4.853980 0.680561 0.620526
H 3.838457 0.086587 1.958740
H 4.933211 -0.471880 -1.407124
H 5.291228 -1.840105 -0.341255
H 3.534323 -1.824090 -2.541295

Geometry data of conformers of structure 7*S*9*S*2"*S*-**11**

7*S*9*S*2"*S*-**11**-c3, ΔG = 0.0000 kcal/mol, population = 33.13 %

C 2.153144 0.810084 -0.812616
C 1.658758 2.096278 -0.571455
C 0.297135 2.266677 -0.234502
C -0.543317 1.159771 -0.143651
C 0.015304 -0.120767 -0.369009
C 1.333342 -0.324598 -0.683956
O 3.462655 0.734310 -1.188020
C 4.132833 -0.547097 -1.180077
C 4.329997 -1.060109 0.209798
C 4.027358 -2.270945 0.685815
C 4.297303 -2.618681 2.124112
C 3.405946 -3.380983 -0.114702
C 2.549003 3.304232 -0.670513
O -0.058543 3.564077 -0.029496
C -1.316305 3.891524 0.566122
C -2.046863 1.006307 0.094858
N -2.263836 -0.308863 -0.436311
C -1.085461 -1.056090 0.020953
C -2.743876 -2.469372 0.231542
O -1.404452 -2.370122 -0.361419
O -2.458556 0.893015 1.500055
C -3.344661 -0.244254 1.637743
C -3.325843 -1.004788 0.270529
C -4.651775 -0.877527 -0.473157
O -4.534481 -1.312713 -1.821195
H 1.713613 -1.321844 -0.825931
H 3.605917 -1.247177 -1.828119
H 5.091737 -0.322937 -1.648446
H 4.779029 -0.336406 0.884757
H 4.738079 -1.783181 2.667749
H 3.369764 -2.909590 2.626888
H 4.974291 -3.475298 2.196158
H 4.052980 -4.262668 -0.100786
H 2.456169 -3.684221 0.336434
H 3.224725 -3.123254 -1.155948
H 3.591450 3.008069 -0.745968
H 2.301222 3.908001 -1.548212
H 2.422307 3.948049 0.200724
H -2.122507 3.836581 -0.166205
H -1.537184 3.240953 1.412989
H -1.220164 4.918416 0.910355
H -2.667773 1.758798 -0.384263
H -1.040505 -1.067142 1.123157
H -3.331096 -3.123772 -0.405049
H -2.662455 -2.888166 1.236097
H -2.982969 -0.857468 2.462645
H -4.351505 0.107286 1.873919
H -5.415505 -1.493109 0.004303
H -4.986873 0.165810 -0.430522
H -3.710000 -0.926488 -2.153416

7*S*9*S*2"*S*-**11**-c17, ΔG = 0.2567 kcal/mol, population = 21.47 %

C 1.815291 0.326740 -0.246100
C 1.482140 1.684836 -0.305643
C 0.127167 2.072436 -0.221656
C -0.870004 1.106483 -0.095872
C -0.476555 -0.249775 -0.016523
C 0.830704 -0.662132 -0.072271
O 3.141451 0.030965 -0.345824
C 3.551845 -1.353906 -0.290977
C 5.027392 -1.399661 -0.492814
C 5.950701 -1.723310 0.417877
C 7.409878 -1.744461 0.053096
C 5.663192 -2.090642 1.846738
C 2.540656 2.742108 -0.459102
O -0.059966 3.418600 -0.282851
C -1.335135 3.994240 0.010145
C -2.398760 1.176775 -0.087859
N -2.724682 -0.177252 -0.433283
C -1.745970 -0.963406 0.328326
C -3.604682 -2.110507 0.479033
O -2.192601 -2.277724 0.112444
O -3.018736 1.364382 1.230206
C -4.075866 0.387511 1.391597
C -3.967836 -0.604614 0.186535
C -5.137835 -0.457488 -0.781289
O -4.891597 -1.142556 -2.002007
H 1.080299 -1.707913 0.012309
H 3.249408 -1.783534 0.664314
H 3.042634 -1.901404 -1.090068
H 5.358329 -1.138745 -1.493789
H 7.572107 -1.472657 -0.989801
H 7.973465 -1.051609 0.685176

H 7.834711 -2.738639 0.222572
H 5.994133 -3.113640 2.049182
H 6.231538 -1.440755 2.518262
H 4.611646 -2.015382 2.114116
H 2.466080 3.483149 0.339122
H 3.532813 2.300905 -0.436954
H 2.422711 3.282333 -1.401866
H -1.146016 5.049340 0.192135
H -2.013361 3.896449 -0.838195
H -1.780266 3.544042 0.898049
H -2.831279 1.898893 -0.775294
H -1.862513 -0.779653 1.409765
H -4.180873 -2.794763 -0.135884
H -3.732596 -2.348888 1.536332
H -3.937439 -0.104414 2.354042
H -5.042561 0.896117 1.390790
H -6.045370 -0.879119 -0.346808
H -5.317168 0.608783 -0.964546
H -3.980429 -0.924097 -2.249664

7*S*9*S*2"*S*-**11**-c10, ΔG = 0.5924 kcal/mol, population = 12.18 %

C 1.830242 0.264641 0.239054
C 1.539147 1.575136 -0.156645
C 0.192720 1.973156 -0.304795
C -0.835767 1.062309 -0.069511
C -0.486973 -0.241796 0.352498
C 0.808502 -0.658093 0.526648
O 3.151818 -0.052683 0.333805
C 3.519236 -1.369939 0.802134
C 5.005474 -1.423903 0.893897
C 5.830532 -2.128342 0.113056
C 7.316123 -2.098748 0.347061
C 5.400555 -2.997163 -1.035732
C 2.633408 2.568788 -0.434127
O 0.046437 3.270652 -0.686569
C -1.237427 3.898813 -0.669733
C -2.354404 1.133586 -0.244850
N -2.675507 -0.263060 -0.311844
C -1.803345 -0.858792 0.708164
C -3.696242 -1.942290 0.904806
O -2.257407 -2.187790 0.742741
O -3.107753 1.614292 0.920758
C -4.197587 0.697065 1.183121
C -3.987685 -0.539505 0.247658
C -5.043988 -0.609428 -0.850731
O -4.686307 -1.548110 -1.856284
H 1.022512 -1.659844 0.864110
H 3.073050 -1.527882 1.788893
H 3.117113 -2.120341 0.121234
H 5.435544 -0.819427 1.687055
H 7.838229 -1.738802 -0.544566
H 7.582606 -1.457840 1.187362
H 7.694509 -3.106125 0.545822
H 5.928165 -2.696431 -1.945312
H 5.674894 -4.039210 -0.846303
H 4.332413 -2.958263 -1.237103
H 3.605675 2.150365 -0.190842
H 2.638131 2.864576 -1.486472
H 2.484304 3.480277 0.147593
H -1.784578 3.662785 0.243606
H -1.041715 4.967484 -0.711382
H -1.825393 3.610997 -1.541913
H -2.691617 1.682533 -1.120129
H -2.029699 -0.437294 1.702145
H -4.219892 -2.746255 0.397058
H -3.942871 -1.938615 1.967896
H -4.170268 0.432969 2.239949
H -5.147427 1.191351 0.967119
H -6.003154 -0.923108 -0.436382
H -5.175329 0.390080 -1.282555
H -3.747908 -1.392492 -2.042098

7*S*9*S*2"*S*-**11**-c2, ΔG = 0.8616 kcal/mol, population = 7.73 %

C 2.068456 0.462937 0.655794
C 1.717457 1.717093 0.147448
C 0.357247 2.015883 -0.091742
C -0.620598 1.050789 0.138828
C -0.211393 -0.199025 0.663923
C 1.094218 -0.509415 0.942019
O 3.395497 0.262582 0.889792
C 3.876875 -1.079295 1.138333
C 3.674481 -2.026218 -0.010855
C 4.197518 -1.873188 -1.230891
C 3.919771 -2.863791 -2.325971
C 5.066301 -0.712167 -1.623952
C 2.762143 2.757279 -0.149904

O 0.151391 3.279121 -0.552760
C -1.171282 3.799794 -0.697408
C -2.126637 1.002278 -0.128069
N -2.347105 -0.415191 -0.125562
C -1.502895 -0.885575 0.980393
C -3.327174 -2.085982 1.135435
O -1.868326 -2.239279 1.072172
O -2.982300 1.494559 0.959545
C -4.026828 0.521804 1.206396
C -3.670917 -0.751693 0.369818
C -4.645503 -0.968081 -0.783730
O -4.154486 -1.935789 -1.701746
H 1.354175 -1.467432 1.363393
H 4.933820 -0.922999 1.350851
H 3.414502 -1.469861 2.046684
H 3.042931 -2.887561 0.178413
H 4.852832 -3.293398 -2.703083
H 3.276382 -3.675279 -1.985812
H 3.436562 -2.371843 -3.175849
H 5.351530 -0.090493 -0.778452
H 5.970682 -1.067648 -2.125449
H 4.538980 -0.075067 -2.341102
H 2.667222 3.613901 0.522689
H 3.759195 2.341099 -0.038766
H 2.650081 3.140614 -1.165532
H -1.780842 3.588580 0.182088
H -1.052423 4.874810 -0.807849
H -1.653580 3.401759 -1.590912
H -2.446206 1.472525 -1.054115
H -1.820208 -0.423488 1.930480
H -3.764261 -2.955673 0.654768
H -3.638086 -2.031685 2.180178
H -4.058726 0.325009 2.277553
H -4.988512 0.935162 0.894058
H -5.604446 -1.328679 -0.408743
H -4.820763 -0.009761 -1.287278
H -3.218480 -1.721885 -1.834764

7*S*9*S*2"*S*-**11**-c5, ΔG = 0.8641 kcal/mol, population = 7.69 %

C 2.264613 0.974973 0.273728
C 1.727340 2.134380 -0.293465
C 0.326024 2.254330 -0.436875
C -0.508146 1.209510 -0.047123
C 0.087538 0.067857 0.541297
C 1.437865 -0.067693 0.727083
O 3.623501 0.940578 0.399460
C 4.279044 -0.320660 0.670390
C 4.152201 -1.265620 -0.480280
C 3.731305 -2.533067 -0.457151
C 3.660675 -3.339676 -1.724746
C 3.290095 -3.273023 0.774256
C 2.610141 3.260446 -0.756832
O -0.066833 3.439044 -0.978100
C -1.448692 3.802082 -1.017220
C -2.011750 0.960333 -0.189770
N -2.052797 -0.467700 -0.054591
C -1.083720 -0.732385 1.016398
C -2.728874 -2.119828 1.414572
O -1.270414 -2.105543 1.248422
O -2.847533 1.443476 0.916925
C -3.745526 0.380519 1.319902
C -3.287706 -0.913186 0.567728
C -4.300161 -1.351844 -0.485787
O -3.752365 -2.336788 -1.351911
H 1.838808 -0.949241 1.197841
H 5.318786 -0.026977 0.817313
H 3.918736 -0.735390 1.611609
H 4.444788 -0.836152 -1.434691
H 2.639095 -3.693420 -1.895224
H 3.975216 -2.760121 -2.592578
H 4.292761 -4.230014 -1.654681
H 2.250632 -3.597458 0.665090
H 3.888094 -4.179573 0.903350
H 3.373609 -2.691001 1.689559
H 2.438579 4.162052 -0.163180
H 3.657911 2.987051 -0.673141
H 2.395400 3.521785 -1.794705
H -1.966004 3.290216 -1.829524
H -1.942674 3.590252 -0.068215
H -1.465567 4.872708 -1.205794
H -2.448313 1.304312 -1.123821
H -1.390540 -0.225081 1.946649
H -3.086618 -3.076438 1.046718
H -2.970959 -2.003631 2.472412
H -3.684813 0.280043 2.403125
H -4.769098 0.647121 1.046824
H -5.179801 -1.787070 -0.009596

H -4.626234 -0.473208 -1.055182
H -2.862584 -2.025000 -1.576855

7*S*9*S*2"*S*-**11**-c1, ΔG = 0.9086 kcal/mol, population = 7.14 %

C 1.958075 0.294299 -0.909588
C 1.665844 1.606418 -0.523466
C 0.356836 1.930938 -0.102919
C -0.629495 0.948615 -0.072500
C -0.271729 -0.370438 -0.440447
C 0.991071 -0.723838 -0.839046
O 3.221925 0.073431 -1.367486
C 3.726630 -1.282032 -1.423603
C 3.795184 -1.962340 -0.085747
C 4.513650 -1.530548 0.954932
C 4.497601 -2.259986 2.268317
C 5.356892 -0.287449 0.922820
C 2.720816 2.677369 -0.555029
O 0.193178 3.238141 0.238619
C -0.951006 3.655000 0.989361
C -2.133762 0.993550 0.200818
N -2.552741 -0.214790 -0.450177
C -1.491555 -1.169021 -0.102589
C -3.339500 -2.336554 0.021579
O -2.009576 -2.377098 -0.598623
O -2.535636 0.797623 1.599945
C -3.589887 -0.194504 1.647119
C -3.697605 -0.813585 0.214012
C -4.997091 -0.419706 -0.481528
O -4.961761 -0.738153 -1.866281
H 1.221256 -1.744643 -1.100284
H 3.127544 -1.865938 -2.124656
H 4.715012 -1.156133 -1.864210
H 3.198242 -2.860381 0.031442
H 3.854342 -3.139519 2.237329
H 5.507550 -2.576562 2.546110
H 4.145547 -1.601677 3.068662
H 5.484193 0.112206 -0.080710
H 4.894516 0.494752 1.533307
H 6.341476 -0.484682 1.355117
H 2.702743 3.262237 0.365177
H 3.706765 2.238651 -0.680128
H 2.552232 3.376925 -1.378873
H -1.198597 2.933723 1.768636
H -0.676848 4.604481 1.443082
H -1.812110 3.808483 0.338197
H -2.643048 1.874613 -0.181695
H -1.430134 -1.293936 0.991809
H -4.026632 -2.834400 -0.655407
H -3.305030 -2.856902 0.980279
H -3.326503 -0.931840 2.404837
H -4.527945 0.286509 1.933523
H -5.840592 -0.955401 -0.043819
H -5.168232 0.653680 -0.335173
H -4.093023 -0.447363 -2.182584

7*S*9*S*2"*S*-**11**-c64, ΔG = 1.2255 kcal/mol, population = 4.18 %

C 1.945705 0.395899 -0.809578
C 1.603816 1.689273 -0.373029
C 0.280645 1.933988 0.021190
C -0.655972 0.915105 0.000693
C -0.276584 -0.367917 -0.432633
C 1.011545 -0.651416 -0.826929
O 3.235387 0.250499 -1.214117
C 3.766535 -1.077641 -1.437598
C 3.798572 -1.941087 -0.207932
C 4.451814 -1.648172 0.920155
C 4.397858 -2.565545 2.108979
C 5.256649 -0.394638 1.114693
C 2.652951 2.763682 -0.323525
O -0.097923 3.182005 0.473339
C -0.420627 4.113571 -0.571258
C -2.138930 0.936621 0.334489
N -2.568576 -0.239140 -0.372554
C -1.482005 -1.202354 -0.117751
C -3.312214 -2.396809 0.002237
O -2.005295 -2.384011 -0.667429
O -2.477513 0.651782 1.732608
C -3.514824 -0.359179 1.763120
C -3.677790 -0.892954 0.300601
C -5.010472 -0.476957 -0.314600
O -5.029573 -0.708411 -1.716909
H 1.287174 -1.647099 -1.136456
H 3.201247 -1.563146 -2.235428
H 4.767619 -0.878746 -1.818404
H 3.231636 -2.864831 -0.253560
H 5.403552 -2.892193 2.390380

H 3.984327 -2.043772 2.977646
H 3.787575 -3.447487 1.914331
H 5.394782 0.167167 0.193837
H 4.758903 0.264043 1.833478
H 6.236694 -0.635550 1.535414
H 2.307667 3.610609 0.265243
H 3.572067 2.377856 0.117781
H 2.907200 3.121339 -1.324995
H -1.257143 3.744889 -1.170017
H -0.703190 5.041971 -0.080498
H 0.438750 4.290008 -1.221452
H -2.657958 1.846233 0.034367
H -1.385340 -1.391393 0.964607
H -4.018700 -2.860792 -0.678869
H -3.237410 -2.974192 0.925300
H -3.208245 -1.137281 2.461766
H -4.446055 0.087663 2.118905
H -5.827501 -1.052115 0.123425
H -5.188944 0.582286 -0.093892
H -4.177211 -0.388417 -2.049182

7*S*9*S*2"*S*-**11**-c34, ΔG = 1.3429 kcal/mol, population = 3.43 %

C 1.989172 0.026572 -1.405712
C 1.893673 1.274806 -0.800735
C 0.687125 1.631451 -0.144310
C -0.383986 0.746022 -0.132914
C -0.229997 -0.515569 -0.766229
C 0.928118 -0.892494 -1.388565
O 3.162922 -0.339831 -2.029854
C 3.957177 -1.305710 -1.266417
C 4.517526 -0.697058 -0.024032
C 3.992408 -0.764360 1.204169
C 4.615380 -0.005322 2.342732
C 2.755903 -1.534311 1.574139
C 3.033960 2.251529 -0.813963
O 0.728118 2.859500 0.437424
C -0.307351 3.285784 1.326197
C -1.821006 0.860308 0.385003
N -2.459427 -0.169120 -0.383084
C -1.470759 -1.255153 -0.378713
C -3.396674 -2.270546 -0.153019
O -2.183806 -2.304923 -0.979761
O -2.024142 0.442682 1.776457
C -3.161611 -0.453280 1.831269
C -3.551504 -0.785204 0.351848
C -4.890031 -0.165182 -0.036717
O -5.100754 -0.221176 -1.441139
H 1.041192 -1.864028 -1.849908
H 3.340965 -2.181185 -1.060192
H 4.747592 -1.596545 -1.955611
H 5.396651 -0.076755 -0.168930
H 5.499682 0.547513 2.026103
H 3.895485 0.704745 2.762241
H 4.898039 -0.681604 3.155034
H 2.946051 -2.154783 2.454124
H 1.953092 -0.840499 1.842515
H 2.385347 -2.171397 0.775557
H 3.868829 1.852257 -1.381518
H 2.726263 3.202159 -1.254728
H 3.372882 2.466756 0.201495
H 0.104587 4.130906 1.872118
H -1.187164 3.614767 0.772275
H -0.580409 2.495835 2.026722
H -2.283514 1.833758 0.245524
H -1.265620 -1.582421 0.654733
H -4.225991 -2.575115 -0.783655
H -3.280785 -2.960736 0.684371
H -2.868184 -1.336726 2.397357
H -3.987359 0.040646 2.348100
H -5.710372 -0.704371 0.439253
H -4.918675 0.871842 0.318896
H -4.264204 0.053168 -1.846169

7*S*9*S*2"*S*-**11**-c32, ΔG = 1.9396 kcal/mol, population = 1.25 %

C 2.133077 0.140241 1.221322
C 1.984235 1.299778 0.468850
C 0.699948 1.633706 -0.034813
C -0.379046 0.791817 0.206610
C -0.176065 -0.359483 1.013237
C 1.044607 -0.689289 1.534562
O 3.378338 -0.207895 1.699566
C 4.005660 -1.306767 0.961500
C 4.390215 -0.894229 -0.420608
C 3.675060 -1.080119 -1.535449
C 4.140279 -0.508157 -2.845654
C 2.361553 -1.806741 -1.606618

C 3.145515 2.202382 0.166471
O 0.680153 2.789981 -0.749100
C -0.556292 3.372758 -1.168029
C -1.827970 0.789722 -0.291379
N -2.179322 -0.582605 -0.066150
C -1.560776 -0.880506 1.232919
C -3.494404 -1.903613 1.301787
O -2.064575 -2.161620 1.510879
O -2.782584 1.540256 0.530662
C -3.943340 0.706662 0.771431
C -3.589060 -0.728038 0.256051
C -4.397022 -1.105845 -0.981650
O -3.871832 -2.267839 -1.609448
H 1.187439 -1.561303 2.158029
H 4.881740 -1.554899 1.557525
H 3.329704 -2.162618 0.969364
H 5.306923 -0.317064 -0.492853
H 5.089103 0.018751 -2.745475
H 4.255065 -1.294729 -3.597404
H 3.395830 0.192702 -3.236986
H 2.100134 -2.323786 -0.687132
H 1.557637 -1.096956 -1.825396
H 2.374575 -2.534029 -2.422917
H 3.343508 2.237181 -0.907119
H 2.933654 3.224916 0.485094
H 4.040494 1.850309 0.670331
H -0.953022 2.859487 -2.044631
H -1.292635 3.362641 -0.363729
H -0.320906 4.400123 -1.434497
H -1.956043 1.088593 -1.328329
H -1.971034 -0.218757 2.014226
H -3.935964 -2.820573 0.924377
H -3.952418 -1.623648 2.251987
H -4.158074 0.729065 1.839304
H -4.800185 1.113314 0.229657
H -5.431085 -1.320243 -0.708129
H -4.402897 -0.258407 -1.677403
H -2.910729 -2.145158 -1.632614

7*S*9*S*2"*S*-**11**-c33, ΔG = 2.0520 kcal/mol, population = 1.03 %

C 2.302239 0.563697 0.875673
C 1.956144 1.765380 0.263978
C 0.598560 2.004095 -0.067897
C -0.359429 1.034817 0.205247
C 0.043474 -0.152697 0.870142
C 1.342648 -0.397162 1.224445
O 3.623507 0.294271 1.165730
C 4.374376 -0.256962 0.032903
C 3.903158 -1.621236 -0.346809
C 3.016690 -1.915318 -1.304617
C 2.562702 -3.331615 -1.522655
C 2.358855 -0.907478 -2.203548
C 2.986505 2.809535 -0.060776
O 0.382209 3.207903 -0.662220
C -0.945507 3.711516 -0.832067
C -1.843909 0.914213 -0.153626
N -2.023366 -0.504514 -0.041502
C -1.248659 -0.848164 1.158734
C -3.044613 -2.094602 1.289098
O -1.581102 -2.199817 1.344898
O -2.782713 1.469178 0.826859
C -3.814760 0.486562 1.090013
C -3.369380 -0.840896 0.390659
C -4.256998 -1.182036 -0.802333
O -3.687819 -2.214086 -1.596581
H 1.647297 -1.305046 1.723896
H 4.317452 0.447456 -0.797452
H 5.401467 -0.281908 0.391978
H 4.277415 -2.431831 0.271514
H 3.055590 -4.026456 -0.842741
H 1.480996 -3.408746 -1.372744
H 2.759756 -3.649657 -2.550732
H 2.429328 -1.224305 -3.247516
H 1.293734 -0.841103 -1.960711
H 2.778717 0.091153 -2.116262
H 3.131793 2.903583 -1.140378
H 2.667808 3.788541 0.299476
H 3.940824 2.559718 0.395910
H -1.425390 3.262925 -1.702389
H -1.552071 3.540138 0.057779
H -0.835769 4.780212 -0.999331
H -2.108260 1.292801 -1.137566
H -1.646219 -0.314181 2.038191
H -3.417533 -3.015605 0.852038
H -3.435035 -1.967383 2.300208
H -3.912417 0.380468 2.169918
H -4.763867 0.840666 0.681785

H -5.232608 -1.531678 -0.461736
H -4.414796 -0.275850 -1.399503
H -2.751509 -1.986078 -1.698108

7*S*9*S*2"*S*-**11**-c45, ΔG = 2.2195 kcal/mol, population = 0.78 %

C 2.089039 0.355282 -1.341329
C 1.821959 1.655009 -0.921827
C 0.565097 1.946449 -0.333209
C -0.391647 0.944078 -0.215199
C -0.066478 -0.362711 -0.665760
C 1.147672 -0.675373 -1.214561
O 3.320695 0.045134 -1.880094
C 4.351370 -0.235917 -0.875612
C 4.071118 -1.484195 -0.106709
C 3.459363 -1.563398 1.080423
C 3.165789 -2.899099 1.704755
C 2.968911 -0.386469 1.875583
C 2.836493 2.754036 -1.065886
O 0.444418 3.237436 0.076030
C -0.696889 3.670798 0.818699
C -1.839691 0.940324 0.285668
N -2.327159 -0.262671 -0.324724
C -1.205328 -1.197378 -0.172327
C -2.983997 -2.414507 0.207463
O -1.767651 -2.406139 -0.614515
O -2.005856 0.701836 1.723603
C -3.017098 -0.318944 1.910577
C -3.339279 -0.905949 0.495995
C -4.741850 -0.530209 0.027951
O -4.920083 -0.809969 -1.353974
H 1.404561 -1.679045 -1.519211
H 5.262664 -0.333554 -1.462533
H 4.452425 0.634677 -0.227492
H 4.338036 -2.409879 -0.607981
H 3.522880 -3.723957 1.088311
H 3.628270 -2.976219 2.693277
H 2.087837 -3.018910 1.853118
H 3.290258 0.572664 1.477790
H 1.874601 -0.386868 1.893542
H 3.304064 -0.462074 2.913470
H 3.213821 3.074885 -0.091225
H 3.676232 2.422676 -1.671415
H 2.390591 3.632436 -1.534384
H -0.927183 2.981927 1.632316
H -0.427266 4.640719 1.228902
H -1.565041 3.787795 0.168964
H -2.424911 1.813617 0.011428
H -0.971807 -1.346846 0.895556
H -3.758670 -2.912983 -0.366719
H -2.787705 -2.956902 1.133884
H -2.619374 -1.066951 2.595627
H -3.907133 0.130702 2.356184
H -5.491169 -1.102053 0.577177
H -4.913842 0.533067 0.233919
H -4.119254 -0.488246 -1.794899
